# Ladderization: A Powerful Strategy for Controlling Supramolecular Helical Ordering, Amplifying Asymmetry, and Emerging and Enhancing Circularly Polarized Luminescence

**DOI:** 10.1002/smll.74040

**Published:** 2026-06-04

**Authors:** Tomoyuki Ikai, Koki Nishino, Nichito Kawabata, Kosuke Oki, Mitsuo Hara, Yukikazu Takeoka, Eiji Yashima

**Affiliations:** ^1^ Department of Molecular and Macromolecular Chemistry Graduate School of Engineering Nagoya University Nagoya Japan; ^2^ Precursory Research For Embryonic Science and Technology (PRESTO) Japan Science and Technology Agency (JST) Kawaguchi Saitama Japan; ^3^ Department of Chemical Engineering National Tsing Hua University Hsinchu Taiwan P. R. China

**Keywords:** alkyne benzannulations, amplification of asymmetry, circularly polarized luminescence, ladder polymers, self‐assembly

## Abstract

We report the synthesis of optically‐active π‐conjugated coplanar ladder polymers with enantiopure side groups that enable helical ordering‐induced circular dichroism (CD) and circularly polarized luminescence (CPL) in a specific solvent mixture and in the film state, whereas the corresponding linear polymer precursor showed a negligible CD and CPL in any state, demonstrating the key role of the coplanar ladder framework in forming well‐ordered supramolecular chiral aggregates. The CPL activities of the pristine films of the chiral ladder polymers were significantly enhanced after solvent vapor exposure, leading to a luminescence dissymmetry factor exceeding 0.1, which arises from the supramolecular helical aggregation through cholesteric ordering. This methodology for enhancing the CPL was versatile and highly effective in generating a comparably intense CPL even when the films contained up to 75 mol% of an achiral analogue of the chiral ladder polymer, demonstrating strong amplification of the asymmetry. Furthermore, the CPL color of the film could be readily tuned by Förster resonance energy transfer to an achiral ladder polymer with a backbone structure different from that of the chiral ladder polymer.

## Graphical abstract

π‐Conjugated chiral ladder polymers with enantiopure side groups exhibit helical ordering‐induced circularly polarized luminescence (CPL) in poor solvents and in the film state. Vapor‐annealed films show a particularly strong CPL with *g*_lum_ values of > 0.1. Blending chiral/achiral ladder polymers markedly amplifies the asymmetry in the film. The CPL color of the blended films can be tuned by combining asymmetry amplification with energy transfer.

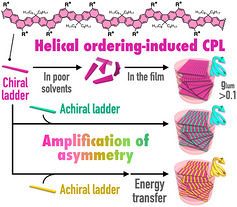

## 1 Introduction

π‐Conjugated polymers are a prominent class of functional materials with widespread applications in electronic and optoelectronic devices as well as chemical sensors due to their unique optical, electronic, and guest‐responsive properties [1, 2, 3, 4, 5]. Their properties and functions are significantly influenced not only by their primary and secondary structures, but also by the interchain packing arrangements in the aggregate and solid states [6, 7, 8]. Therefore, controlling the interchain interactions and hence the morphology of aggregation is a key factor in maximizing the material and device performance.

Optically‐active π‐conjugated polymers (e.g., polythiophenes [9, 10, 11, 12, 13, 14], polyfluorenes [15, 16, 17], poly(*p*‐phenylenevinylene)s [18, 19, 20, 21], poly(*p*‐phenylene)s [22, 23], poly(*p*‐phenyleneethynylene)s [24, 25]) carrying enantiopure side groups generally showed barely detectable chiroptical responses in the molecularly dispersed state in solution. On the other hand, in poor solvents, particularly at low temperatures, or in the film state, these polymers formed interpolymer supramolecular helical assemblies with a controlled handedness, resulting in a strong circular dichroism (CD) and/or circularly polarized luminescence (CPL) in the π–π* transition of the main chain chromophore regions [26, 27, 28, 29, 30, 31, 32, 33, 34, 35, 36, 37, 38]. Hence, supramolecular helical assemblies of π‐conjugated chiral polymers have attracted considerable attention for chiroptical devices [39, 40, 41, 42, 43, 44], anti‐counterfeiting security [45, 46], biochemical sensing [47, 48, 49], and information storage [50, 51]. However, all such supramolecular systems reported to date have consisted of optically‐active π‐conjugated polymers composed of monomer units connected by single bonds (i.e., linear polymers), which promotes a high conformational freedom of the entire polymer chain [52, 53]. Therefore, predicting the structure of aggregates from the structures of π‐conjugated chiral linear polymers with such conformational fluctuations still remains challenging.

**FIGURE 1:**
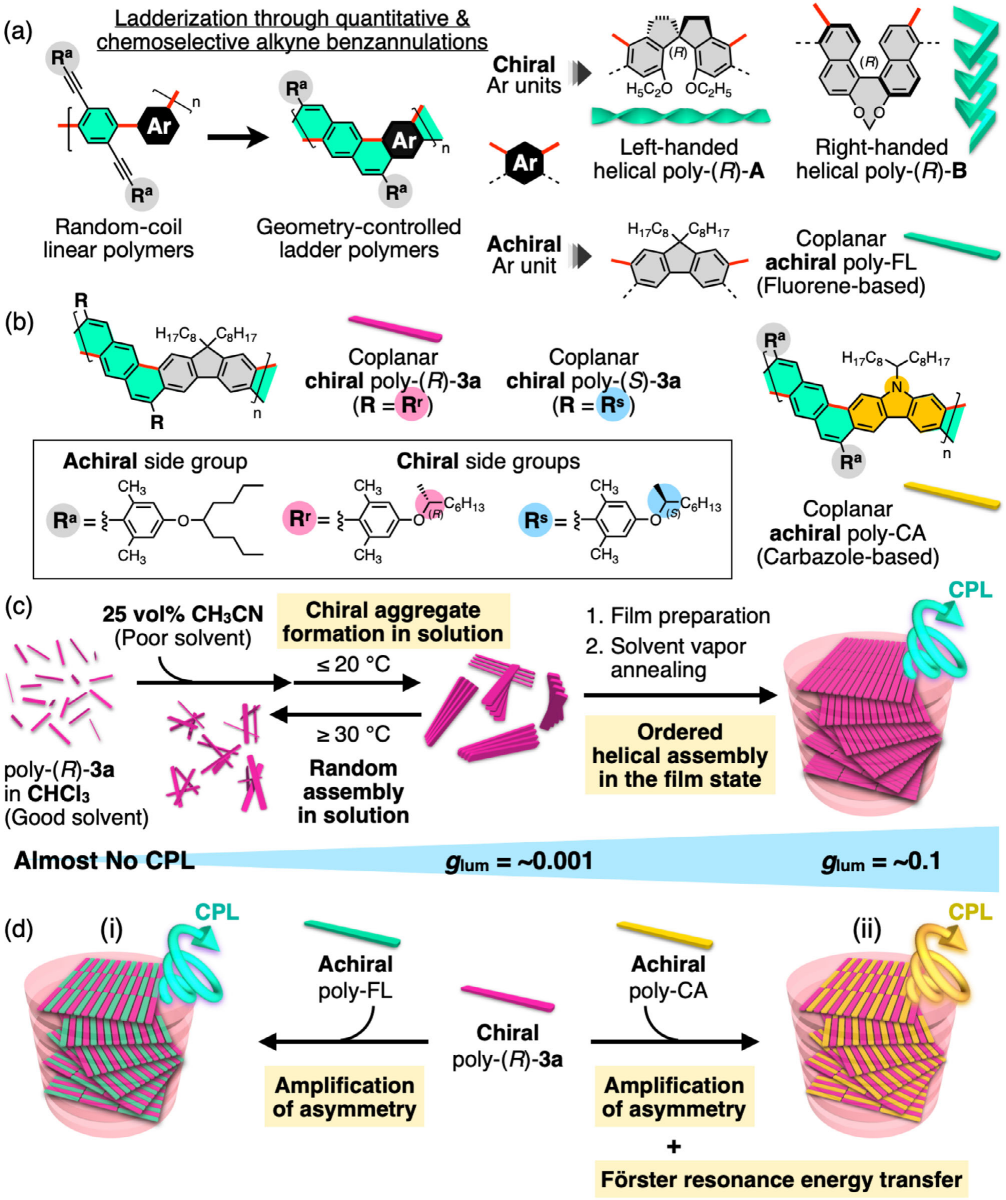
(a) Rational synthesis of the geometry‐controlled ladder polymers with single‐handed helical (poly‐(*R*)‐**A** and poly‐(*R*)‐**B**) and coplanar (poly‐FL) achiral backbone structures through quantitative and chemoselective alkyne benzannulations of the corresponding random‐coil linear polymers with and without chiral aromatic units, respectively. (b) Structures of fluorene‐based optically‐active, π‐conjugated coplanar ladder polymers (poly‐(*R*)‐**3a** and poly‐(*S*)‐**3a**) carrying enantiopure alkoxy side groups and a carbazole‐based achiral π‐conjugated coplanar ladder polymer (poly‐CA) with achiral alkoxy side groups. (c) Schematic illustrations of the chiral aggregate formation of the coplanar chiral ladder polymer (poly‐(*R*)‐**3a**) in solution, followed by more ordered supramolecular helical assembly in the film state, resulting in the enhancement of circularly polarized luminescence (CPL) with a luminescence dissymmetry factor (*g*_lum_) of 0.1. (d) Schematic illustrations of the generation of circularly polarized light accompanied by the amplification of asymmetry in the blended films composed of chiral and achiral ladder polymers without (i) and with (ii) Förster resonance energy transfer (FRET).

The ladder polymers are composed of cyclic repeating units interconnected by two or more covalent bonds [54, 55], hence, the conformational freedom is severely restricted [56, 57, 58, 59, 60, 61], which enables the structural designability in a predictable manner, thus making ladder polymers a powerful platform for the precise control of the backbone geometry [62]. In fact, we recently succeeded in the synthesis of a series of well‐defined helical ladder polymers without detectable structural defects [63, 64, 65, 66, 67, 68] using alkyne benzannulation [69, 70] and its modified method [64]. The incorporation of chiral units into the shape‐persistent ladder‐type backbone allowed the precise control of the spiral structures, resulting in the rational synthesis of single‐handed helical ribbon‐like and tubular ladder polymers, such as poly‐(*R*)‐**A** [64] and poly‐(*R*)‐**B** [67], respectively (Figure 1a). Fully π‐conjugated, achiral coplanar ladder polymers (e.g., poly‐FL) with highly rigid, one‐dimensionally extended π‐conjugated backbones were also successfully synthesized using achiral monomer units as the building blocks (Figure 1a) [64]. We envisioned that highly‐ordered, supramolecular helical assemblies could be generated using coplanar π‐conjugated chiral ladder polymers due to their extremely limited conformational freedom. However, the helical self‐assembly of π‐conjugated ladder polymers is an unexplored field but with significant potential for creating high‐performance chiral materials.

To this end, we synthesized optically‐active, π‐conjugated coplanar ladder polymers carrying enantiopure alkoxy side groups by alkyne benzannulation (Figure 1b) and investigated the impact of a unique ladder framework on supramolecular helical aggregate formation and chiroptical enhancement in both the solution and film states after solvent vapor exposure (Figure 1c). Furthermore, we, for the first time, demonstrated the amplification of asymmetry (sergeants‐and‐soldiers (S&S) effect) [71, 72] in the blended films composed of chiral and achiral ladder polymers, resulting in the generation of circularly polarized light with a luminescence dissymmetry factor (|*g*_lum_|) greater than 0.1, which is 100‐fold higher than that of the chiral ladder polymer aggregates in solution. CPL color modulation was also achieved by combining the S&S effect with Förster resonance energy transfer (FRET) [73, 74, 75, 76, 77] between the chiral and achiral ladder polymers with different backbones (Figure 1d). We also highlighted the potential superiority of the ladder polymers over the corresponding linear polymers in achieving ordered helical aggregation, asymmetry amplification, and the emergence of a strong CPL.

## 2 Results and Discussion

**SCHEME 1:**
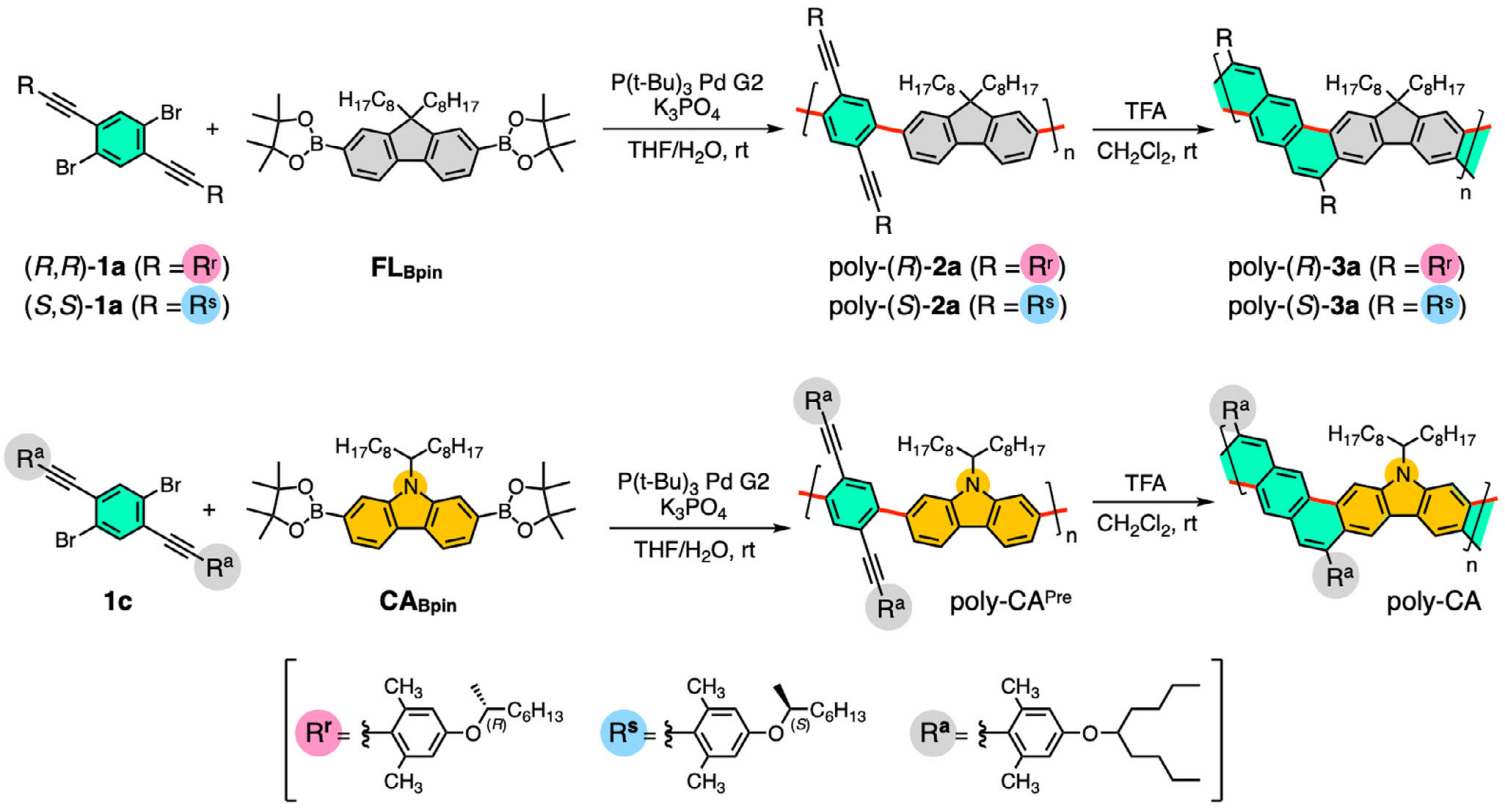
Synthesis of poly‐(*R*)‐**3a**, poly‐(*S*)‐**3a**, and poly‐CA.

Novel enantiopure 1,4‐dibromobenzenes ((*R*,*R*)‐ and (*S*,*S*)‐**1a** in Scheme 1) carrying (*R*)‐ and (*S*)‐4‐alkoxy‐2,6‐dimethylphenylethynyl side groups (R^r^ and R^s^, respectively) at the 2,5‐positions of the benzene ring were synthesized by the Sonogashira–Hagihara cross‐coupling of the corresponding enantiopure phenylacetylenes with 1,4‐dibromo‐2,5‐diiodobenzene (Scheme S1 and Figure S1). The resulting (*R*,*R*)‐ and (*S*,*S*)‐**1a** were then copolymerized with the achiral fluorene‐based diboronic acid ester monomer (**FL_Bpin_**) by the Suzuki–Miyaura cross‐coupling, affording optically‐active precursor polymers (poly‐(*R*)‐**2a** and poly‐(*S*)‐**2a**, respectively) with the number‐average molar masses (*M*_n_) of more than 3.04 × 10^4^, as estimated by size‐exclusion chromatography (SEC) (Scheme 1; Scheme S3 and entries 1 and 2 in Table S1). The precursor polymers were then treated with trifluoroacetic acid (TFA) to convert them into the corresponding ladder polymers by the acid‐promoted alkyne benzannulation according to a previously reported method (Scheme 1; Scheme S4) [64]. The alkyne groups were completely consumed, as confirmed by an IR analysis (Figure S2a–d), and the resulting poly‐(*R*)‐**3a** and poly‐(*S*)‐**3a** showed well‐resolved ^1^H NMR spectra (Figure S3a–d), virtually identical to that of the previously reported fluorene‐based achiral ladder polymer (poly‐FL) with achiral alkoxy side groups (R^a^) [64]. These results indicated the defect‐free formation of the desired coplanar ladder structures, which was also supported by the matrix‐assisted laser desorption‐ionization time‐of‐flight mass analysis (Figure S6a). A carbazole‐based achiral ladder polymer (poly‐CA) was also synthesized through ladderization of the precursor polymer, poly‐CA^Pre^ (Scheme 1, Schemes S3 and S4, and entry 3 in Table S1; for the IR, NMR, and MALDI‐TOF‐MS spectra of poly‐CA^Pre^ and poly‐CA, see Figures S2e,f, S3e,f, S4, S5, and S6b).

**FIGURE 2:**
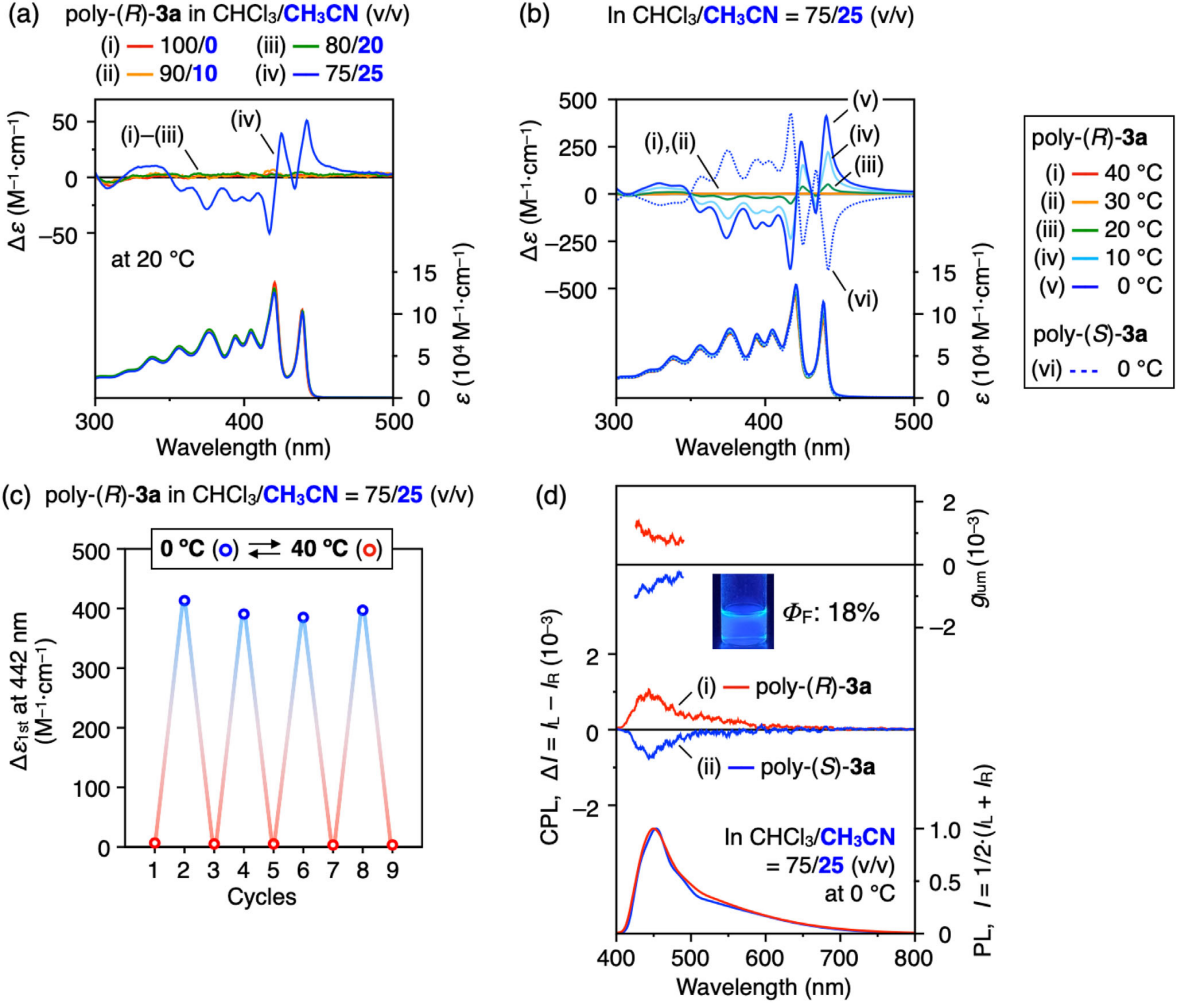
(a) Absorption and CD spectra of poly‐(*R*)‐**3a** in chloroform/acetonitrile (100/0 (i), 90/10 (ii), 80/20 (iii), and 75/25 (iv), v/v) at 20°C. The acetonitrile content could not exceed 30 vol% due to the precipitation of poly‐(*R*)‐**3a**. Dynamic light scattering (DLS) measurements showed that poly‐(*R*)‐**3a** exhibited insufficient light scattering in chloroform at 10°C–40°C, indicating that poly‐(*R*)‐**3a** does not form aggregates in chloroform within this temperature range and remains in a molecularly dispersed state (Table S2). (b) Absorption and CD spectra of poly‐(*R*)‐**3a** (i–v) and poly‐(*S*)‐**3a** (vi) in chloroform/acetonitrile (75/25, v/v) at different temperatures (40 (i), 30 (ii), 20 (iii), 10 (iv), and 0 (v, vi) °C). For the corresponding variable‐temperature absorption and CD spectra in chloroform/acetonitrile (100/0, 90/10, and 80/20, v/v), see Figure S7a,c,d. (c) Reversible change in CD intensity (Δ*ε*_first_ at 442 nm) of poly‐(*R*)‐**3a** in chloroform/acetonitrile (75/25, v/v) upon temperature variation between 40 (red circles) and 0 (blue circles) °C. Measurements were performed immediately after each temperature change. (d) Normalized PL (bottom), CPL (middle), and *g*_lum_ (top) spectra (*λ*_ex_ = 300 nm) of poly‐(*R*)‐**3a** (i) and poly‐(*S*)‐**3a** (ii) in chloroform/acetonitrile (75/25, v/v) at 0°C. The *g*_lum_ values are defined as 2(*I*_L_—*I*_R_)/(*I*_L_ + *I*_R_), where *I*_L_ and *I*_R_ are the PL intensities of the left‐ and right‐handed circularly polarized light, respectively. The inset shows a photograph of poly‐(*R*)‐**3a** in chloroform/acetonitrile (75/25, v/v) under irradiation at 365 nm. Fluorescence quantum yield (*Φ*_F_) is also shown. [Repeating units of polymer] = 0.10 mm.

The poly‐(*R*)‐**3a** showed no CD in good solvents, such as chloroform and tetrahydrofuran as well as in chloroform/acetonitrile (80/20 and 90/10, v/v) mixtures, at 0°C–20°C (Figure 2a‐i–iii; Figure S7), but a clear Cotton effect was observed in the π–π* transition of the poly‐(*R*)‐**3a** backbone in chloroform containing 25 vol% acetonitrile as a poor solvent at 20°C (Figure 2a‐iv). The molar CD at the first Cotton effect (Δ*ε*_first_) at 442 nm significantly increased at lower temperatures, exceeding 400 at 0°C (Figure 2b‐i–v). The CD intensity of poly‐(*R*)‐**3a** in chloroform/acetonitrile (75/25, v/v) dramatically decreased upon 10‐ and 100‐fold dilutions (Figure S8). This result indicated that the CD activity of poly‐(*R*)‐**3a** observed in a chloroform/acetonitrile mixture below 20°C arose from the ordered helical aggregate formation of the chiral ladder polymers (Figure 1c, middle). On the other hand, no significant differences were observed in the absorption spectra of poly‐(*R*)‐**3a** with variations in the solvent (Figure 2a), concentration (Figure S8), and temperature (Figure 2b), indicating that interchain π–π stacking is minimal even in the aggregated state. This is probably due to the bulky aromatic side groups attached to the rigid π‐conjugated coplanar backbone (see below and Figure S19).

The aggregate formation was further confirmed by the variable‐temperature dynamic light scattering (DLS) measurements in chloroform/acetonitrile (75/25, v/v) at 10°C–40°C (Figure S9 and Table S2). Poly‐(*R*)‐**3a** showed a bimodal size distribution with hydrodynamic diameters (*D*_H_) centered at ca. 15–18 and 122 nm at 30°C and 40°C, likely corresponding to slightly and highly aggregated structures, respectively (Figure S9a‐iii, iv). The proportion of the large aggregates increased with the decreasing temperature, reaching an average *D*_H_ value of 204 nm with a polydispersity index (PDI) of 0.145 at 10°C (Figure S9a‐i and Table S2). Because poly‐(*R*)‐**3a** showed almost no Cotton effect in chloroform/acetonitrile (75/25, v/v) above 30°C (Figure 2b‐i, ii), the aggregates formed at 30°C and 40°C are likely composed of CD‐inactive randomly assembled disordered structures (Figure 1c (left) and Figure S9a‐iii, iv), different from the CD‐active highly‐ordered, helically assembled structures formed below 20°C (Figures 1c (middle) and 2b‐iii–v and Figure S9a‐i,ii). The CD intensity of poly‐(*R*)‐**3a** in chloroform/acetonitrile (75/25, v/v) remained unchanged after storage at 0°C for 24 h (Figure S10), indicating that the helical aggregate formation was instantaneously completed. Therefore, the CD‐active helical aggregate formation can be rapidly and reversibly switched (off and on) by repeating the heating (40°C) and cooling (0°C) cycle, respectively (Figure 2c).

As expected from the CD study, poly‐(*R*)‐**3a** showed almost no CPL in chloroform at 0°C (Figure S11), but displayed a clear CPL signal in chloroform/acetonitrile (75/25, v/v) at 0°C due to the helical aggregate formation, with a |*g*_lum_| value of 8 × 10^−4^ (Figure 2d‐i). To the best of our knowledge, this is the first example of the helical ordering‐induced CPL derived from an optically‐active ladder polymer. The enantiomeric poly‐(*R*)‐**3a** and poly‐(*S*)‐**3a** displayed the mirror‐image CD and CPL spectra in chloroform/acetonitrile (75/25, v/v) at 0°C (Figure 2b‐v,vi,d). In sharp contrast, no clear CD and CPL signals were observed for the precursor linear polymer (poly‐(*R*)‐**2a**) in chloroform/acetonitrile (75/25, v/v) at 0°C (Figure S12), which is consistent with the previous report that an optically‐active poly(fluorene‐*alt*‐*p*‐phenylene) bearing enantiopure side groups on the phenylene units exhibited a very weak Cotton effect even in thin films [78]. Based on the fact that poly‐(*R*)‐**2a** formed CD‐inactive disordered aggregates in chloroform/acetonitrile (75/25, v/v) below 20°C, as confirmed by a DLS analysis (Figure S9b and Table S2), the highly‐restricted conformational freedom of chiral ladder polymers most likely plays a critical role in the formation of well‐ordered, supramolecular helical aggregates that exhibit a strong optical activity. To clarify how the formation of helical aggregate depends on the *M*_n_ and *M*_w_/*M*_n_ values of poly‐(*R*)‐**3a** chains, we used SEC to fractionate the as‐synthesized poly‐(*R*)‐**3a** (*M*_n_ = 3.30 × 10^4^; *M*_w_/*M*_n_ = 4.6) into four fractions (**f1**–**f4**) with different *M*_n_ values (1.13 × 10^4^–16.2 × 10^4^) and narrower molar‐mass dispersities (*M*_w_/*M*_n_ ≤ 1.3) (Figure S13a,b). The CD and CPL signals of poly‐(*R*)‐**3a** were found to increase with the *M*_n_ values in chloroform/acetonitrile (75/25, v/v) at 0°C (Figure S13c–f), suggesting that higher‐molar‐mass fractions form more highly‐ordered, helically assembled structures. In addition, the PL spectra varied substantially with the *M*_n_ values (Figure S13d); in particular, the highest‐molar‐mass fraction (**f1**) exhibited a broadened PL profile similar to that observed for the polymer film discussed below, whereas the absorption spectra showed only minor differences among the fractions (Figure S13c). Notably, the chiroptical intensities of the **f3** fraction, which has an *M*_n_ value (2.95 × 10^4^) comparable to that of the unfractionated poly‐(*R*)‐**3a** but a narrower dispersity (1.3), were lower than those before fractionation ((iii) and (v) in Figure S13c,d). These results indicated that the molar‐mass effect is more pronounced than the dispersity effect. However, the subsequent experiments in the film state were carried out using the as‐synthesized poly‐(*R*)‐**3a** due to the difficulty of obtaining sufficiently high‐molar‐mass SEC fractions on a preparative scale.

**FIGURE 3:**
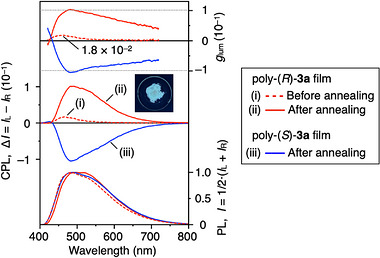
Normalized PL (bottom), CPL (middle), and *g*_lum_ (top) spectra (*λ*_ex_ = 300 nm) of poly‐(*R*)‐**3a** (i, ii) and poly‐(*S*)‐**3a** (iii) in the film state measured at room temperature before (i) and after (ii, iii) annealing in chloroform vapor at 25°C for 1 h. The pristine polymer thin films were prepared by drop‐casting of the corresponding chloroform/acetonitrile (75/25, v/v) solutions (1.0 mm). The inset shows a photograph of the vapor annealed poly‐(*R*)‐**3a** film under irradiation at 365 nm.

**FIGURE 4:**
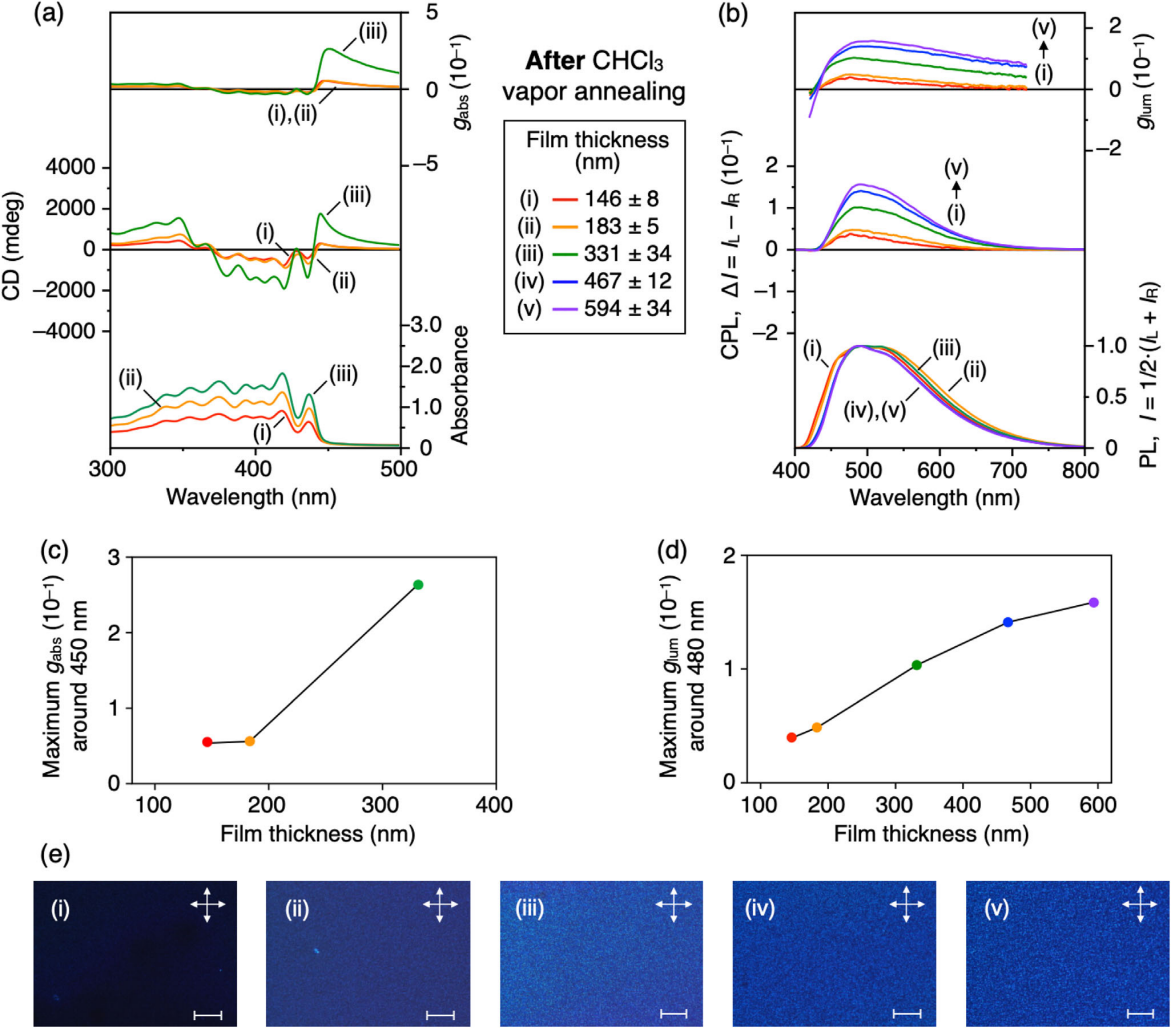
(a, b) Absorption (bottom), CD (middle), and *g*_abs_ (top) spectra (a) and normalized PL (bottom), CPL (middle), and *g*_lum_ (top) spectra (b; *λ*_ex_ = 300 nm) of the poly‐(*R*)‐**3a** films with different thicknesses (146 (i), 183 (ii), 331 (iii), 467 (iv), and/or 594 nm (v)) measured at room temperature after annealing in chloroform vapor at 25°C for 1 h. The pristine polymer thin films were prepared by drop‐casting of the corresponding chloroform/acetonitrile (75/25, v/v) solutions (0.40 (i), 0.60 (ii), 1.0 (iii), 1.5 (iv), and/or 2.0 mm (v)). (c, d) Plots of the maximum *g*_abs_ (c; *λ* ≈ 450 nm) and *g*_lum_ (d; *λ* ≈ 480 nm) values of the poly‐(*R*)‐**3a** films versus the film thickness. (e) Polarized optical micrographs of the vapor‐annealed poly‐(*R*)‐**3a** films with different thicknesses taken at room temperature under crossed polarizers. Scale bar: 50 µm. For the corresponding data of the as‐cast films, see Figure S23.

The photoluminescence (PL) spectrum of the poly‐(*R*)‐**3a** film, cast from its chloroform/acetonitrile (75/25, v/v) solution, was broader than that in solution, resulting in a blue‐white emission (Figures 2d‐i and 3‐i). The *g*_lum_ value of the poly‐(*R*)‐**3a** film reached 1.8 × 10^−2^ (Figure 3‐i), which was 20‐fold higher than that in chloroform/acetonitrile (75/25, v/v) at 0°C (Figure 2d‐i). The influence of macroscopic anisotropy on the polymer films investigated in this study was confirmed to be nearly negligible (Figures S14–S20), and virtually no contribution from linear dichroism was observed (Figure S21). The CPL intensity of the polymer film further increased after chloroform vapor exposure for 1 h, resulting in a maximum *g*_lum_ value of over 1.0 × 10^−1^, which is more than 100‐fold higher than that of the aggregate in solution (|*g*_lum_| ≤ 8 × 10^−4^) (Figure 3‐i, ii). The CD intensity of the poly‐(*R*)‐**3a** film also markedly increased upon vapor annealing (Figure S22‐i, ii), while the maximum CD and absorption wavelengths remained roughly unchanged compared to those before vapor exposure and even in chloroform/acetonitrile (75/25, v/v) at 0°C (Figure 2b‐v). Moreover, we observed a pronounced enhancement of the chiroptical properties with the increasing film thickness after vapor annealing (Figure 4a–d), which is characteristic of chiral π‐conjugated polymer films with a macroscopic helical supramolecular organization, that is, cholesteric liquid crystalline (LC) ordering, as reported previously [16, 78, 79, 80]. Specifically, after vapor annealing, the *g*_lum_ values of the poly‐(*R*)‐**3a** films increased with the increasing film thickness, reaching a maximum value of 0.16 at a film thickness of 594 nm (Figure 4b,d). The Kuhn's dissymmetry factor (*g*_abs_) of the poly‐(*R*)‐**3a** films showed a marked increase at a film thickness of 331 nm (Figure 4a,c). CD measurements could not be performed for films with a thickness of 467 nm or greater due to high absorbance. In contrast, the *g*_abs_ and *g*_lum_ values of the as‐cast films showed virtually no dependence on film thickness (Figure S23a–d). In polarized optical microscopy (POM) observations, the vapor annealed poly‐(*R*)‐**3a** films showed a clear granular texture under crossed polarizers, which became more pronounced as the film thickness increased (Figure 4e). The observed textures are similar to those reported for chiral π‐conjugated polymer films with cholesteric LC ordering [78, 81]. No birefringence was observed for the as‐cast films under crossed polarizers prior to vapor annealing (Figure S23e), indicating that the films are largely amorphous and macroscopically isotropic. These results suggest that the vapor annealing process promoted a more ordered and regular supramolecular helical arrangement of the self‐assembled poly‐(*R*)‐**3a** chains within the film, inducing a cholesteric LC ordering in the initially amorphous as‐cast poly‐(*R*)‐**3a** films (Figure 1c, right) and remarkably enhancing the CD and CPL activities in the film state in a thickness‐dependent manner. Because no pronounced angle dependence in the CD and CPL signals was observed upon rotating the films (Figures S14 and S16), the vapor‐annealed films likely possess a multidomain cholesteric LC arrangement, in which the macroscopic anisotropy is averaged out. The vapor annealed poly‐(*S*)‐**3a** film showed CPL and CD as intense as those of the poly‐(*R*)‐**3a** with the opposite sign (Figure 3‐ii, iii and Figure S22‐ii, iii).

To gain insights into the orientation of the polymer chains in the film state, two‐dimensional, wide‐angle X‐ray scattering (2D‐WAXS) analyses were carried out on the poly‐(*R*)‐**3a** film before and after chloroform vapor annealing. No clear reflections were observed before vapor annealing (Figure S24a,b‐i), whereas a distinct scattering signal (2*θ*_z_ = 7.3°) appeared in the out‐of‐plane direction after annealing (Figure S24a,b‐ii), indicating the formation of a periodic ordered structure with the chain‐to‐chain spacing of 1.0–1.4 nm. The molecular modeling study suggested that this periodicity corresponds to the packing structure of the coplanar poly‐(*R*)‐**3a** chains oriented in a face‐to‐face manner (Figure S25a), in which efficient intermolecular π–π stacking is not anticipated due to the bulky aromatic side groups, rather than in an edge‐to‐edge manner (Figure S25b). The nearly complete absence of π‐electron‐mediated intermolecular interactions is consistent with the finding that the absorption region of the vapor annealed poly‐(*R*)‐**3a** film is nearly identical to that of the molecularly dispersed state in chloroform (Figure 2a‐i and Figure S22‐ii), although the film showed a broader spectral profile. Consequently, the coplanar poly‐(*R*)‐**3a** layers are most likely arranged in a one‐handed helical fashion at 1.0–1.4 nm intervals after chloroform vapor exposure, driven by the chiral bias of the enantiopure side groups (Figure 1c, right), similar to the previously reported helically assembled π‐conjugated linear polymers [16, 22, 26, 27, 28, 29, 30, 31, 32, 33, 34, 35, 36, 37, 38, 78]. Again, no CD and CPL were observed for poly‐(*R*)‐**2a** in the film state even after chloroform vapor exposure (Figure S26).

**FIGURE 5:**
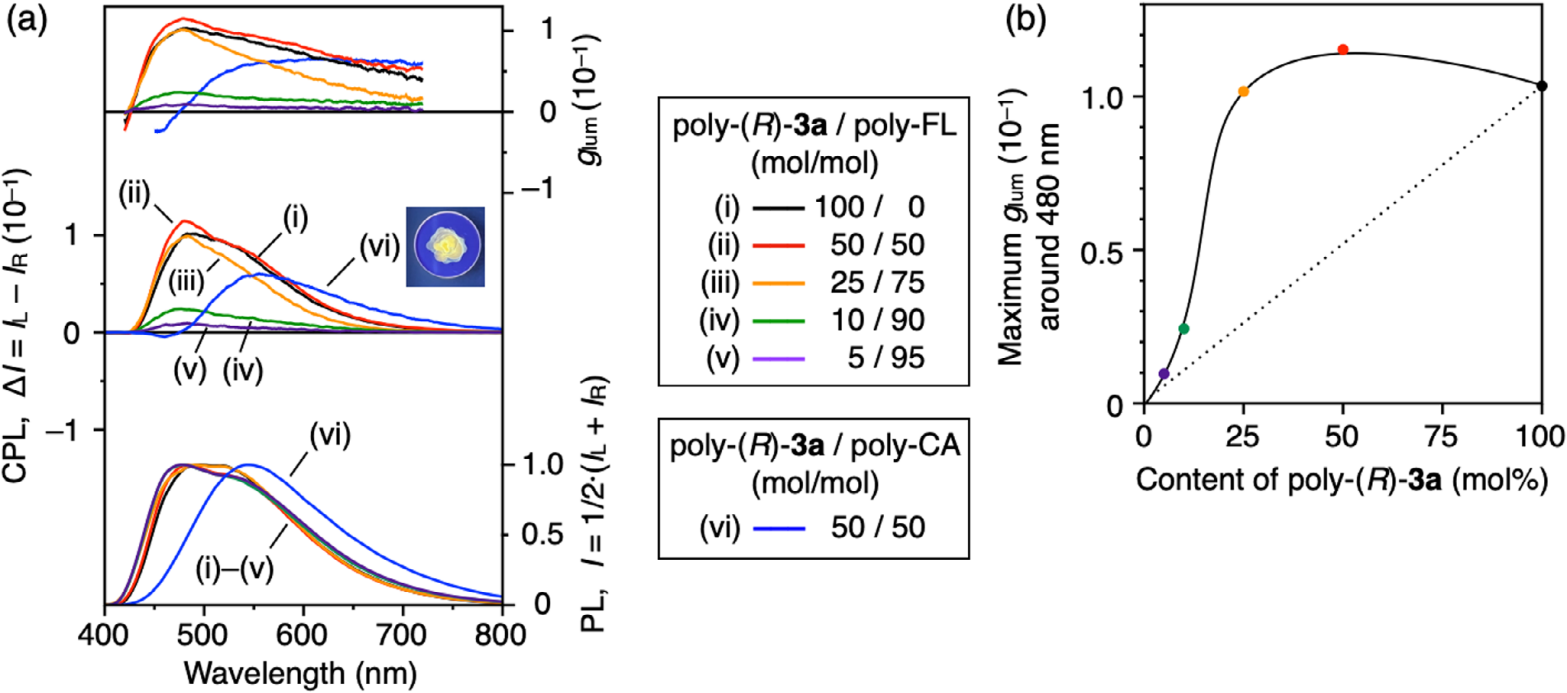
(a) Normalized PL (bottom), CPL (middle), and *g*_lum_ (top) spectra (*λ*_ex_ = 300 nm) of the polymer blended films containing poly‐(*R*)‐**3a** and achiral poly‐FL at different molar ratios (100/0 (i), 50/50 (ii), 25/75 (iii), 10/90 (iv), and 5/95 (v), mol/mol) and the poly‐(*R*)‐**3a/**achiral poly‐CA blend film (50/50, mol/mol) (vi). Measurements were performed at room temperature after annealing in chloroform vapor at 25°C for 1 h. The pristine polymer thin films were prepared by drop‐casting of the corresponding chloroform/acetonitrile (75/25 (i–v) or 100/0 (vi), v/v) solutions (1.0 mm). The inset shows a photograph of the annealed poly‐(*R*)‐**3a/**poly‐CA blended film under irradiation at 365 nm. For the corresponding CD and absorption spectra of the blended films after vapor annealing, see Figure S27. (b) Plots of the maximum *g*_lum_ values around 480 nm of the vapor annealed poly‐(*R*)‐**3a**/poly‐FL blended films versus the content of poly‐(*R*)‐**3a**.

We next investigated the expression of CPL accompanied by the amplification of asymmetry in the blended films composed of chiral (poly‐(*R*)‐**3a**) and achiral (poly‐FL) [64] ladder polymers at varying ratios prepared from their chloroform/acetonitrile (75/25, v/v) solutions (Figure 5a‐i–v). Interestingly, the blended film containing only 25 mol% of the chiral poly‐(*R*)‐**3a** showed a strong CPL with the maximum *g*_lum_ value of 1.0 × 10^−1^ after chloroform vapor annealing (Figure 5a‐iii), comparable to that of the pure poly‐(*R*)‐**3a** film, accompanied by a slight change in the PL spectra (Figure 5a‐i,iii), indicating a strong S&S effect (Figure 5b) [71, 72]. These results suggested that the chiral packing morphology of the chiral/achiral ladder polymers in the blended films appeared to be virtually the same as that of the poly‐(*R*)‐**3a** film (Figure 1c,d and Figure S25a), as evidenced by 2D‐WAXS (Figure S28a,b‐i). Consequently, the achiral poly‐FL chains likely co‐assemble into one‐handed twisted aggregates guided by the chiral bias of poly‐(*R*)‐**3a**. When either or both of the chiral and achiral ladder polymers in the polymer blended films (50/50, mol/mol) were replaced with the corresponding precursor linear polymers (poly‐(*R*)‐**2a** and poly‐FL^Pre^), the resultant films exhibited a very weak or no CPL (Figure S19). These results unambiguously demonstrated that interchain chiral interactions between π‐conjugated ladder polymers are not only crucial for the emergence and ordering of supramolecular helical structures, but also highly effective for amplifying the asymmetry in the π‐conjugated helical systems.

Notably, when the carbazole‐based ladder polymer (poly‐CA) was incorporated as the achiral component (50 mol%) in the blended film, a pronounced CPL response with a *g*_lum_ value of 0.07 emerged after chloroform vapor annealing in the PL region of the achiral poly‐CA (Figure 5a‐vi). This emission band was red‐shifted by ca. 50 nm relative to that of the fluorene‐based chiral ladder polymer (poly‐(*R*)‐**3a**) (Figure 5a‐i). During UV irradiation, the poly‐(*R*)‐**3a**/poly‐CA blended film exhibited a yellowish PL (Figure 5a‐vi), with a PL spectrum nearly identical to that of the pristine poly‐CA film (Figure S29‐ii, iii), indicating that the observed CPL predominantly originated from the achiral poly‐CA. These results suggested that a remarkable amplification of asymmetry occurred in the blended film composed of the chiral and achiral ladder polymers with different main‐chain structures, facilitated by an efficient FRET [73, 74, 75, 76, 77] from the excited state of poly‐(*R*)‐**3a** to the ground state of poly‐CA. As a result, a color‐tunable and intense CPL can be readily generated by simply casting a mixed solution of the chiral and achiral ladder polymers, followed by chloroform vapor annealing of the resulting film. Although numerous studies have examined CPL induction in mixtures of chiral molecules with achiral molecules or polymers as well as in those of achiral molecules with chiral polymers [82, 83, 84], reports of CPL induction based on intermolecular FRET between the chiral and achiral π‐conjugated polymers remain scarce [85, 86, 87]. Therefore, this finding provides a novel design strategy for developing color‐tunable CPL‐active polymer blended films in a rational and predictable manner.

## 3 Conclusion

In summary, we have developed optically‐active, π‐conjugated coplanar ladder polymers bearing enantiopure side groups by a quantitative and chemoselective alkyne benzannulation strategy. These defect‐free chiral ladder polymers undergo an ordered self‐assembly in a supramolecular chiral manner in poor solvents and in the film state, giving rise to pronounced CD and CPL responses, unlike their flexible linear precursors that exhibited almost no optical activity under any conditions. Chloroform‐vapor annealed films with a multidomain cholesteric LC arrangement showed a particularly strong helical ordering‐induced CPL, with the *g*_lum_ values surpassing 0.1. Remarkably, blending chiral and achiral ladder polymers with either identical or distinct backbones resulted in a substantial amplification of asymmetry in the film. The CPL color of the blended films can be tuned by combining amplification of the asymmetry with an efficient FRET, generating an intense and color‐tunable CPL. The present modular approach, integrating the defect‐free ladderization, supramolecular helical aggregation through cholesteric ordering, and amplification of asymmetry, offers a versatile platform for constructing more diverse and elaborate hierarchical architectures with advanced functionalities reminiscent of biological systems, including selective catalysis and molecular and chiral recognition, signal transduction, and energy conversion.

## Conflicts of Interest

The authors declare no conflicts of interest.

## Supporting information




**Supporting File**: smll74040‐sup‐0001‐SuppMat.pdf.

## Data Availability

The data that support the findings of this study are available from the corresponding author upon reasonable request.
