# Supplementary material for: Ladderization: A Powerful Strategy for Controlling Supramolecular Helical Ordering, Amplifying Asymmetry, and Emerging and Enhancing Circularly Polarized Luminescence
Source: Small. 2026 Jun 4;22(42):e74040. doi: 10.1002/smll.74040 (PMC13410664; doi:10.1002/smll.74040)
Supplement: Supplementary file 1 — Supporting File: smll74040‐sup‐0001‐SuppMat.pdf. [file SMLL-22-e74040-s001.pdf]

Supporting Information for:

Ladderization: A Powerful Strategy for Controlling Supramolecular Helical Ordering, Amplifying Asymmetry, and Emerging and Enhancing Circularly Polarized Luminescence

Tomoyuki Ikai,[1,2,*] Koki Nishino,[1] Nichito Kawabata,[1] Kosuke Oki,[1] Mitsuo Hara,[1] Yukikazu Takeoka,[1] and Eiji Yashima,[1,3,*]

[1] Department of Molecular and Macromolecular Chemistry, Graduate School of Engineering, Nagoya University, Chikusa-ku, Nagoya 464-8603, Japan

[2] Precursory Research for Embryonic Science and Technology (PRESTO), Japan Science and Technology Agency (JST), Kawaguchi, Saitama 332-0012, Japan

[3] Department of Chemical Engineering, National Tsing Hua University, 101, Sec. 2, Kuang-Fu Road, Hsinchu, 30013, Taiwan, R.O.C.

* Correspondence: ikai@chembio.nagoya-u.ac.jp; yashima@chembio.nagoya-u.ac.jp

Table of Contents

1. Instruments and Materials ········ S-3

2. Synthetic Procedures ········ S-5

3. Molecular Modeling of Packing Structures of Poly-(*R*)-**3a** ········ S-15

4. Supporting Data ········ S-16

5. Supporting References ········ S-47

6. ^{1}H and ^{13}C NMR Spectral Data ········ S-48

1. Instruments and Materials

Instruments. The melting points were measured on a Yanako melting point apparatus (Yanako, Kyoto, Japan) and were uncorrected. The NMR spectra were measured using a Varian 500AS (Agilent Technologies, Santa Clara, CA) or a Bruker Ascend 500 (Bruker Biospin, Billerica, MA) spectrometer operating at 500 MHz for ^{1}H and 126 MHz for ^{13}C using tetramethylsilane as the internal standard. The IR spectra were recorded on a JASCO FT/IR-6X spectrophotometer (JASCO, Tokyo, Japan) equipped with a JASCO ATR PRO ONE X attenuated total reflectance attachment (ZnSe prism). The absorption and circular dichroism (CD) spectra in solution were obtained in a 1.0- or 10-mm quartz cell using a JASCO V-750 spectrophotometer and a JASCO J-1500 spectropolarimeter, respectively. The concentrations of the polymers were calculated based on the monomer units. The temperature was controlled with a JASCO ETCS-900 apparatus. The photoluminescence (PL) and circularly polarized luminescence (CPL) spectra in solution were recorded on a JASCO CPL-300 spectrophotometer with a 1.0-mm quartz cell. A scanning rate of 200 nm/min, an excitation slit width of 3000 μm, a monitoring slit width of 3000 μm, a response time of 2 seconds, and a 1 time accumulation were employed for the CPL measurements. Drop-coated films prepared from chloroform/acetonitrile (75/25, v/v) solutions of the polymers (1.0 mM) on quartz substrates with and/or without chloroform vapor annealing at 25 °C for 1 h were used for solid-state absorption, CD, PL, and CPL spectral measurements. Film thicknesses were measured by an Alpha-Step IQ surface profiler (KLA-Tencor, Milpitas, CA). Fluorescence quantum yields were measured with a JASCO FP-8550 spectrofluorometer attached with a JASCO ILF-135 integrating sphere (diameter 120 mm). The dynamic light scattering (DLS) measurements were performed on a Zetasizer Nano ZS spectrometer (Malvern Instruments, Worcestershire, UK) equipped with a He/Ne laser (633 nm). Grazing-incidence wide-angle X-ray scattering (GI-WAXS) measurements were performed with an FR-E X-ray diffractometer equipped with a two-dimensional detector R-axis IV (Rigaku, Tokyo, Japan) involving an imaging plate (Fujifilm, Tokyo, Japan). An X-ray beam (Cu Kα = 0.154 nm, 0.3 mm collimated) was used, and the camera length was set at 300 mm. The polymer films were placed onto a pulse motor stage composed of oblique pulse (ATS-C310-EM, Chuo Precision Industrial, Tokyo, Japan) and Z-pulse (ALV-3005-HM, Chuo Precision Industrial) motors. The incident angle of the X-ray beam was adjusted between 0.18 and 0.22° to the substrate surface using the pulse motors. The size exclusion chromatography (SEC) measurements were performed with a JASCO PU-4580 liquid chromatograph equipped with a JASCO CO-4060 column oven and a JASCO MD-2018 multi-wavelength UV/VIS detector. The number-average molar mass (M_n) and its dispersity (M_w/M_n) were determined at 40 °C using a Tosoh TSKgel GMH$_{HR}$-M (30 cm) SEC column (Tosoh, Tokyo, Japan), and chloroform was used as the eluent at a flow rate of 1.0 mL/min. The molar mass calibration curve was obtained with polystyrene standards (Tosoh). Recycling preparative high-performance liquid chromatography (HPLC) was performed with a JAI LC-7080 liquid chromatograph (JAI, Tokyo, Japan) equipped with a JAI UV-800LA UV detector at room temperature. JAIGEL-1HR and

JAIGEL-2HR (60 cm × 2.0 cm (i.d.)) connected in series were used as columns (JAI), and chloroform was used as the eluent at a flow rate of 9.0 mL/min. The chromatographic separations of enantiomers were performed using HPLC equipped with a JASCO PU-4185 Binary HPLC pump, a JASCO AS-4050 autosampler, and JASCO MD-2018 multi-wavelength UV/VIS and JASCO CD-4095 dual detectors, and a CHIRALPAK IB (DAICEL, Osaka, Japan) was used as a chiral column. The high-resolution mass spectra (HRMS) were recorded on a JEOL JMS-T100GCV (JEOL, Akishima, Japan) and a Bruker compact QTOF (Bruker Daltonics, Billerica, MA) spectrometers with electron impact (EI) and atmospheric pressure chemical ionization (APCI), respectively. The matrix-assisted laser desorption-ionization time-of-flight mass (MALDI-TOF-MS) spectra were measured on a Bruker ultrafleXtreme TOF/TOF mass spectrometer (Bruker Daltonics, Bremen, Germany) using *trans*-2-[3-(4-*tert*-butylphenyl)-2-methyl-2-propenylidene]malononitrile as the ionizing matrix.

Materials. All starting materials and anhydrous solvents were purchased from Sigma-Aldrich (St. Louis, MO), Fujifilm Wako Pure Chemical (Osaka, Japan), Tokyo Kasei (TCI, Tokyo, Japan), Nacalai Tesque, or Kanto Kagaku (Tokyo, Japan) and were used as received. (*S*)-*p*-Toluenesulfonic acid 2-octyl ester,[S1] *rac*-*p*-toluenesulfonic acid 2-octyl ester,[S2] and 1,4-dibromo-2,5-bis[2-[4-[(1-butylpentyl)oxy]-2,6-dimethylphenyl]ethynyl]benzene (**1c**)[S3] were synthesized according to the previously reported methods.

2. Synthetic Procedures

Enantiopure 1,4-dibromobenzene monomers ((*R*,*R*)-**1a** and (*S*,*S*)-**1a**) were synthesized according to Scheme S1.

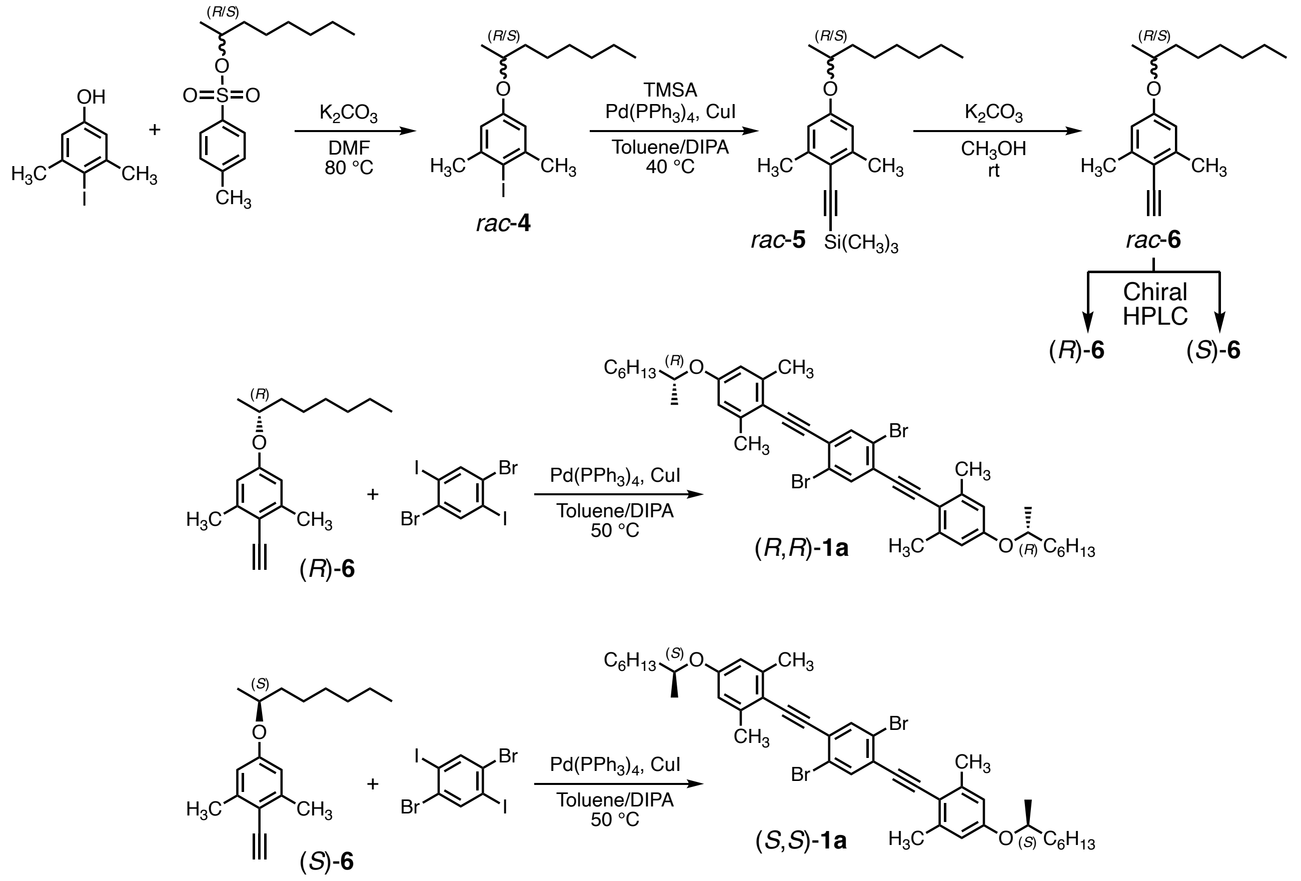

Scheme S1. Synthesis of (*R*,*R*)-**1a** and (*S*,*S*)-**1a**.

Synthesis of *rac*-4. To a mixture of 4-iodo-3,5-dimethylphenol (15.1 g, 60.9 mmol) and potassium carbonate (21 g, 150 mmol) in anhydrous *N*,*N*-dimethylformamide (DMF) (90 mL) was added *rac*-*p*-toluenesulfonic acid 2-octyl ester (20.6 g, 72.4 mmol). After stirring at 80 °C for 16 h, the mixture was cooled to room temperature and diluted with ethyl acetate/*n*-hexane (4/1, v/v). The solution was washed with water, and then dried over Na_2SO_4. The solvents were removed under reduced pressure and the crude product was purified by silica gel chromatography using *n*-hexane as the eluent to give the desired product as a colorless oil (16.4 g, 75% yield). ^{1}H NMR (500 MHz, $CDCl_3$, 25 °C): δ 6.65 (s, 2H, Ar–H), 4.30 (sex, J = 6.1 Hz, 1H, OCH), 2.43 (s, 6H, CH_3), 1.73-1.50 (m, 2H, CH_2), 1.46-1.26 (m, 11H, CH_2, CH_3), 0.88 (t, J = 7.0 Hz, 3H, CH_3). ^{13}C NMR (126 MHz, $CDCl_3$, 25 °C): δ 157.80, 142.78, 114.74, 96.60, 73.85, 36.47, 31.79, 29.71, 29.25, 25.49, 22.60, 19.72, 14.08.

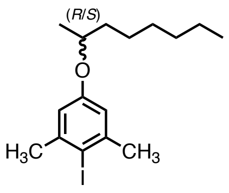

rac-**4**

Synthesis of *rac*-5. To a mixture of *rac*-**4** (16.3 g, 45.2 mmol), copper (I) iodide (CuI) (93 mg, 0.49 mmol), and tetrakis(triphenylphosphine)palladium(0) ($Pd(PPh_3)_4$) (0.28 g, 0.24 mmol) in a degassed toluene/diisopropylamine (DIPA) mixture (4/1, v/v; 90 mL) was added trimethylsilylacetylene (TMSA) (9.5 mL, 68 mmol). After stirring at 40 °C for 14 h, the mixture was diluted with *n*-hexane and the solution was washed with aqueous 1 N HCl, saturated aqueous $NaHCO_3$, and water, and then dried over Na_2SO_4. The solvent was removed under reduced pressure and the crude product was purified by silica gel chromatography using *n*-hexane as the eluent to give the desired product as a pale yellow oil (14.4 g, 96% yield). ^{1}H NMR (500 MHz, $CDCl_3$, 25 °C): δ 6.55 (s, 2H, Ar–H), 4.33 (sex, J = 6.1 Hz, 1H, OCH), 2.39 (s, 6H, CH_3), 1.73-1.50 (m, 2H, CH_2), 1.44-1.25 (m, 11H, CH_2, CH_3), 0.88 (t, J = 7.1 Hz, 3H, CH_3), 0.25 (s, 9H, TMS). ^{13}C NMR (126 MHz, $CDCl_3$, 25 °C): δ 157.72, 142.36, 115.06, 114.06, 103.11, 100.61, 73.57, 36.45, 31.79, 29.26, 25.46, 22.60, 21.27, 19.75, 14.08, 0.27.

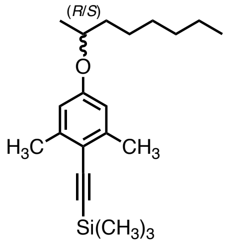

rac-**5**

Synthesis of *rac*-6. Potassium carbonate (26.5 g, 192 mmol) and *rac*-**5** (12.0 g, 36.2 mmol) were dissolved in methanol (100 mL) and the mixture was stirred at room temperature for 24 h. After evaporating the solvent, the residue was diluted with *n*-hexane and the solution was washed with water, and then dried over Na_2SO_4. The solvent was removed under reduced pressure and the crude product was purified by silica gel chromatography using *n*-hexane as the eluent to give the desired product as a brown oil (6.62 g, 71% yield). IR (ATR, cm^{-1}): 3313 (≡CH), 2097 (C≡C). ^{1}H NMR (500 MHz, $CDCl_3$, 25 °C): δ 6.57 (s, 2H, Ar–H), 4.34 (sex, J = 6.0 Hz, 1H, OCH), 3.40 (s, 1H, C≡CH), 2.41 (s, 6H, CH_3), 1.74-1.51 (m, 2H, CH_2), 1.46-1.26 (m, 11H, CH_2, CH_3), 0.88 (t, J = 7.0 Hz, 3H, CH_3). ^{13}C NMR (126 MHz, $CDCl_3$, 25 °C): δ 157.89, 142.65,

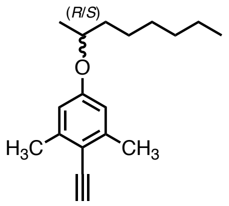

rac-**6**

114.06, 113.93, 83.48, 81.44, 73.57, 36.47, 31.79, 29.26, 25.49, 22.60, 21.25, 19.75, 14.08. HRMS (EI+): *m*/*z* calcd for $C_{18}H_{26}O$ (M^+), 258.1978; found 258.1975.

rac-**6** was resolved into enantiomers ((*R*)-**6** and (*S*)-**6**) (≥99% ee) by chiral HPLC (Figure S1a–c). Chiral HPLC conditions: column, CHIRALPAK IB (2.0 cm (i.d.) × 20 cm); eluent, *n*-hexane; flow rate, 8.0 mL/min; temperature, room temperature. The absolute configurations were assigned based on the retention time of (*R*)-rich **6** synthesized through a three-step reaction, which started with the etherification of 4-iodo-3,5-dimethylphenol with the optically-active (*S*)-*p*-toluenesulfonic acid 2-octyl ester[S1] involving stereochemical inversion and partial racemization (see below and Scheme S2 and Figure S1d).

Analytical data of (*R*)-**6**: Brown oil. $[\alpha]^{25}_D$ –10.0 (*c* 0.2, $CHCl_3$). IR (ATR, cm^{-1}): 3313 (≡CH), 2097 (C≡C). ^{1}H NMR (500 MHz, $CDCl_3$, 25 °C): δ 6.57 (s, 2H, Ar–H), 4.34 (sex, J = 6.1 Hz, 1H, OCH), 3.40 (s, 1H, C≡CH), 2.41 (s, 6H, CH_3), 1.74-1.51 (m, 2H, CH_2), 1.46-1.26 (m, 11H, CH_2, CH_3), 0.88 (t, J = 7.0 Hz, 3H, CH_3). ^{13}C NMR (126 MHz, $CDCl_3$, 25 °C): δ 157.87, 142.63, 114.05, 113.91, 83.48, 81.43, 73.55, 36.46, 31.78, 29.25, 25.48, 22.60, 21.25, 19.75, 14.08. HRMS (EI+): *m*/*z* calcd for $C_{18}H_{26}O$ (M^+), 258.1978; found 258.1986.

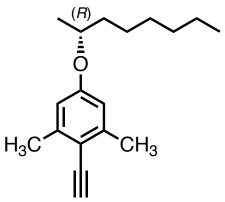

(*R*)-**6**

Analytical data of (*S*)-**6**: Brown oil. $[\alpha]^{25}_D$ +9.3 (*c* 0.2, $CHCl_3$). IR (ATR, cm^{-1}): 3313 (≡CH), 2097 (C≡C). ^{1}H NMR (500 MHz, $CDCl_3$, 25 °C): δ 6.57 (s, 2H, Ar–H), 4.34 (sex, J = 6.1 Hz, 1H, OCH), 3.40 (s, 1H, C≡CH), 2.41 (s, 6H, CH_3), 1.74-1.51 (m, 2H, CH_2), 1.46-1.26 (m, 11H, CH_2, CH_3), 0.88 (t, J = 6.7 Hz, 3H, CH_3). ^{13}C NMR (126 MHz, $CDCl_3$, 25 °C): δ 157.89, 142.65, 114.06, 113.93, 83.48, 81.44, 73.57, 36.47, 31.79, 29.26, 25.49, 22.60, 21.25, 19.75, 14.08. HRMS (EI+): *m*/*z* calcd for $C_{18}H_{26}O$ (M^+), 258.1978; found 258.1981.

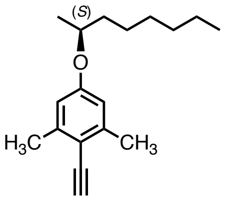

(*S*)-**6**

Synthesis of (*R*,*R*)-1a. To a mixture of 1,4-dibromo-2,5-diiodobenzene (0.90 g, 1.8 mmol), CuI (41 mg, 0.22 mmol), and $Pd(PPh_3)_4$ (97 mg, 0.084 mmol) in a degassed toluene/DIPA mixture (4/1, v/v; 20 mL) was added (*R*)-**6** (1.05 g, 4.06 mmol). After stirring at 50 °C for 14 h, the mixture was diluted with chloroform and the solution was washed with water, and then dried over Na_2SO_4. The solvent was removed under reduced pressure and the residue was passed through a short pad of silica gel using chloroform as the eluent. After concentrating in vacuo, the crude product was purified by recycling preparative HPLC on JAIGEL-1HR and JAIGEL-2HR (60 cm × 2.0 cm (i.d.)) using chloroform as the eluent to give the desired product as a yellow solid (0.72 g, 52% yield). Mp: 134.4–135.1 °C. $[\alpha]^{25}_D$ +12.3 (*c* 0.2,

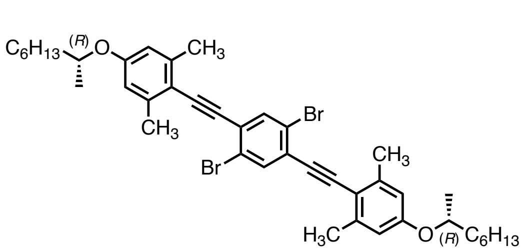

(*R*,*R*)-**1a**

$CHCl_3$). IR (ATR, cm^{-1}): 2207 (C≡C). ^1H NMR (500 MHz, $CDCl_3$, 25 °C): δ 7.74 (s, 2H, Ar–H), 6.61 (s, 4H, Ar–H), 4.38 (sex, J = 6.1 Hz, 2H, OCH), 2.52 (s, 12H, CH_3), 1.75-1.53 (m, 4H, CH_2), 1.45-1.29 (m, 22H, CH_2, CH_3), 0.88 (t, J = 7.0 Hz, 6H, CH_3). ^{13}C NMR (126 MHz, $CDCl_3$, 25 °C): δ 158.52, 142.82, 135.66, 126.62, 122.88, 114.23, 114.13, 95.04, 93.71, 73.66, 36.47, 31.79, 29.26, 25.48, 22.61, 21.60, 19.78, 14.08. HRMS (APCI+): m/z calcd for $C_{42}H_{53}Br_2O_2$ ($M+H^+$), 747.2407; found 747.2404.

Synthesis of (*S*,*S*)-1a. To a mixture of 1,4-dibromo-2,5-diiodobenzene (0.44 g, 0.91 mmol), CuI (20 mg, 0.11 mmol), and $Pd(PPh_3)_4$ (45 mg, 0.039 mmol) in a degassed toluene/DIPA mixture (4/1, v/v; 5 mL) was added (*S*)-**6** (0.53 g, 2.1 mmol). After stirring at 50 °C for 14 h, the mixture was diluted with chloroform and the solution was washed with water, and then dried over Na_2SO_4. The solvent was removed under reduced pressure and the residue was passed through a short pad of silica gel using chloroform as the eluent. After concentrating in vacuo, the crude product was purified by recycling preparative HPLC on JAIGEL-1HR and JAIGEL-2HR (60 cm × 2.0 cm (i.d.)) using chloroform as the eluent to give the desired product as a yellow solid (0.38 g, 56% yield). Mp: 134.4–135.2 °C. $[\alpha]^{25}_D$ –13.1 (c 0.2, $CHCl_3$). IR (ATR, cm^{-1}): 2207 (C≡C). ^1H NMR (500 MHz, $CDCl_3$, 25 °C): δ 7.73 (s, 2H, Ar–H), 6.61 (s, 4H, Ar–H), 4.38 (sex, J = 6.1 Hz, 2H, OCH), 2.52 (s, 12H, CH_3), 1.76-1.53 (m, 4H, CH_2), 1.48-1.29 (m, 22H, CH_2, CH_3), 0.88 (t, J = 7.0 Hz, 6H, CH_3). ^{13}C NMR (126 MHz, $CDCl_3$, 25 °C): δ 158.50, 142.81, 135.64, 126.60, 122.87, 114.21, 114.10, 95.03, 93.71, 73.64, 36.46, 31.79, 29.25, 25.48, 22.61, 21.60, 19.77, 14.09. HRMS (APCI+): m/z calcd for $C_{42}H_{53}Br_2O_2$ ($M+H^+$), 747.2407; found 747.2416.

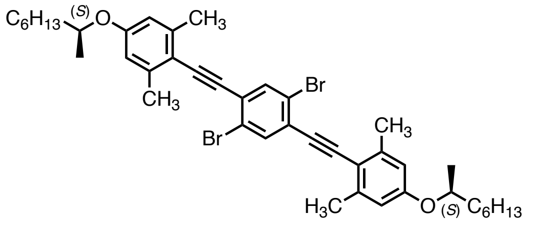

(*S*,*S*)-**1a**

Nonracemic **6** ((*R*)-rich **6**) was synthesized according to Scheme S2 to confirm the absolute configuration of optically-active **6** described above (Figure S1).

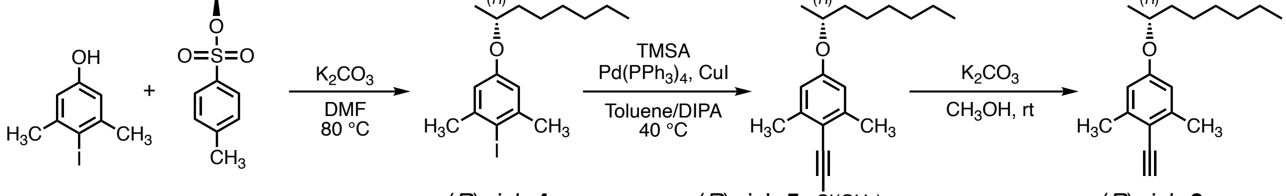

(*R*)-rich **4** (*R*)-rich **5** (*R*)-rich **6**

Scheme S2. Synthesis of (*R*)-rich **6**.

Synthesis of (*R*)-rich 4. To a mixture of 4-iodo-3,5-dimethylphenol (0.62 g, 2.5 mmol) and potassium carbonate (0.92 g, 6.6 mmol) in anhydrous DMF (10 mL) was added (*S*)-*p*-toluenesulfonic acid 2-octyl ester (0.86 g, 3.0 mmol). After stirring at 80 °C for 14 h, the mixture was cooled to room temperature and diluted with ethyl acetate/*n*-hexane (4/1, v/v). The solution was washed with water, and then dried over Na_2SO_4. The solvents were removed under reduced pressure and the crude product was purified by silica gel chromatography using *n*-hexane as the eluent to give the desired product as a colorless oil (0.41 g, 46% yield). ^{1}H NMR (500 MHz, $CDCl_3$, 25 °C): δ 6.65 (s, 2H, Ar–H), 4.30 (sex, J = 5.9 Hz, 1H, OCH), 2.43 (s, 6H, CH_3), 1.73-1.50 (m, 2H, CH_2), 1.46-1.26 (m, 11H, CH_2, CH_3), 0.88 (t, J = 7.0 Hz, 3H, CH_3).

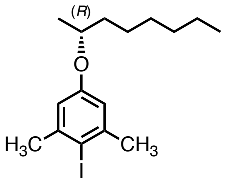

(*R*)-rich **4**

This reaction proceeded with partial racemization, resulting in (*R*)-rich **4**, as confirmed by the chiral HPLC analysis of the subsequent reaction product ((*R*)-rich **6**) (see Figure S1d).

Synthesis of (*R*)-rich 5. To a mixture of (*R*)-rich **4** (0.41 g, 1.2 mmol), CuI (2.9 mg, 0.015 mmol), and $Pd(PPh_3)_4$ (8.3 mg, 7.2 μmol) in a degassed toluene/DIPA mixture (4/1, v/v; 5 mL) was added TMSA (0.25 mL, 1.8 mmol). After stirring at 40 °C for 17 h, the mixture was diluted with *n*-hexane and the solution was washed with aqueous 1 N HCl, saturated aqueous $NaHCO_3$, and water, and then dried over Na_2SO_4. The solvent was removed under reduced pressure and the crude product was purified by silica gel chromatography using *n*-hexane as the eluent to give the desired product as a pale yellow oil (0.36 g, 95% yield). ^{1}H NMR (500 MHz, $CDCl_3$, 25 °C): δ 6.55 (s, 2H, Ar–H), 4.33 (sex, J = 6.1 Hz, 1H, OCH), 2.39 (s, 6H, CH_3), 1.73-1.50 (m, 2H, CH_2), 1.44-1.25 (m, 11H, CH_2, CH_3), 0.88 (t, J = 7.0 Hz, 3H, CH_3), 0.25 (s, 9H, TMS).

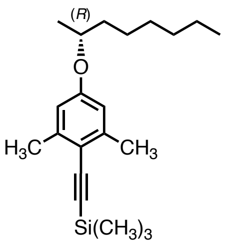

(*R*)-rich **5**

Synthesis of (*R*)-rich 6. Potassium carbonate (0.79 g, 5.7 mmol) and (*R*)-rich **5** (0.36 g, 1.1 mmol) were dissolved in methanol (5 mL) and the mixture was stirred at room temperature for 3 h. After evaporating the solvent, the residue was diluted with *n*-hexane and the solution was washed with water, and then dried over Na_2SO_4. The solvent was removed under reduced pressure and the crude product was purified by silica gel chromatography using *n*-hexane as the eluent to give the desired product as a brown oil (0.21 g, 77% yield). ^{1}H NMR (500 MHz, $CDCl_3$, 25 °C): δ 6.57 (s, 2H, Ar–H), 4.34 (sex, J = 6.1 Hz, 1H, OCH), 3.40 (s, 1H, C≡CH), 2.41 (s, 6H, CH_3), 1.74-1.50 (m, 2H, CH_2), 1.45-1.27 (m, 11H, CH_2, CH_3), 0.88 (t, J = 7.0 Hz, 3H, CH_3).

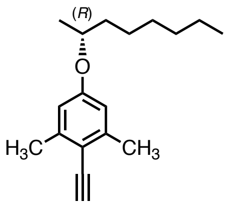

(*R*)-rich **6**

Polymerization. Poly-(*R*)-**2a**, poly-(*S*)-**2a**, and poly-CA^{Pre} were prepared by Suzuki−Miyaura coupling copolymerizations of 2,7-bis(4,4,5,5-tetramethyl-1,3,2-dioxaborolan-2-yl)-9,9-di-*n*-octylfluorene (**FL$_{Bpin}$**) and 9-(9-heptadecanyl)-2,7-bis(4,4,5,5-tetramethyl-1,3,2-dioxaborolan-2-yl)carbazole (**CA$_{Bpin}$**) with (*R*,*R*)-**1a**, (*S*,*S*)-**1a**, or **1c** in a dry Schlenk flask under a dry nitrogen atmosphere (Scheme S3). The results of the copolymerizations are summarized in Table S1. A typical procedure for the copolymerization of **FL** with (*R*,*R*)-**1a** is described as follows.

FL$_{Bpin}$ (96.4 mg, 0.150 mmol), (*R*,*R*)-**1a** (109 mg, 0.146 mmol), and tripotassium phosphate (193 mg, 0.909 mmol) were placed in a dry Schlenk flask, which was then evacuated on a vacuum line and flushed with dry nitrogen. After this evacuation-flush procedure was repeated three times, a degassed tetrahydrofuran (THF)/water mixture (2/1, v/v; 3.0 mL) was added using a syringe. To this was added P(*t*-Bu)$_3$ Pd G2 (9.3 mg, 18 μmol) and the mixture was stirred at room temperature for 2 h, and then diluted with chloroform. The solution was washed with water, and then dried over Na_2SO_4. After filtration, most of the solvent was removed under reduced pressure and the concentrated solution was poured into a large amount of methanol. The resulting polymer was collected by centrifugation, washed with methanol, and dried in vacuo at room temperature (123 mg, 86% yield) (entry 1 in Table S1). In the same way, poly-(*S*)-**2a** and poly-CA^{Pre} were prepared (entries 2 and 3). The M_n and M_w/M_n values of the polymers were estimated by SEC using polystyrene standards in chloroform.

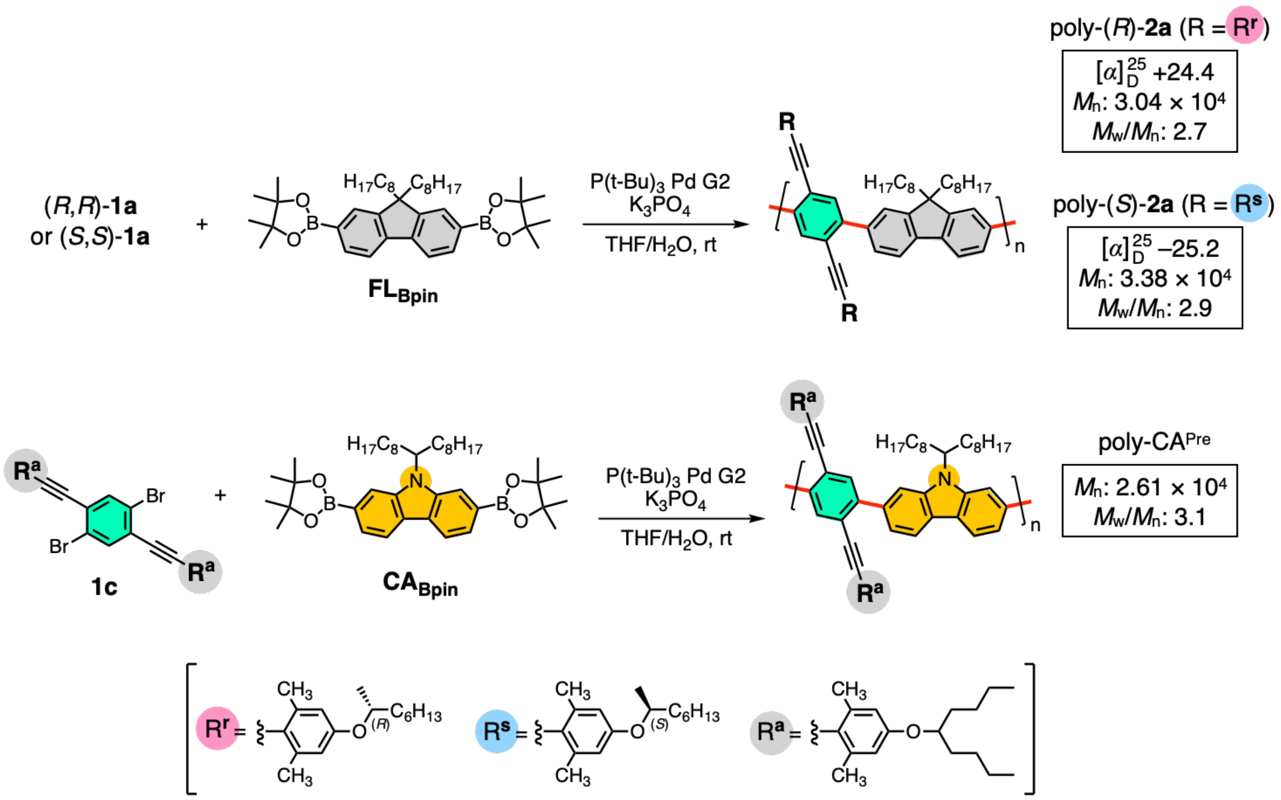

Scheme S3. Synthesis of poly-(*R*)-**2a**, poly-(*S*)-**2a**, and poly-CA^{Pre}.

Table S1. Copolymerization results of **FL_{Bpin}** with (R,R)- and (S,S)-**1a** and those of **CA_{Bpin}** with **1c** using P(t-Bu)$_3$ Pd G2 in the presence of K_3PO_4 in THF/H_2O (2/1, v/v) at room temperature for 2 h.[a]

Entry	Monomer in feed (mol%)		Copolymer				
			Sample code	Yield (%)	M_n (10^4)^b	M_w/M_n ^{b}	DP$_n$ ^{c}
1	**FL_{Bpin}** (50.5)	(R,R)-**1a** (49.5)	poly-(R)-**2a**	86	3.04	2.7	31
2	**FL_{Bpin}** (50.5)	(S,S)-**1a** (49.5)	poly-(S)-**2a**	66	3.38	2.9	35
3	**CA_{Bpin}** (50.5)	**1c** (49.5)	poly-CA^{Pre}	47	2.61	3.1	26

[a] [**FL_{Bpin}** or **CA_{Bpin}**] = 0.050 M, [**1a** or **1c**] = 0.049 M, [P(t-Bu)$_3$ Pd G2] = 6.0 mM, [P(t-Bu)$_3$ Pd G2]/[K_3PO_4] = 1/52.

[b] Estimated by SEC (polystyrene standards) with chloroform as the eluent.

[c] Number-average degree of polymerization estimated by M_n.

Analytical data of poly-(R)-**2a**: Pale yellow solid. $[\alpha]^{25}_D$ +24.4 (c 0.2, $CHCl_3$). IR (ATR, cm^{-1}): 2201 (C≡C). ^{1}H NMR (500 MHz, $CDCl_3$, 50 °C): δ 7.85–7.65 (br, 6H, Ar–H), 7.65–7.55 (br, 2H, Ar–H), 6.55–6.45 (br, 4H, Ar–H), 4.35-4.25 (br, 2H, OCH), 2.30-2.15 (br, 12H, CH_3), 2.10-1.80 (br, 4H, CH_2), 1.75-1.48 (br, 4H, CH_2), 1.43-0.70 (br, 58H, CH_2, CH_3).

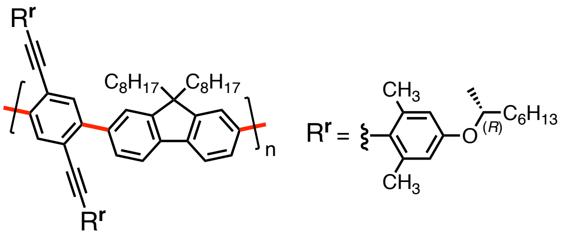

poly-(R)-**2a**

Analytical data of poly-(S)-**2a**: Pale yellow solid. $[\alpha]^{25}_D$ –25.2 (c 0.2, $CHCl_3$). IR (ATR, cm^{-1}): 2202 (C≡C). ^{1}H NMR (500 MHz, $CDCl_3$, 50 °C): δ 7.90–7.65 (br, 6H, Ar–H), 7.65–7.55 (br, 2H, Ar–H), 6.55–6.45 (br, 4H, Ar–H), 4.35-4.25 (br, 2H, OCH), 2.30-2.15 (br, 12H, CH_3), 2.15-1.80 (br, 4H, CH_2), 1.75-1.48 (br, 4H, CH_2), 1.43-0.70 (br, 58H, CH_2, CH_3).

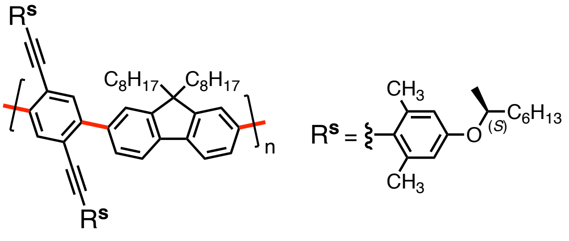

poly-(S)-**2a**

Analytical data of poly-CA^{Pre}: Pale yellow solid. IR (ATR, cm^{-1}): 2201 (C≡C). ^{1}H NMR (500 MHz, $CDCl_3$, 50 °C): δ 8.35–8.00 (br, 2H, Ar–H), 8.00–7.40 (br, 6H, Ar–H), 6.60–6.35 (br, 4H, Ar–H), 4.75-4.45 (br, 1H, NCH), 4.30-4.00 (br, 2H, OCH), 2.40-1.80 (br, 16H, CH_2, CH_3), 1.75-1.50 (br, 8H, CH_2), 1.40-0.95 (br, 40H, CH_2), 0.95-0.65 (br, 18H, CH_3).

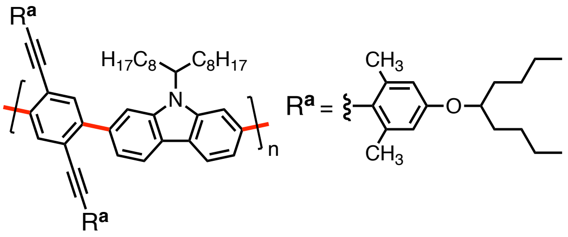

poly-CA^{Pre}

Alkyne Benzannulations of Precursor Polymers. Poly-(*R*)-**3a**, poly-(*S*)-**3a**, and poly-CA were prepared by alkyne benzannulations of the corresponding precursor polymers (poly-(*R*)-**2a**, poly-(*S*)-**2a**, and poly-CA^{Pre}, respectively) with trifluoroacetic acid (TFA) in a dry Schlenk flask under a dry nitrogen atmosphere (Scheme S4). A typical procedure for the modified alkyne benzannulation of poly-(*R*)-**2a** is described as follows.

The precursor polymer poly-(*R*)-**2a** (52 mg, 53 μmol) was placed in a dry Schlenk flask, which was then evacuated on a vacuum line and flushed with dry nitrogen. After this evacuation-flush procedure was repeated three times, an anhydrous dichloromethane/TFA mixture (60/1, v/v; 5.0 mL) was added using a syringe. After stirring at room temperature for 17 h, the reaction mixture was diluted with chloroform and the solution was washed with saturated aqueous $NaHCO_3$ and water, and then dried over Na_2SO_4. After filtration, most of the solvents were removed under reduced pressure and the concentrated solution was poured into a large amount of methanol. The resulting polymer was collected by centrifugation, washed with methanol, and dried in vacuo to yield poly-(*R*)-**3a** as a yellow solid (51 mg, >99% yield). In the same way, the alkyne benzannulations of poly-(*S*)-**2a** and poly-CA^{Pre} were carried out to prepare poly-(*S*)-**3a** and poly-CA, respectively. The M_n and M_w/M_n values of the ladder polymers were estimated by SEC using polystyrene standards in chloroform. The M_n and M_w/M_n values of poly-CA could not be determined by SEC due to unfavorable interactions with the packing material in the SEC column (Tosoh TSKgel GMH_{HR}-M).

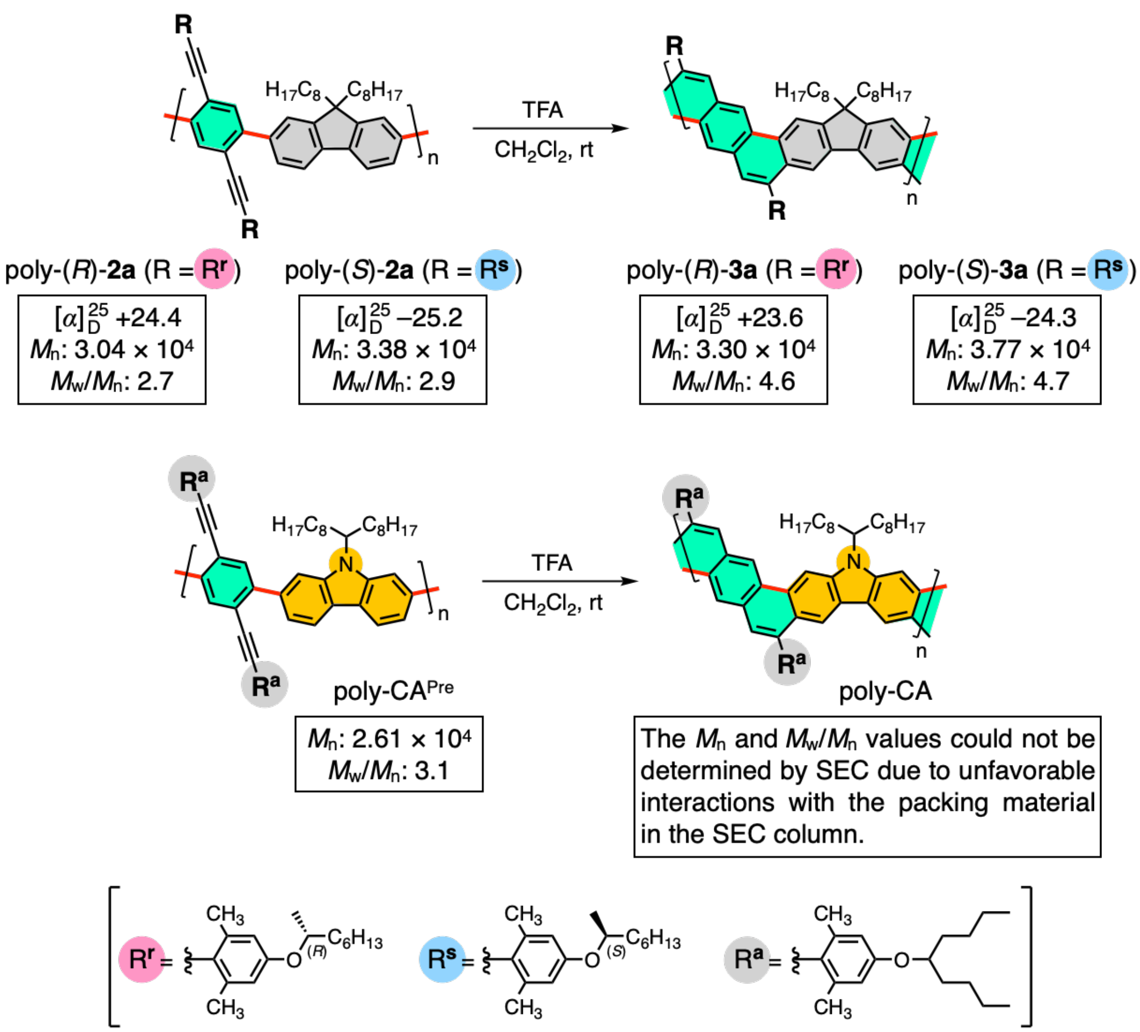

Scheme S4. Synthesis of poly-(*R*)-**3a**, poly-(*S*)-**3a**, and poly-CA.

Analytical data of poly-(*R*)-**3a**: Yellow solid. Yield: >99%. $[\alpha]^{25}_{D}$ +23.6 (*c* 0.2, $CHCl_3$). ^{1}H NMR (500 MHz, $CDCl_3$, 50 °C): δ 9.50–9.05 (br, 2H, Ar–H), 9.05–8.50 (br, 2H, Ar–H), 8.00–7.50 (br, 4H, Ar–H), 7.00–6.60 (br, 4H, Ar–H), 4.70-4.30 (br, 2H, OCH), 2.70-1.80 (br, 20H, CH_2, CH_3), 1.80-0.55 (br, 58H, CH_2, CH_3).

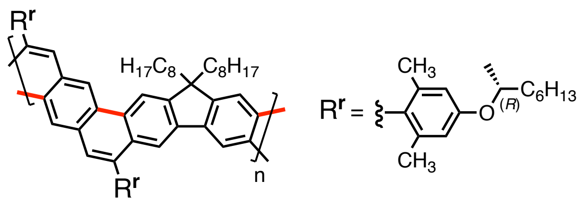

poly-(*R*)-**3a**

Analytical data of poly-(*S*)-**3a**: Yellow solid. Yield: >99%. $[\alpha]^{25}_{D}$ –24.3 (*c* 0.2, $CHCl_3$). ^{1}H NMR (500 MHz, $CDCl_3$, 50 °C): δ 9.60–9.05 (br, 2H, Ar–H), 9.05–8.40 (br, 2H, Ar–H), 8.00–7.50 (br, 4H, Ar–H), 7.00–6.50 (br, 4H, Ar–H), 4.70-4.30 (br, 2H, OCH), 2.70-1.80 (br, 20H, CH_2, CH_3), 1.80-0.55 (br, 58H, CH_2, CH_3).

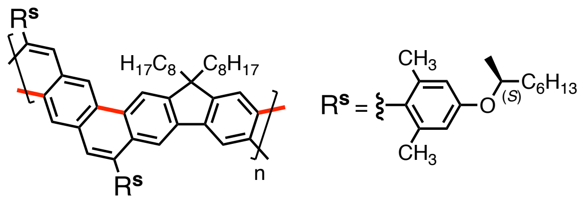

poly-(*S*)-**3a**

Analytical data of poly-CA: Yellow solid. Yield: >99%. ^{1}H NMR (500 MHz, $CDCl_3$, 50 °C): δ 9.70–9.60 (br, 4H, Ar–H), 8.50–8.00 (br, 2H, Ar–H), 7.90–7.60 (br, 2H, Ar–H), 7.00–6.50 (br, 4H, Ar–H), 5.40-4.80 (br, 1H, NCH), 4.60-4.10 (br, 2H, OCH), 3.00-2.50 (br, 2H, CH_2), 2.50-1.65 (br, 22H, CH_2, CH_3), 1.65-0.50 (br, 58H, CH_2, CH_3).

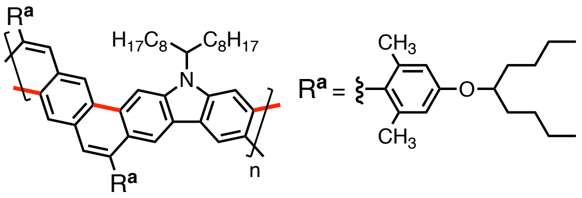

poly-CA

3. Molecular Modeling of Packing Structures of Poly-(*R*)-3a

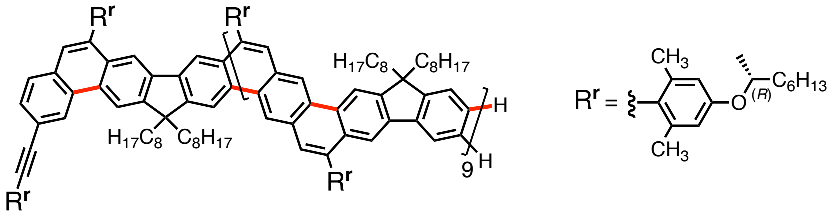

poly-(*R*)-**3a** (9 repeating monomer units)

The molecular modeling and molecular mechanics (MM) calculations were conducted using the Compass II force field[S4] as implemented in the Materials Studio modeling software (Version 8.0; Dassault Systèmes BIOVIA, San Diego, CA, USA) operated using a PC running under Windows 10. First, the polymer model of poly-(*R*)-**3a** (9 repeating monomer units) was constructed by a Polymer Builder module in the Materials Studio modeling software. The face-to-face and edge-to-edge packing structures of the four poly-(*R*)-**3a** chains were generated by manually positioning them as close as possible so that their backbone π-planes oriented face-to-face (Figure S25a) and edge-to-edge (Figure S25b), respectively. The relative positions of the poly-(*R*)-**3a** chains were determined in order to avoid any unfavorable van der Waals contacts. The poly-(*R*)-**3a** packing structures were then geometry-optimized by the MM calculations (Compass II force field) (Figure S25).

4. Supporting Data

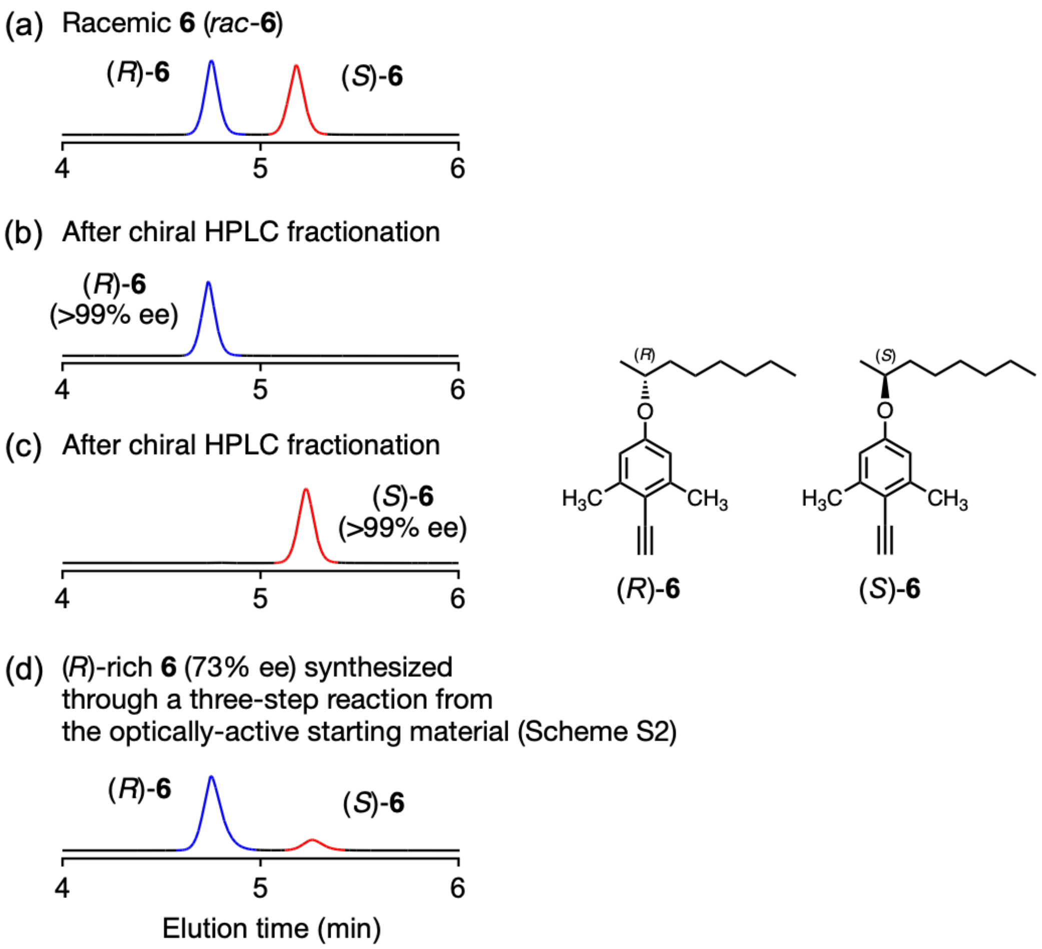

Figure S1. Chromatograms for the resolution of *rac*-**6** (a), the fractionated (*R*)-**6** (b) and (*S*)-**6** (c), and (*R*)-rich **6** synthesized through a three-step reaction from the optically-active starting material ((*S*)-*p*-toluenesulfonic acid 2-octyl ester), which was accompanied by stereochemical inversion and partial racemization (d). Chromatographic conditions: column, CHIRALPAK IB (0.46 cm (i.d.) × 25 cm); eluent, *n*-hexane; flow rate, 1.0 mL/min; temperature, 0 °C.

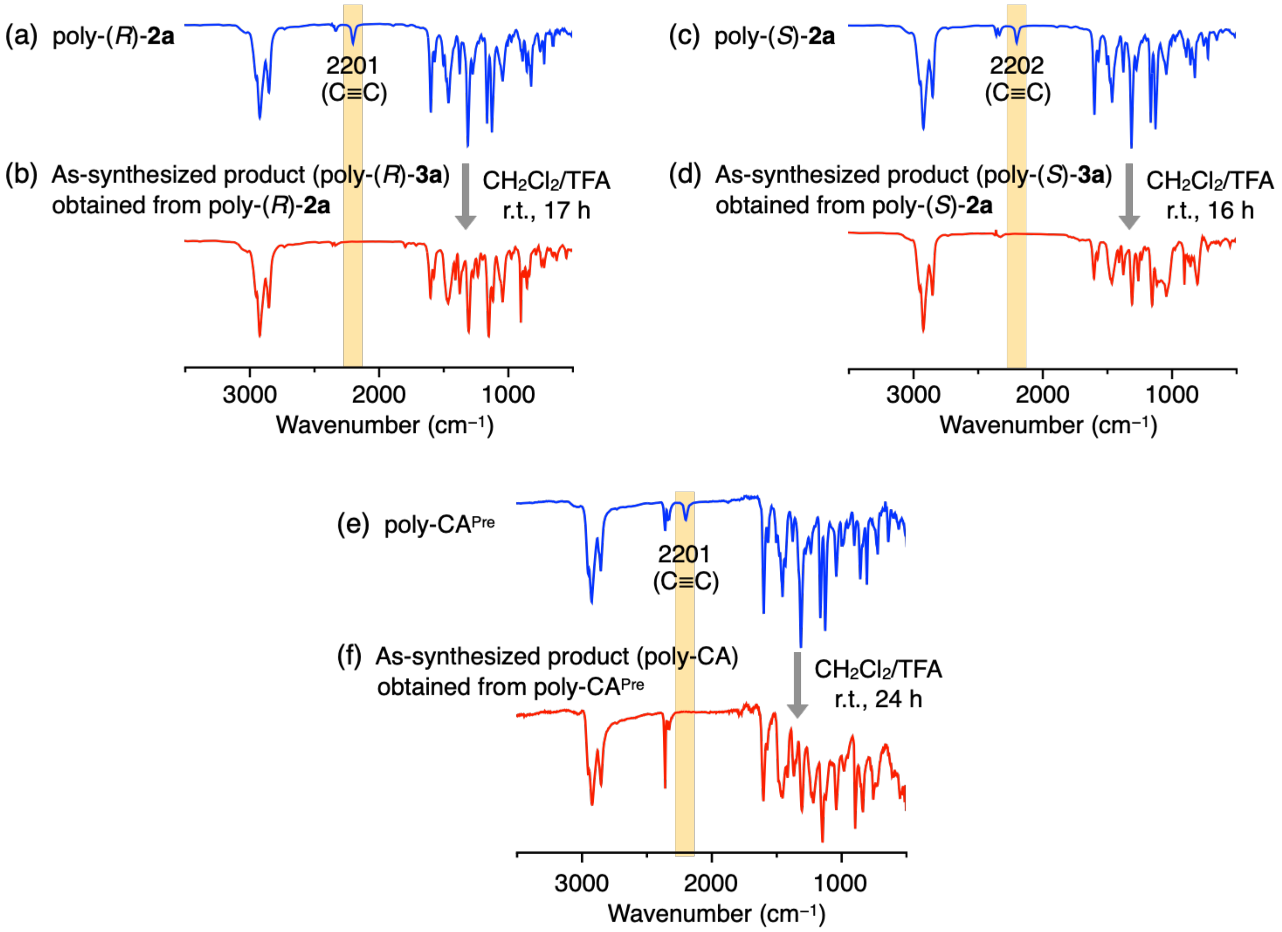

Figure S2. IR spectra of poly-(*R*)-**2a** (a), poly-(*S*)-**2a** (c), poly-CA^{Pre} (e), and those of the as-synthesized products after acid-promoted cyclizations in a dichloromethane/TFA (60/1, v/v) mixture at room temperature, measured in ATR mode at room temperature.

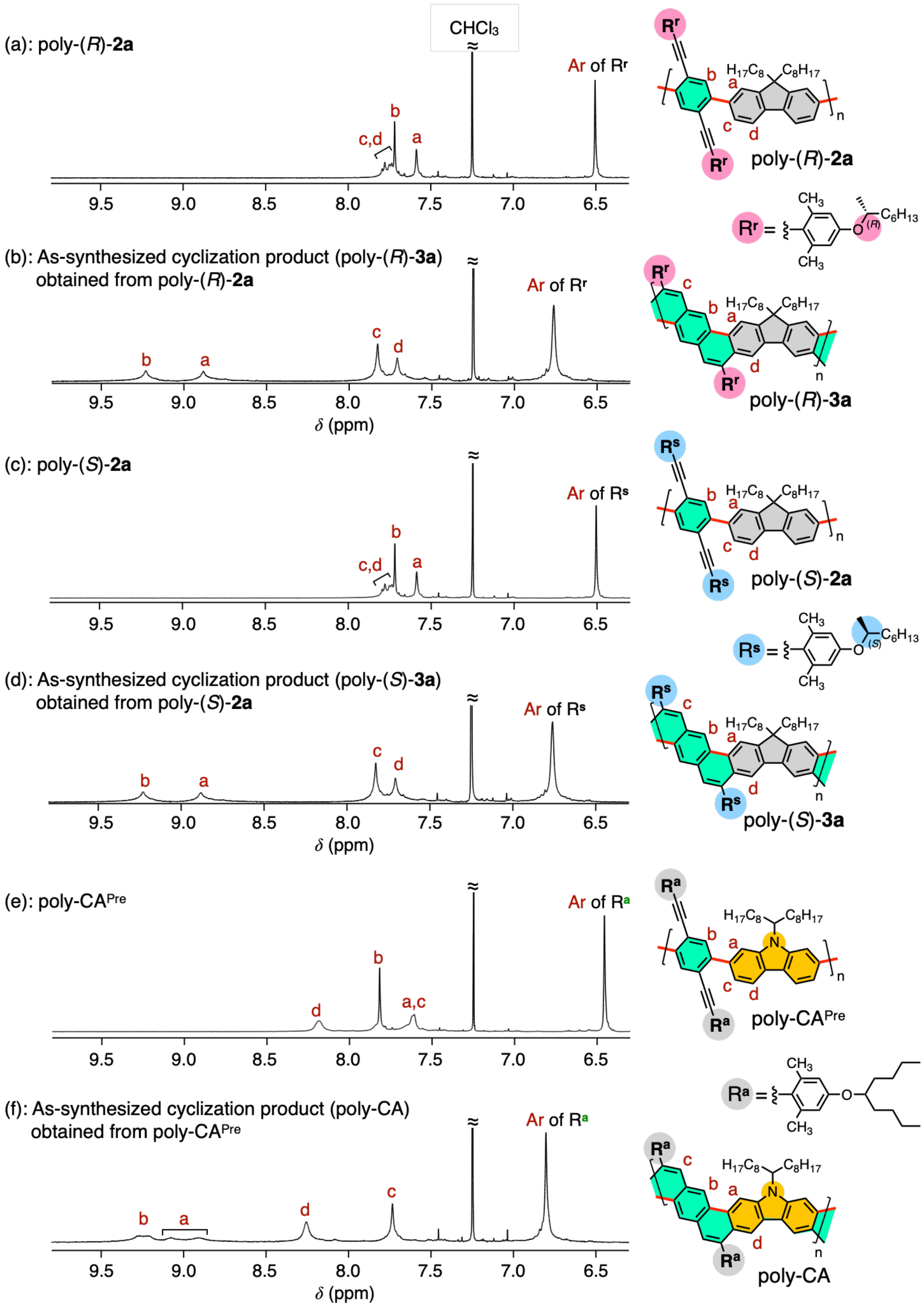

Figure S3. ^{1}H NMR spectra (500 MHz, $CDCl_3$, 50 °C) of poly-(*R*)-**2a** (a), poly-(*S*)-**2a** (c), poly-CA^{Pre} (e), and as-synthesized cyclization products (poly-(*R*)-**3a** (b), poly-(*S*)-**3a** (d), and poly-CA (f)) obtained from poly-(*R*)-**2a**, poly-(*S*)-**2a**, and poly-CA^{Pre}, respectively. For the signal assignments, see ref [S3] for (a – d) and Figures S4 and S5 for (e) and (f), respectively.

Splitting of the aromatic protons (Ha and Hb) of poly-CA was probably due to atropisomers of the α-branched alkyl chain generated by its hindered rotation.[S5]

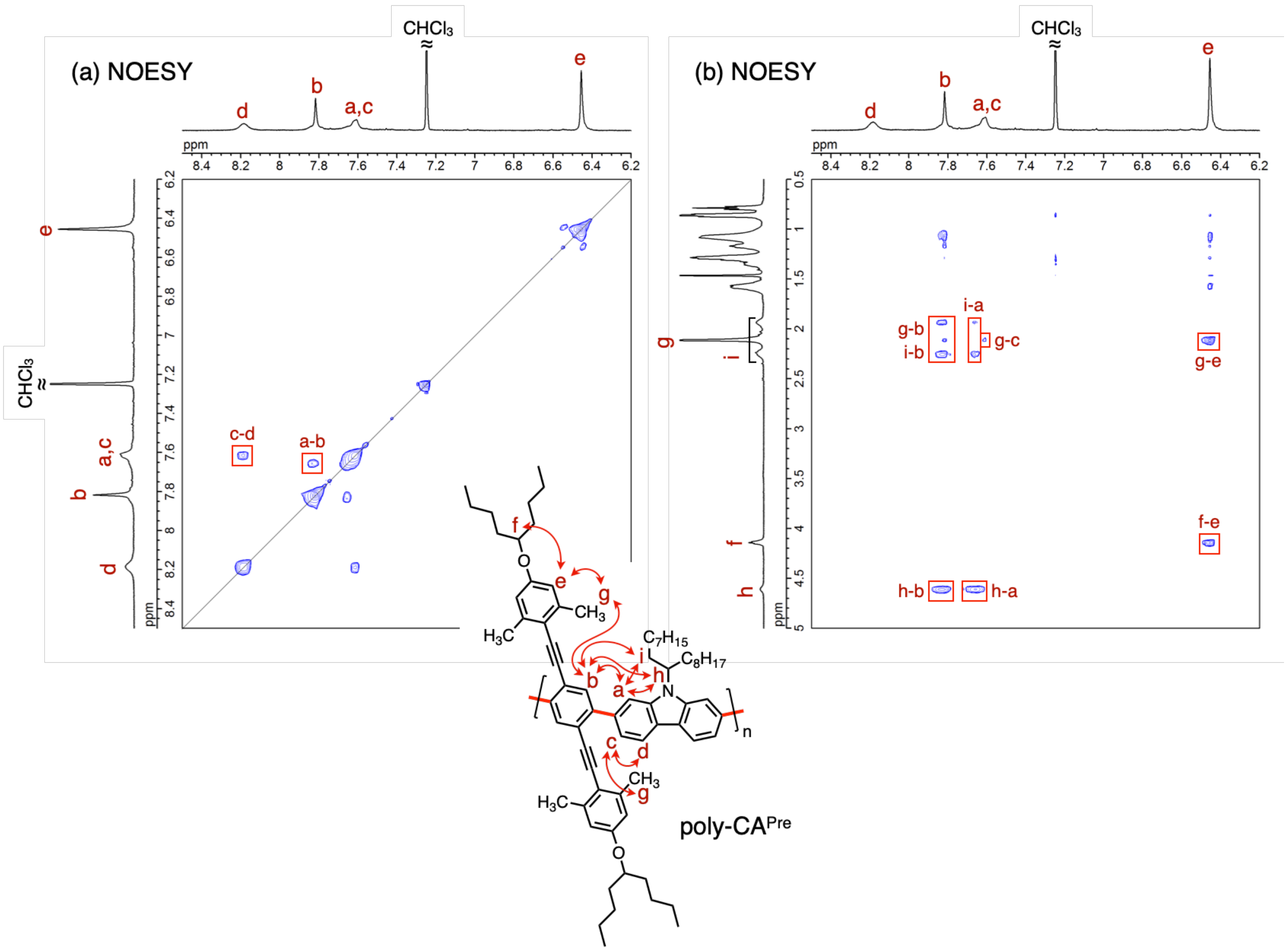

Figure S4. Partial NOESY spectra (500 MHz, $CDCl_3$, 50 °C, mixing time = 500 ms (NOESY)) of poly-CA^{Pre}.

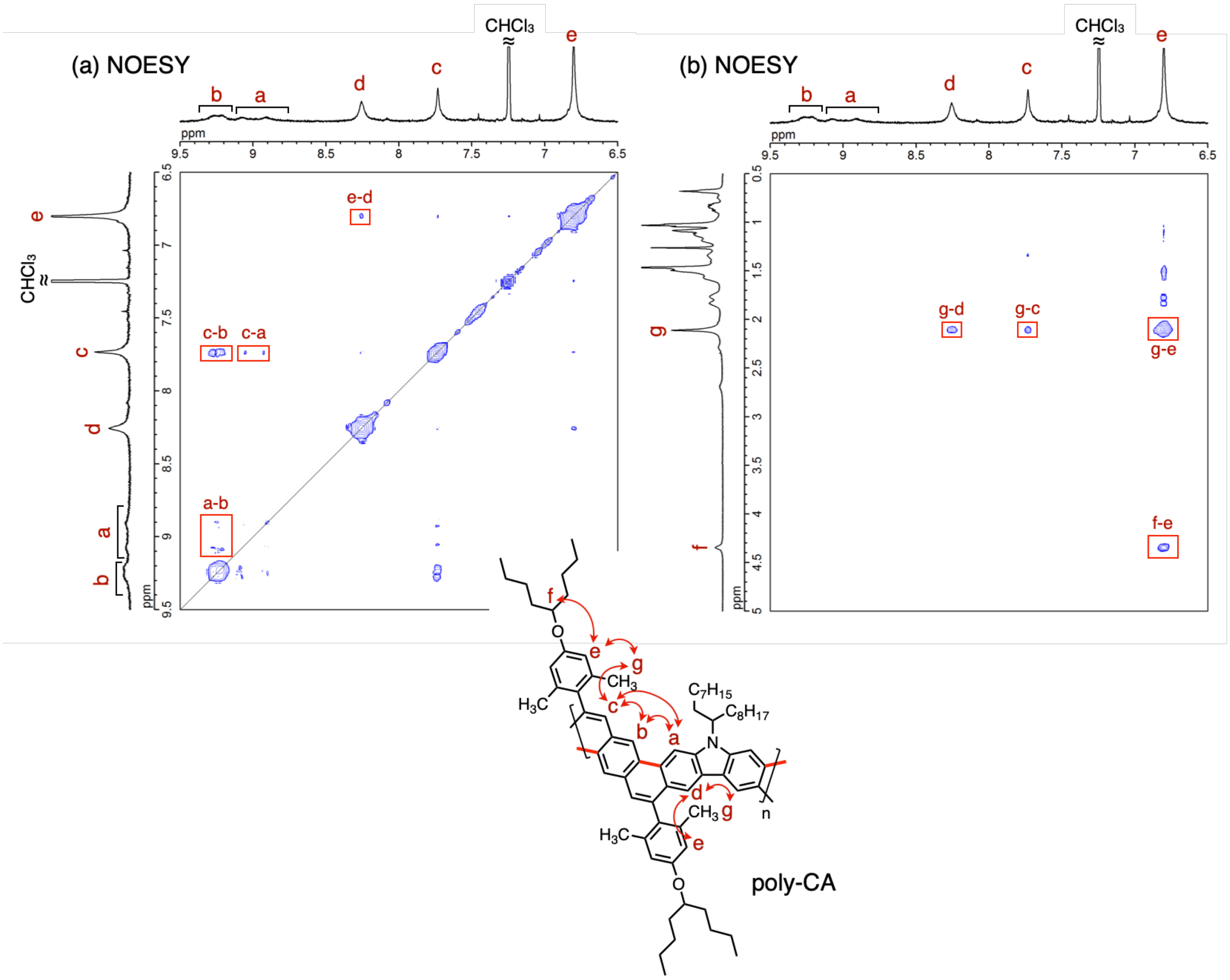

Figure S5. Partial NOESY spectra (500 MHz, $CDCl_3$, 50 °C, mixing time = 500 ms (NOESY)) of poly-CA.

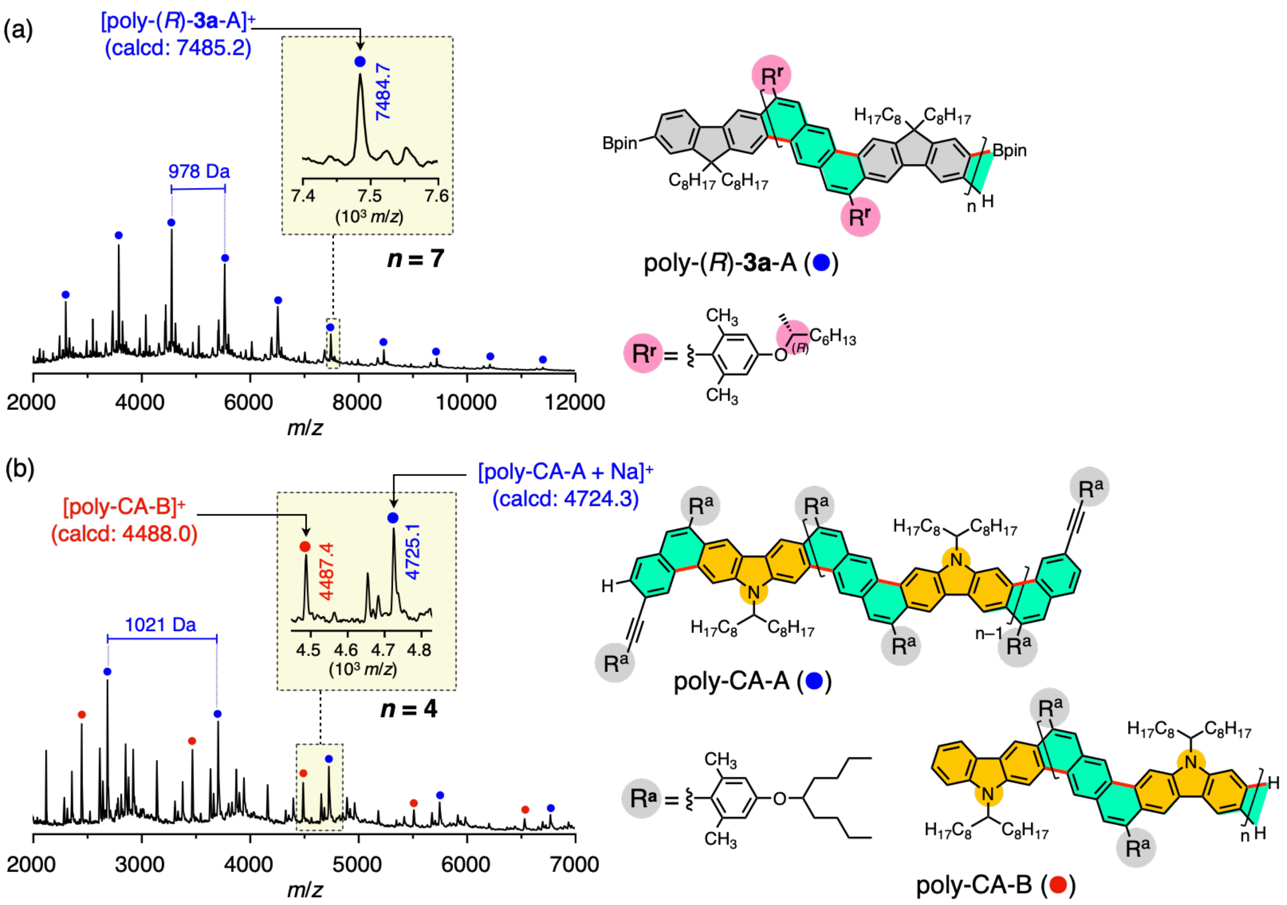

Figure S6. MALDI-TOF-MS spectra of poly-(*R*)-**3a** (a) and poly-CA (b). The MS measurements were performed using *trans*-2-[3-(4-*tert*-butylphenyl)-2-methyl-2-propenylidene]malononitrile as a matrix.

The MALDI-TOF-MS spectra showed main series of peaks with regular intervals of approximately 978 (a) and 1021 (b) (*m*/*z*) mass that correspond to the molar masses of the repeating units.

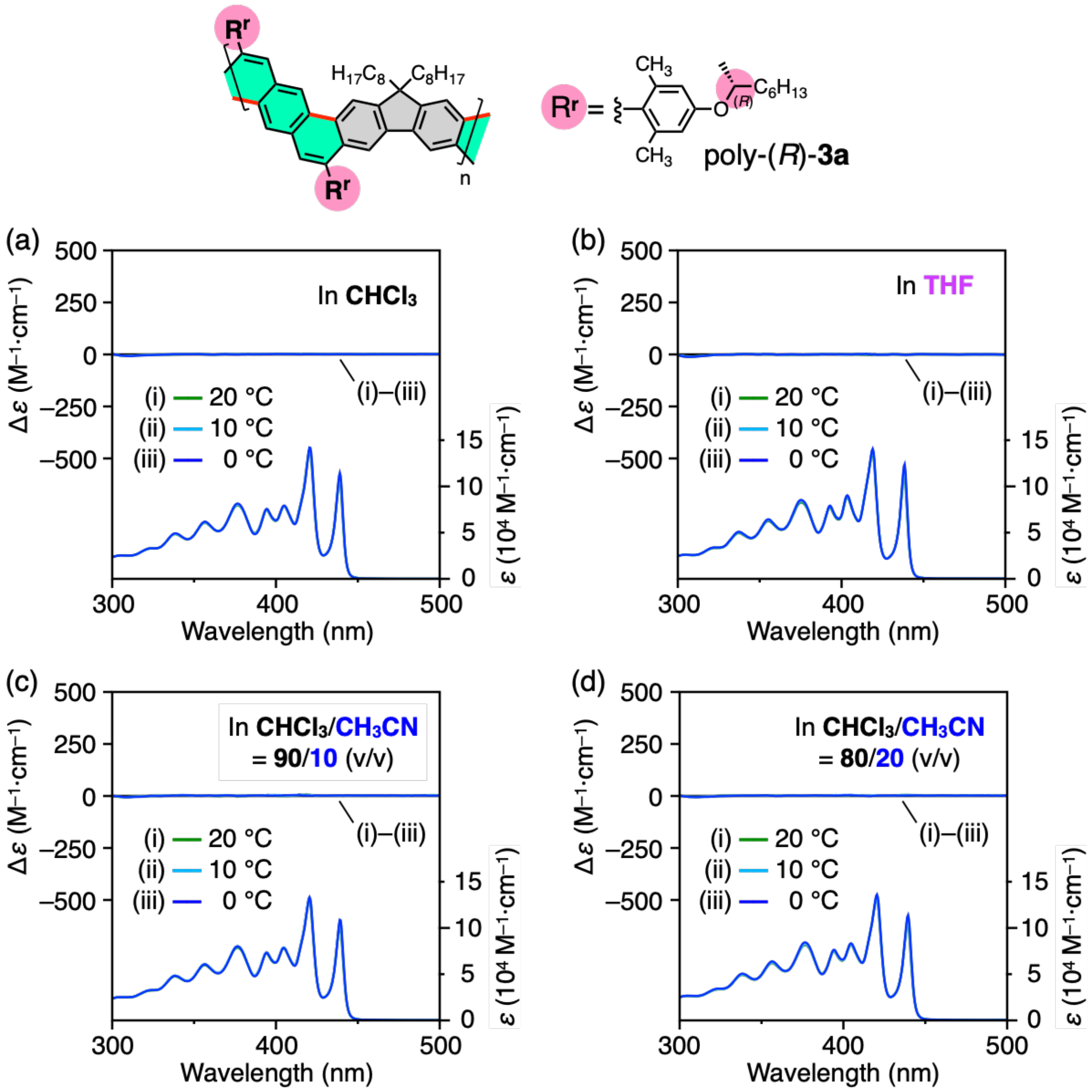

Figure S7. Absorption and CD spectra of poly-(*R*)-**3a** in chloroform (a), THF (b), and chloroform/acetonitrile (90/10 (c) and 80/20 (d), v/v) at 20 (i), 10 (ii), and 0 °C (iii). [Repeating units of polymer] = 0.10 mM.

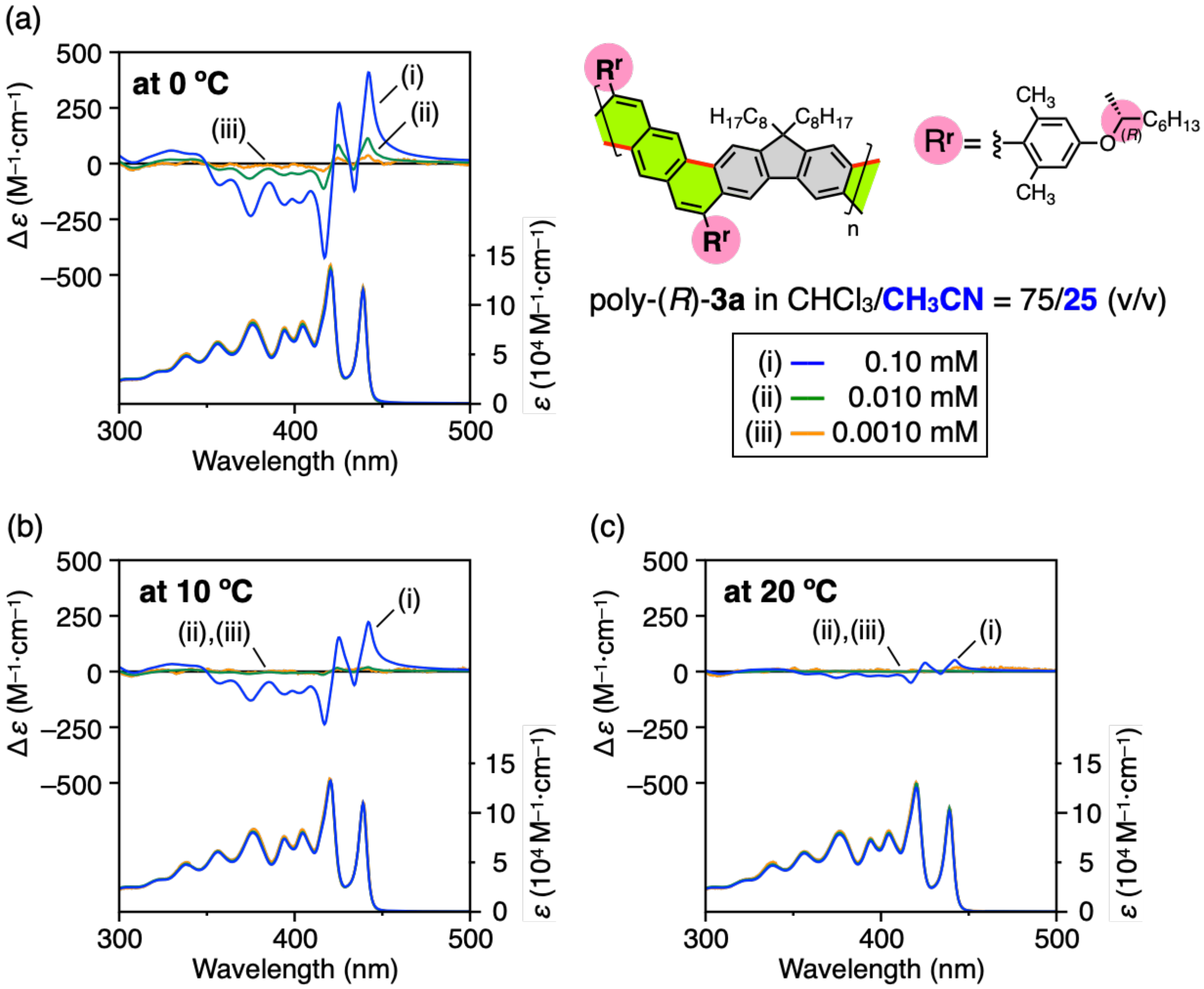

Figure S8. Absorption and CD spectra of poly-(*R*)-**3a** in chloroform/acetonitrile (75/25, v/v) at 0 (a), 10 (b), and 20 °C (c) in different concentrations ([Repeating units of polymer] = 0.10 (i), 0.010 (ii), and 0.0010 mM (iii)). The spectra were measured in 1.0- (i) and 10-mm (ii,iii) cells.

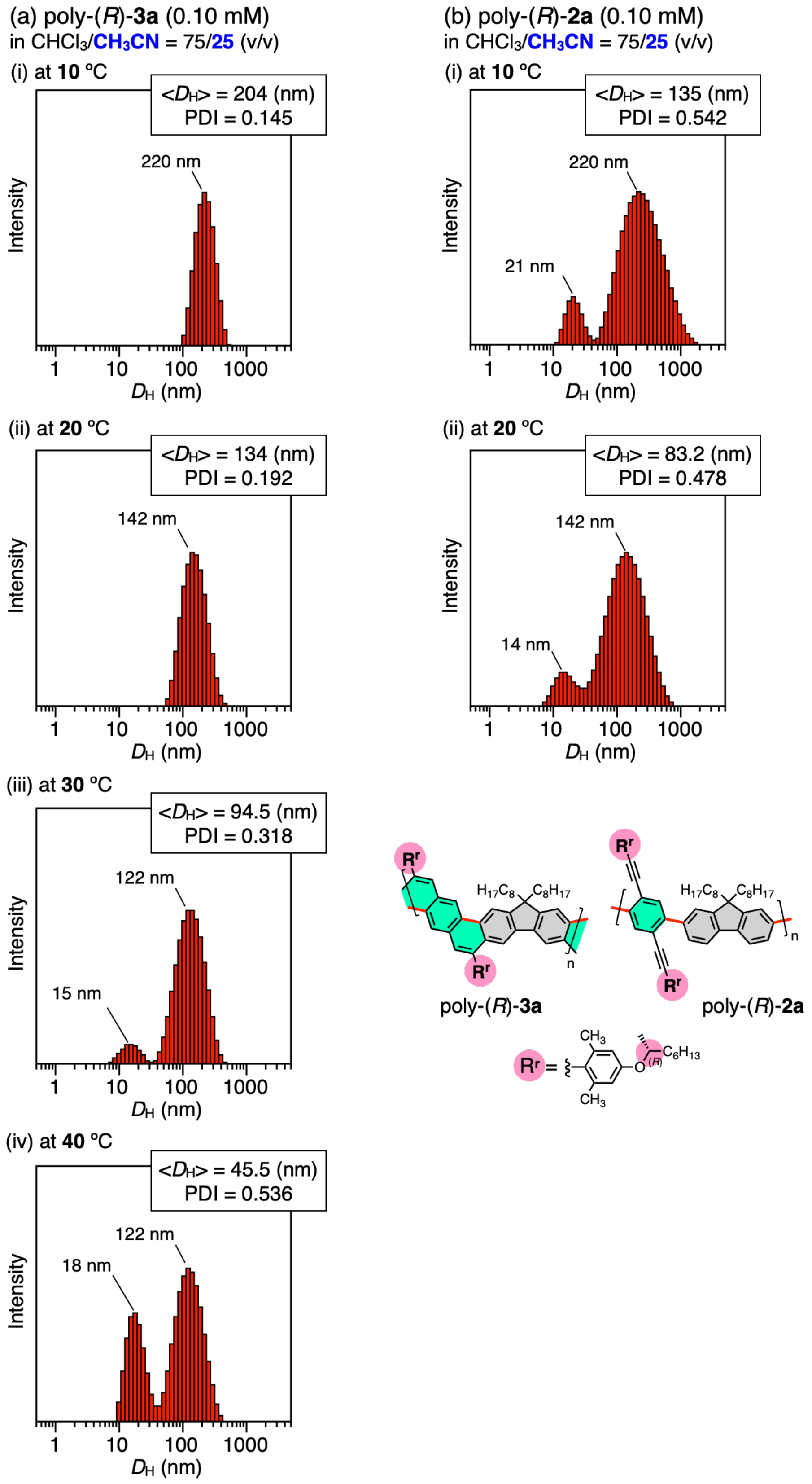

Figure S9. Histogram analyses of DLS measurements of poly-(R)-**3a** (a) and poly-(R)-**2a** (b) in chloroform/acetonitrile (75/25, v/v) at 10 (i), 20 (ii), 30 (iii), and/or 40 °C (iv). [Repeating units of polymer] = 0.10 mM.

Table S2. Hydrodynamic diameter ($<D_{H}>$) values of poly-(*R*)-**3a** and poly-(*R*)-**2a** estimated by DLS measurements in chloroform/acetonitrile (75/25, v/v) at different temperatures.

Polymer	Temperature			
	40 ºC	30 ºC	20 ºC	10 ºC
	$<D_{H}>$ (PDI)	$<D_{H}>$ (PDI)	$<D_{H}>$ (PDI)	$<D_{H}>$ (PDI)
poly-(*R*)-**3a**	45.5 nm (0.536)	94.5 nm (0.318)	134 nm (0.192)	204 nm (0.145)
poly-(*R*)-**3a** [a]	–[b]	–[b]	–[b]	–[b]
poly-(*R*)-**2a**	–[c]	–[c]	83.2 nm (0.478)	135 nm (0.542)

[a] In chloroform.

[b] Not detected.

[c] Not measured.

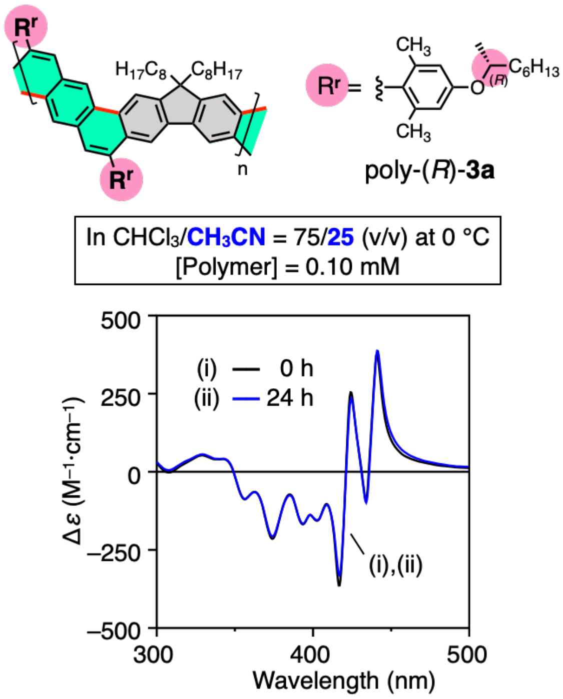

Figure S10. CD spectra of poly-(R)-**3a** in chloroform/acetonitrile (75/25, v/v) measured at 0 °C before (i) and after (ii) allowing to stand at 0 °C for 24 h. [Repeating units of polymer] = 0.10 mM.

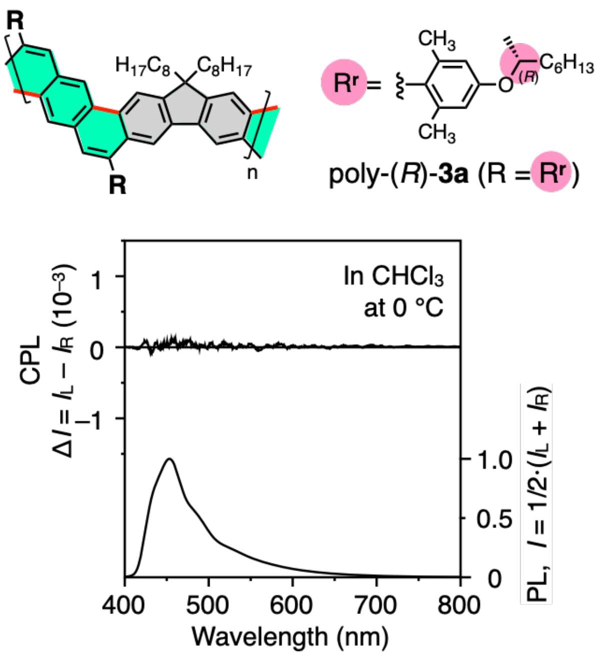

Figure S11. Normalized PL and CPL spectra (λ_{ex} = 300 nm) of poly-(*R*)-**3a** in chloroform at 0 °C. [Repeating units of polymer] = 0.10 mM.

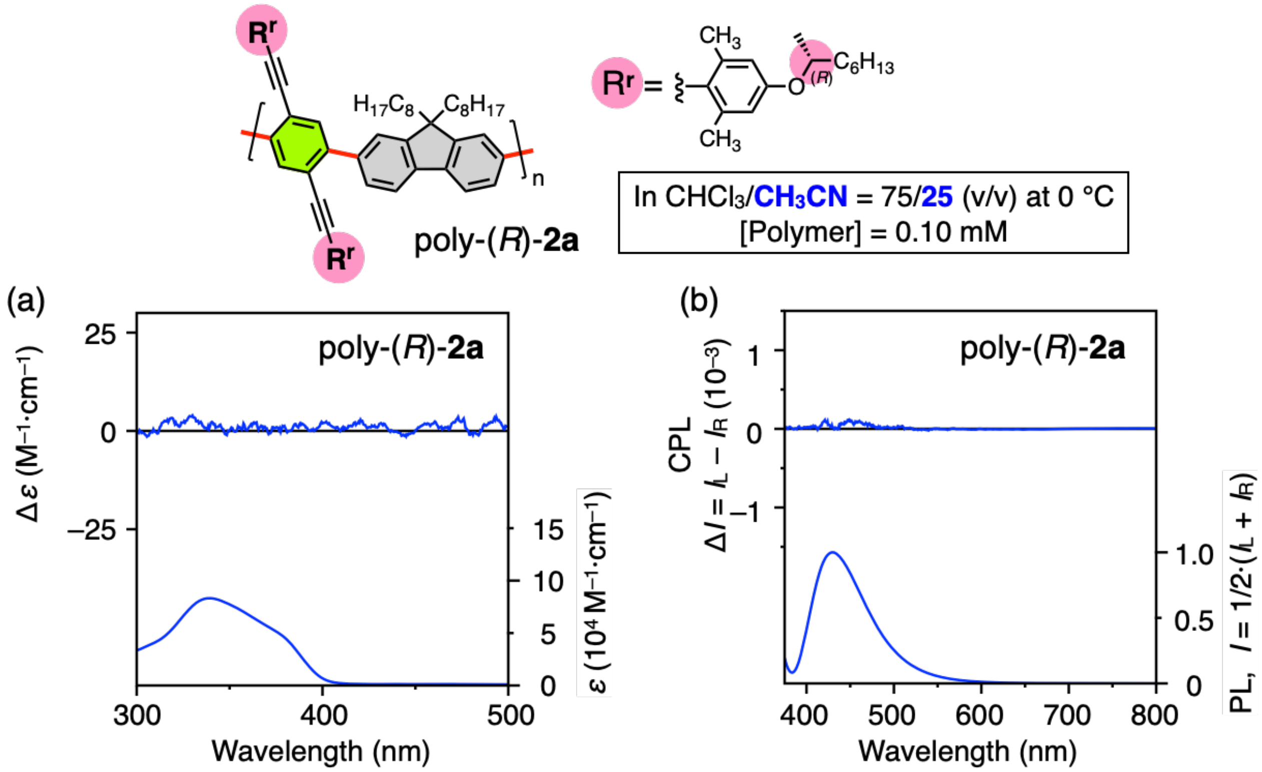

Figure S12. Absorption and CD spectra (a) and normalized PL and CPL spectra (b; λ_{ex} = 300 nm) of poly-(*R*)-**2a** in chloroform/acetonitrile (75/25, v/v) at 0 °C. [Repeating units of polymer] = 0.10 mM.

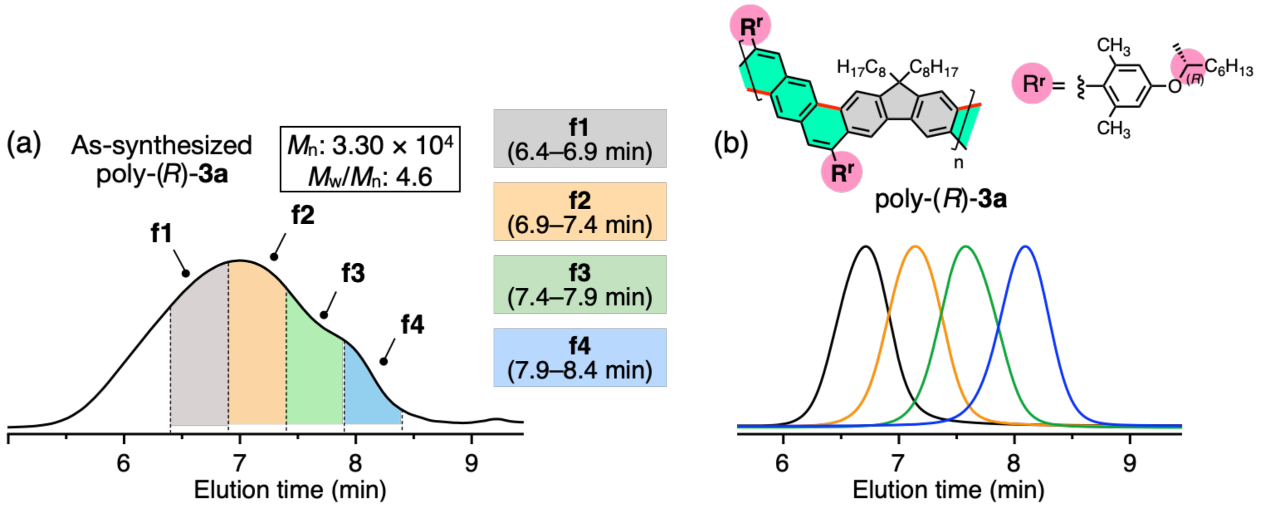

fraction	M_n (10^4)	M_w/M_n	Maximum $\|g_{abs}\|$ (10^{-1}) around 455 nm	Average $\|g_{lum}\|$ (10^{-3}) (425–550 nm)
(—) **f1**	16.2	1.3	0.88 (451 nm)	1.0
(—) **f2**	7.08	1.3	0.75 (457 nm)	0.93
(—) **f3**	2.95	1.3	0.12 (456 nm)	0.11
(—) **f4**	1.13	1.2	< 0.1 (458 nm)	< 0.1
As-synthesized poly-(*R*)-**3a**	3.30	4.6	0.48 (456 nm)	0.86

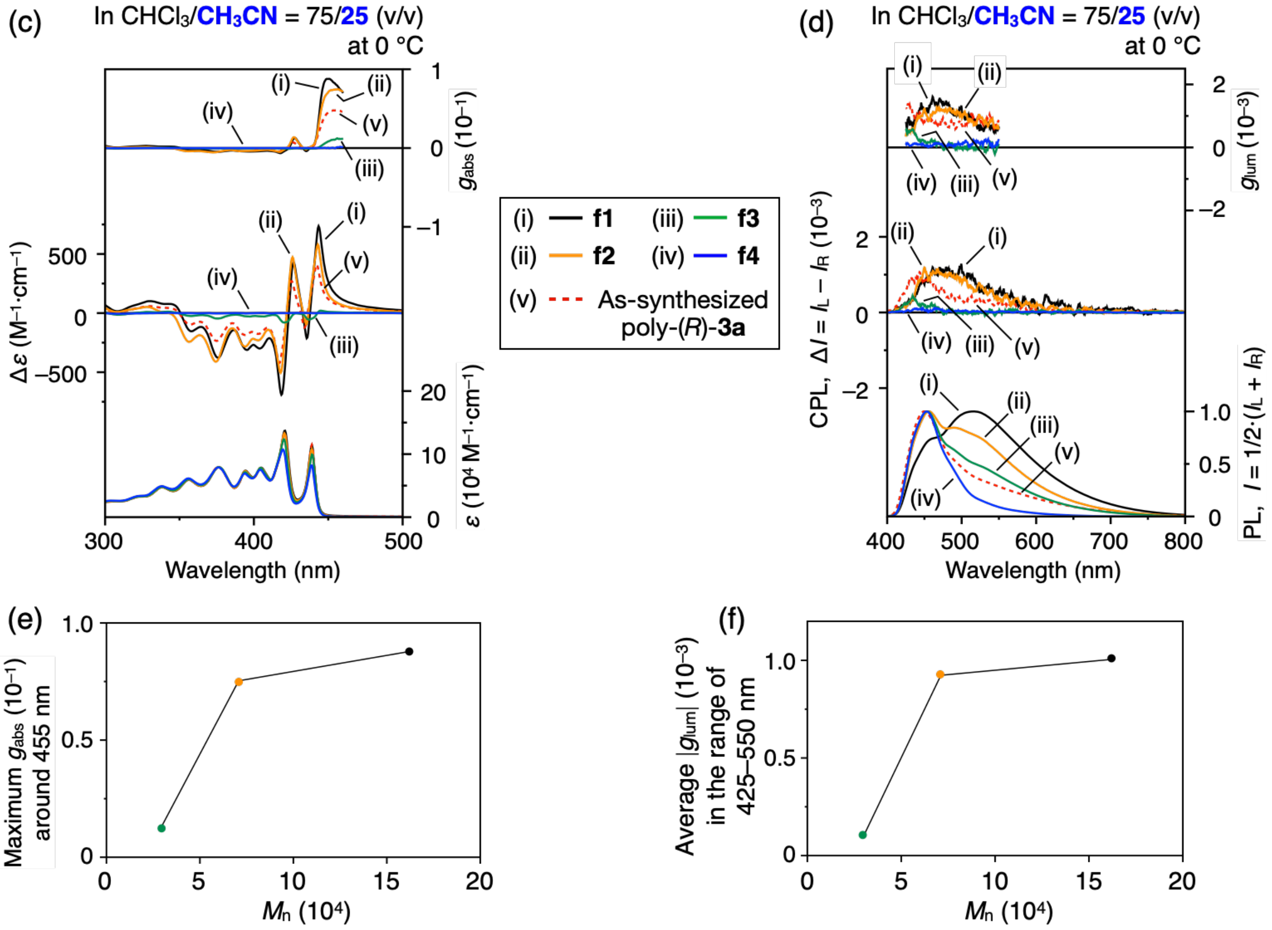

Figure S13. SEC traces of the as-synthesized poly-(*R*)-**3a** (a) and its fractionated components (**f1** – **f4**) with different molar masses (b) (eluent, chloroform; polystyrene standards). (c) Absorption

(bottom), CD (middle), and g_{abs} (top) spectra of fractionated poly-(*R*)-**3a** (**f1** – **f4**) measured in chloroform/acetonitrile (75/25, v/v) at 0 °C after SEC fractionation. (d) Normalized PL (bottom), CPL (middle), and *g*lum (top) spectra of fractionated poly-(*R*)-**3a** (**f1** – **f4**) measured in chloroform/acetonitrile (75/25, v/v) at 0 °C after SEC fractionation. λ_{ex} = 300 nm. The corresponding spectra of the as-synthesized poly-(*R*)-**3a** measured in chloroform/acetonitrile (75/25, v/v) at 0 °C are also shown in (c,d). [Repeating units of polymer] = 0.10 mM. (e) Plots of maximum Kuhn's dissymmetry factors (g_{abs}) around 455 nm of poly-(*R*)-**3a** versus the M_n value. (f) Plots of luminescence dissymmetry factors (g_{lum}) of poly-(*R*)-**3a**, which are estimated as average values in the range of 425–550 nm due to the relatively large wavelength-dependent variation, versus the M_n value. The g_{lum} values are defined as $2(I_L - I_R)/(I_L + I_R)$, where I_L and I_R are the PL intensities of the left- and right-handed circularly polarized light, respectively.

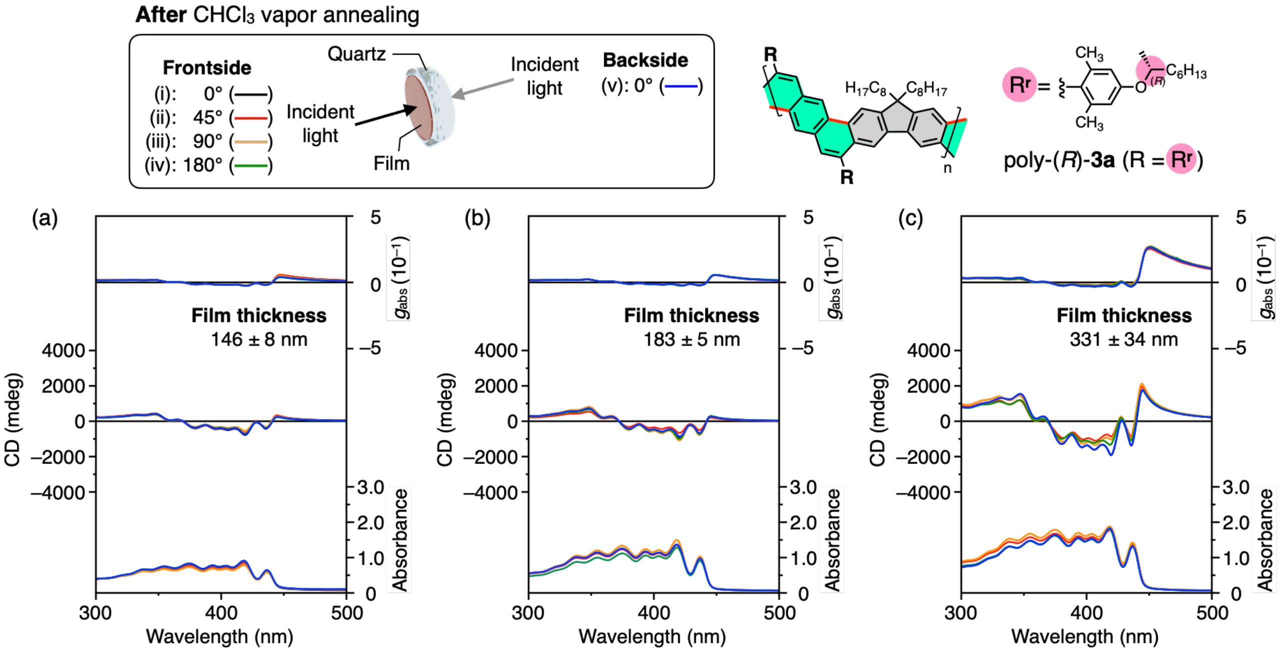

Figure S14. Absorption (bottom), CD (middle), and g_{abs} (top) spectra of the poly-(*R*)-**3a** films with different thicknesses (146 (a), 183 (b), and 331 (c)) measured at room temperature after annealing in chloroform vapor at 25 °C for 1 h. The pristine polymer thin films were prepared by drop-casting of the corresponding chloroform/acetonitrile (75/25, v/v) solutions (0.40 (a), 0.60 (b), and 1.0 mM (c)). All spectra were measured at different rotation angles (0°: black (i), 45°: red (ii), 90°: yellow (iii), and 180°: green (iv) lines) and by reversing the quartz plate (0°: blue lines (v)). For the corresponding data of the as-cast films, see Figure S15.

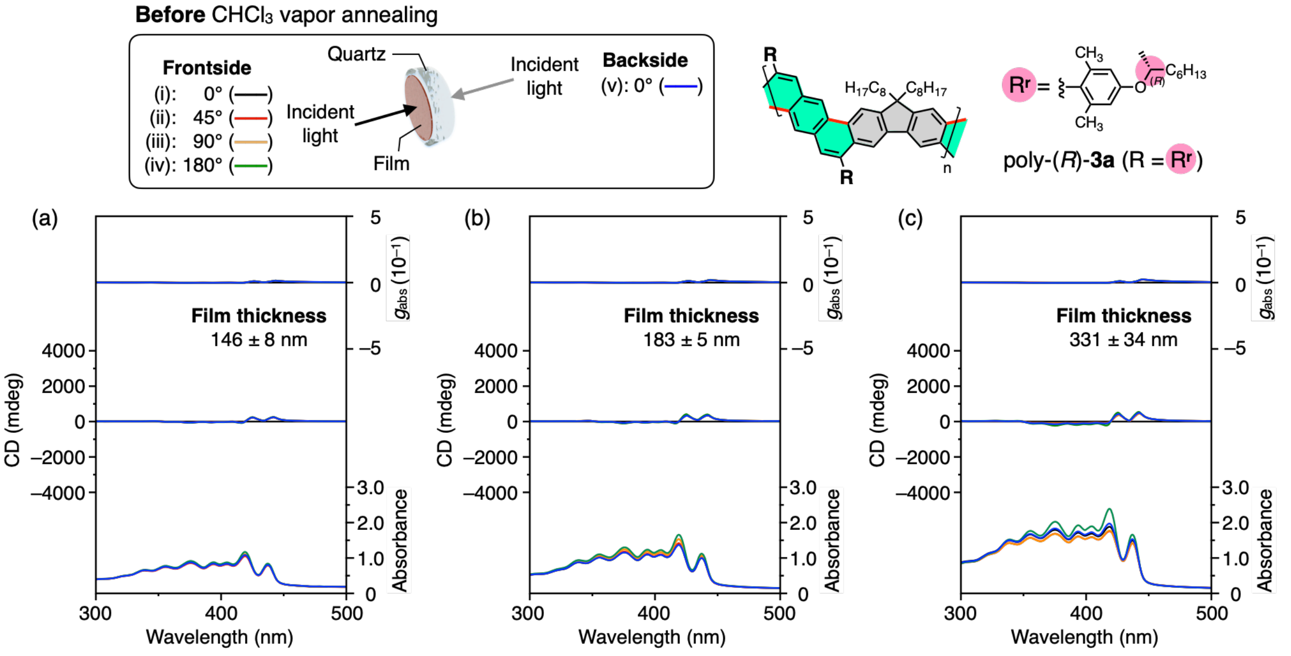

Figure S15. Absorption (bottom), CD (middle), and g_{abs} (top) spectra of the as-cast poly-(*R*)-**3a** films with different thicknesses (146 (a), 183 (b), and 331 (c)) measured at room temperature. The pristine polymer thin films were prepared by drop-casting of the corresponding chloroform/acetonitrile (75/25, v/v) solutions (0.40 (a), 0.60 (b), and 1.0 mM (c)). Film thicknesses shown here were measured after chloroform vapor exposure. All spectra were measured at different rotation angles (0°: black (i), 45°: red (ii), 90°: yellow (iii), and 180°: green (iv) lines) and by reversing the quartz plate (0°: blue lines (v)).

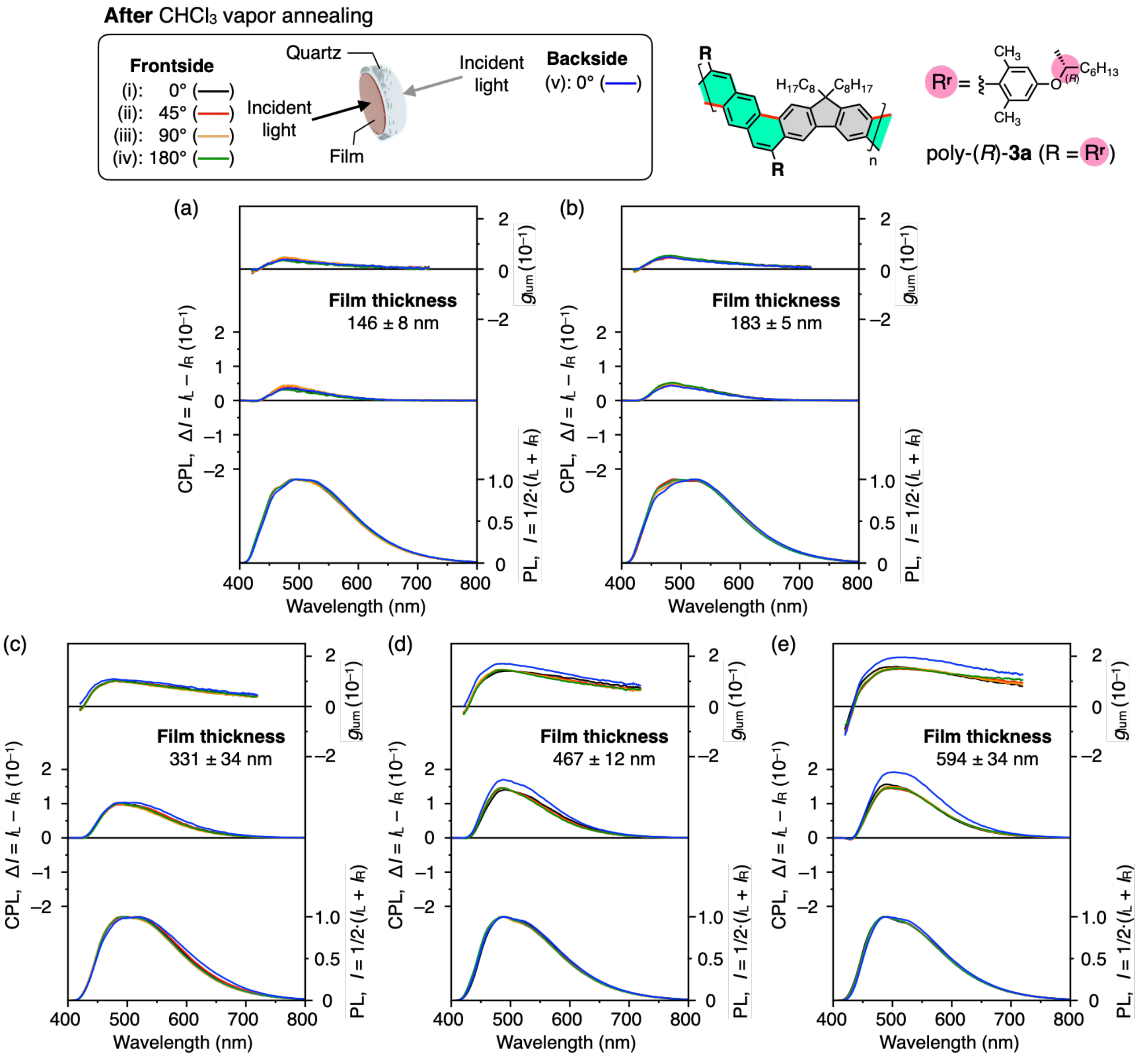

Figure S16. Normalized PL (bottom), CPL (middle), and g_{lum} (top) spectra (λ_{ex} = 300 nm) of the poly-(*R*)-**3a** films with different thicknesses (146 (a), 183 (b), 331 (c), 467 (d), and 594 nm (e)) measured at room temperature after annealing in chloroform vapor at 25 °C for 1 h. The pristine polymer thin films were prepared by drop-casting of the corresponding chloroform/acetonitrile (75/25, v/v) solutions (0.40 (a), 0.60 (b), 1.0 (c), 1.5 (d), and 2.0 mM (e)). All spectra were measured at different rotation angles (0°: black (i), 45°: red (ii), 90°: yellow (iii), and 180°: green (iv) lines) and by reversing the quartz plate (0°: blue lines (v)). For the corresponding data of the as-cast films, see Figure S17.

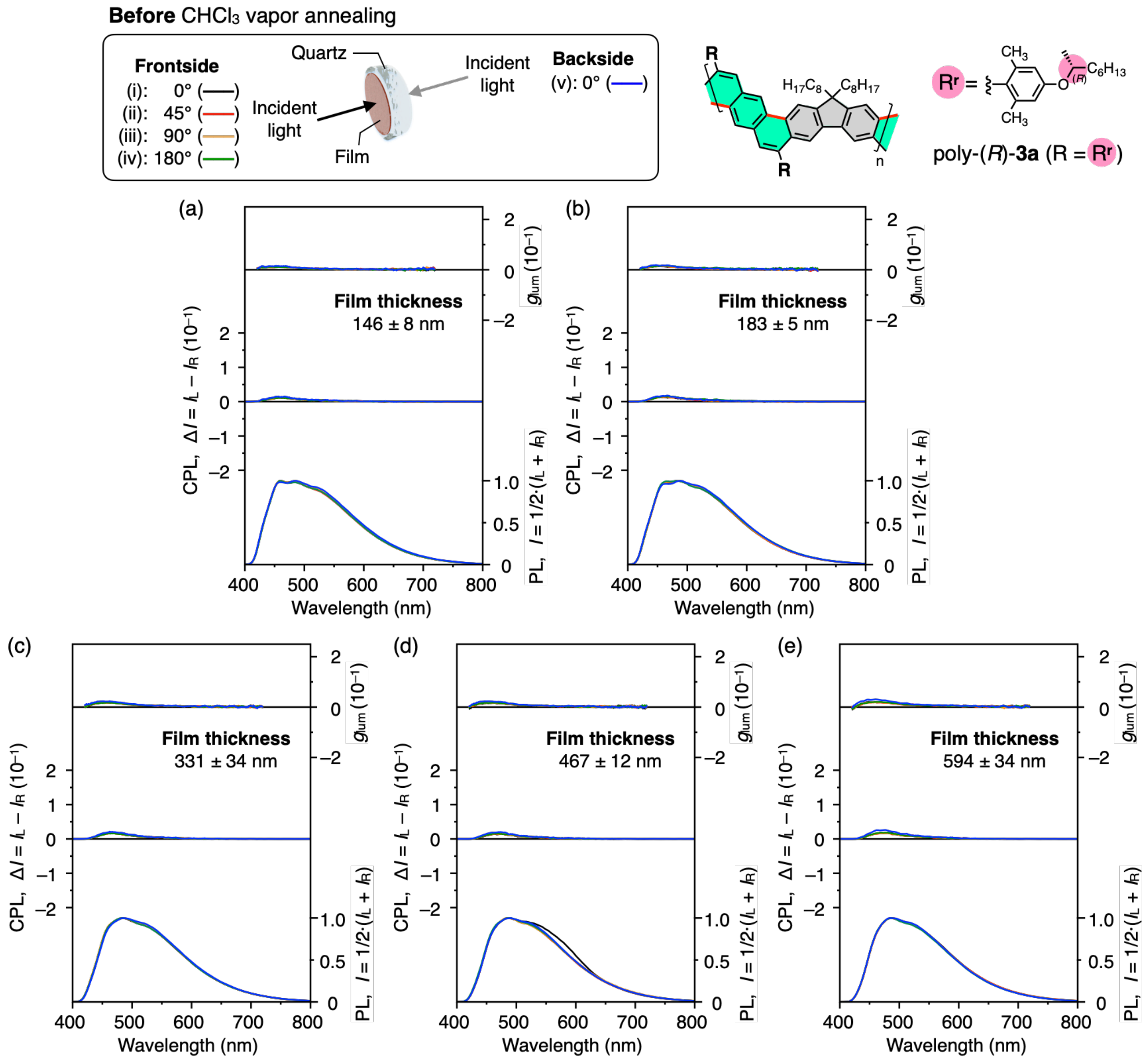

Figure S17. Normalized PL (bottom), CPL (middle), and g_{lum} (top) spectra (λ_{ex} = 300 nm) of the as-cast poly-(*R*)-**3a** films with different thicknesses (146 (a), 183 (b), 331 (c), 467 (d), and 594 nm (e)) measured at room temperature. The pristine polymer thin films were prepared by drop-casting of the corresponding chloroform/acetonitrile (75/25, v/v) solutions (0.40 (a), 0.60 (b), 1.0 (c), 1.5 (d), and 2.0 mM (e)). Film thicknesses shown here were measured after chloroform vapor exposure. All spectra were measured at different rotation angles (0°: black (i), 45°: red (ii), 90°: yellow (iii), and 180°: green (iv) lines) and by reversing the quartz plate (0°: blue lines (v)).

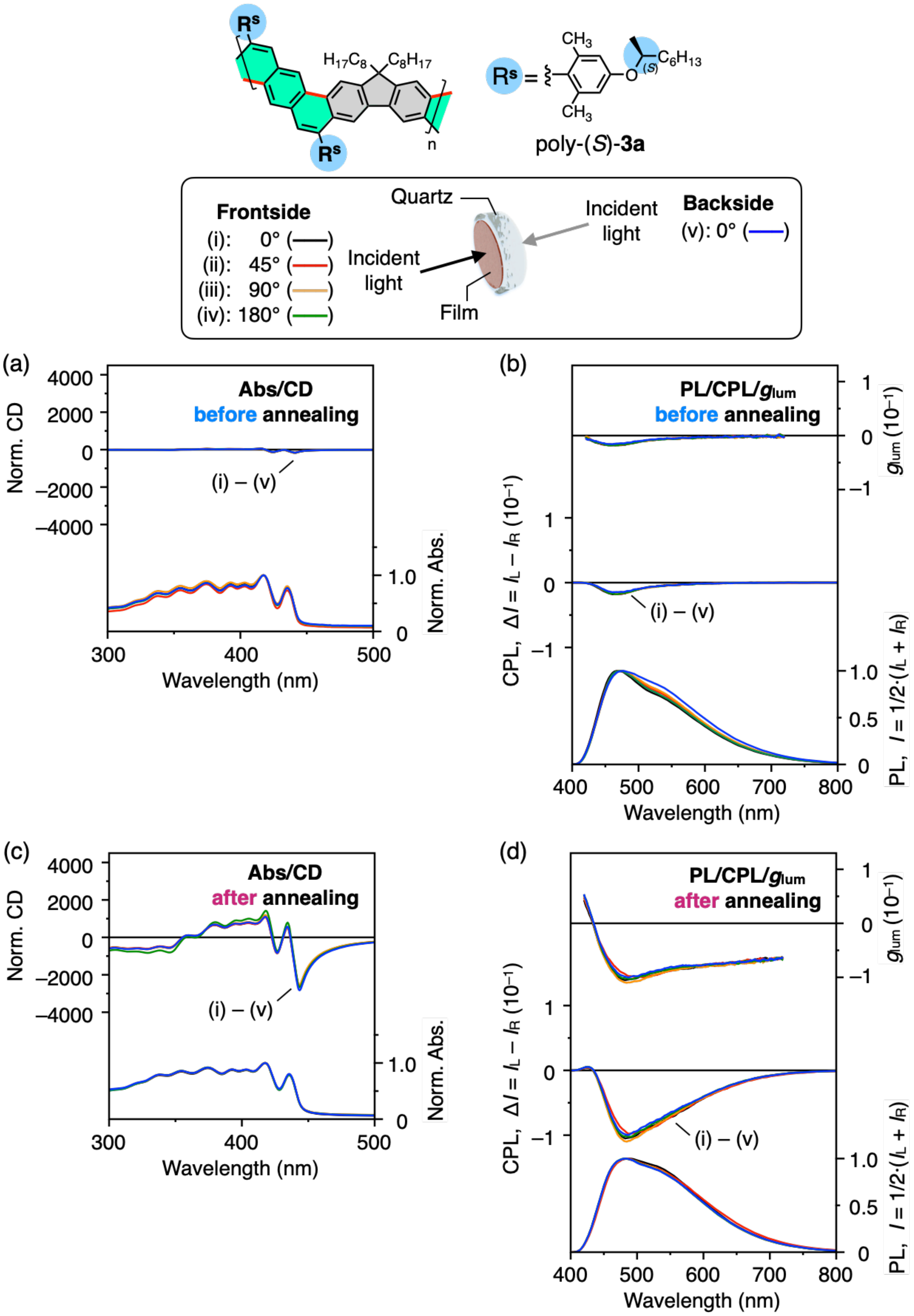

Figure S18. Normalized absorption and CD spectra (a,c) and normalized PL (bottom), CPL (middle), and g_{lum} (top) spectra (b,d; λ_{ex} = 300 nm) of poly-(*S*)-**3a** in the film state measured at room temperature before (a,b) and after (c,d) annealing in chloroform vapor at 25 °C for 1 h. The pristine polymer thin film was prepared by drop-casting of the corresponding chloroform/acetonitrile (75/25, v/v) solution (1.0 mM). The CD and absorption spectra were normalized based on the corresponding absorption spectra at room temperature. All spectra were measured at different rotation angles (0°: black (i), 45°: red (ii), 90°: yellow (iii), and 180°: green (iv) lines) and by reversing the quartz plate (0°: blue lines (v)).

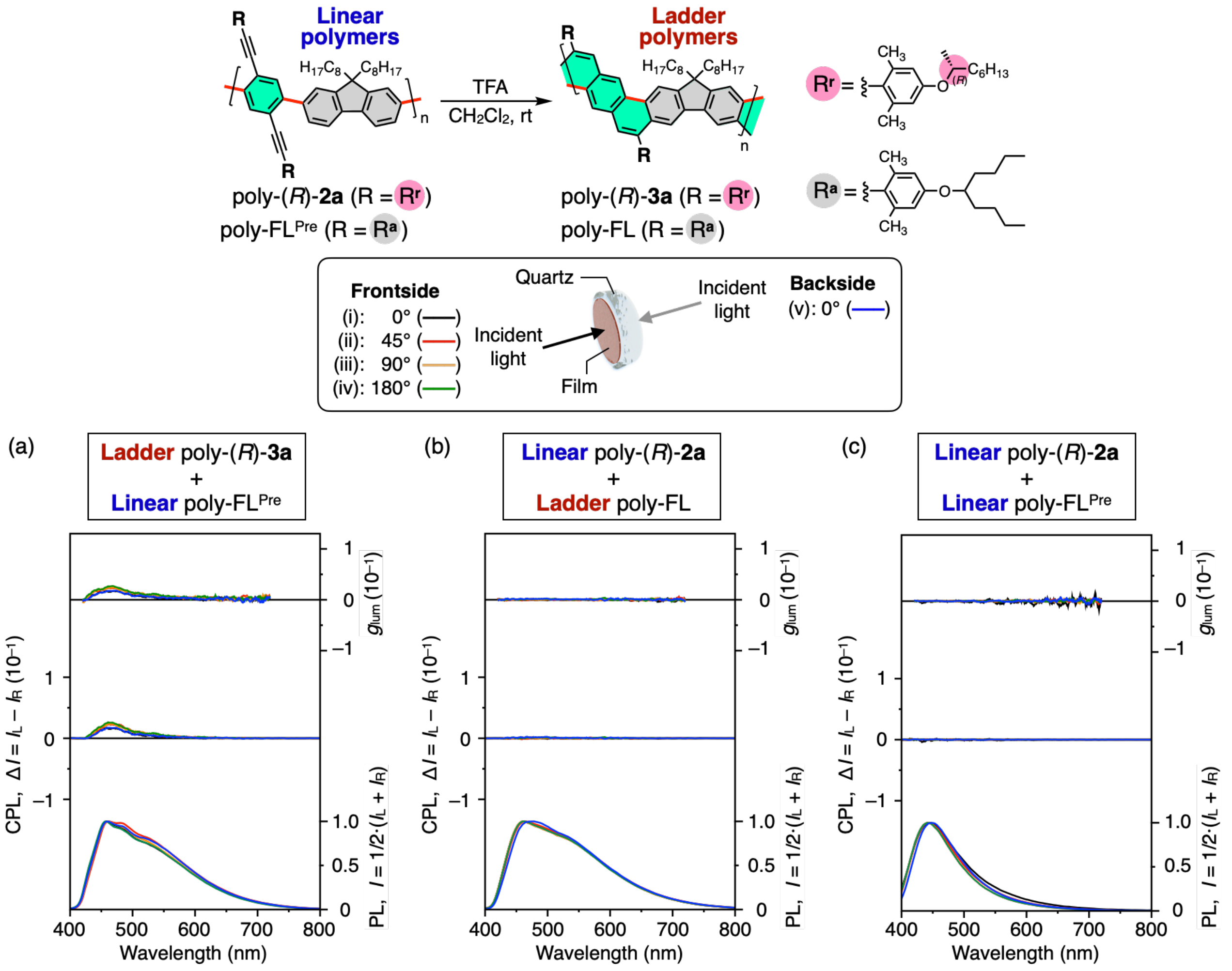

Figure S19. Normalized PL (bottom), CPL (middle), and g_{lum} (top) spectra (λ_{ex} = 300 nm) of the poly-(*R*)-**3a**/poly-FL^{Pre} (a), poly-(*R*)-**2a**/poly-FL (b), and poly-(*R*)-**2a**/poly-FL^{Pre} (c) blended films (50/50, mol/mol) measured at room temperature after annealing in chloroform vapor at 25 °C for 1 h. The pristine polymer thin films were prepared by drop-casting of the corresponding chloroform/acetonitrile (75/25, v/v) solutions (1.0 mM). All spectra were measured at different rotation angles (0°: black (i), 45°: red (ii), 90°: yellow (iii), and 180°: green (iv) lines) and by reversing the quartz plate (0°: blue lines (v)).

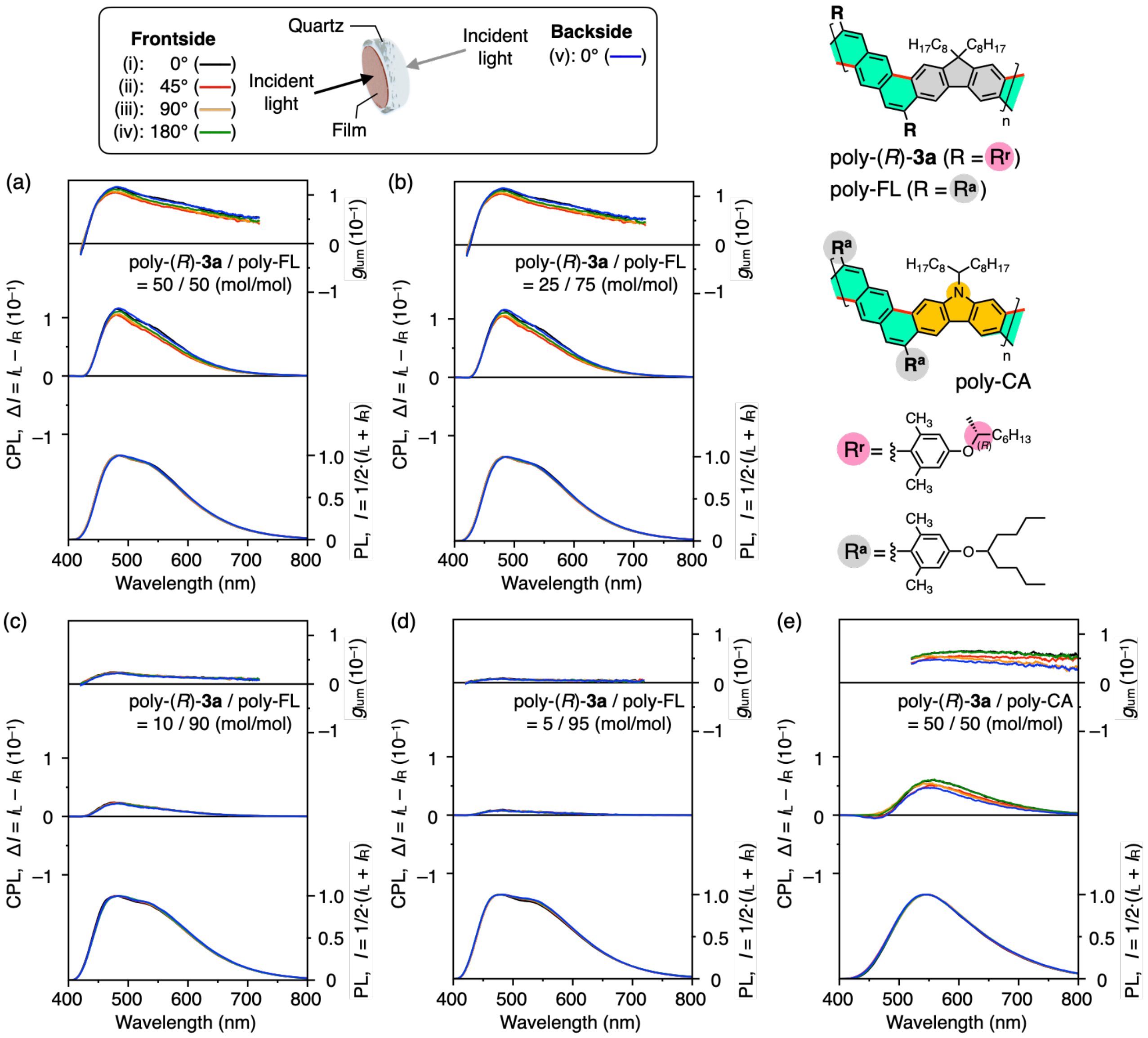

Figure S20. Normalized PL (bottom), CPL (middle), and g_{lum} (top) spectra (λ_{ex} = 300 nm) of the polymer blended films containing poly-(*R*)-**3a** and poly-FL at different molar ratios (50/50 (a), 25/75 (b), 10/90 (c), and 5/95 (d), mol/mol) and the poly-(*R*)-**3a**/poly-CA blended film (50/50, mol/mol) (e) measured at room temperature after annealing in chloroform vapor at 25 °C for 1 h. The pristine polymer thin films were prepared by drop-casting of the corresponding chloroform/acetonitrile (75/25 (a–d) or 100/0 (e), v/v) solutions (1.0 mM). All spectra were measured at different rotation angles (0°: black (i), 45°: red (ii), 90°: yellow (iii), and 180°: green (iv) lines) and by reversing the quartz plate (0°: blue lines (v)).

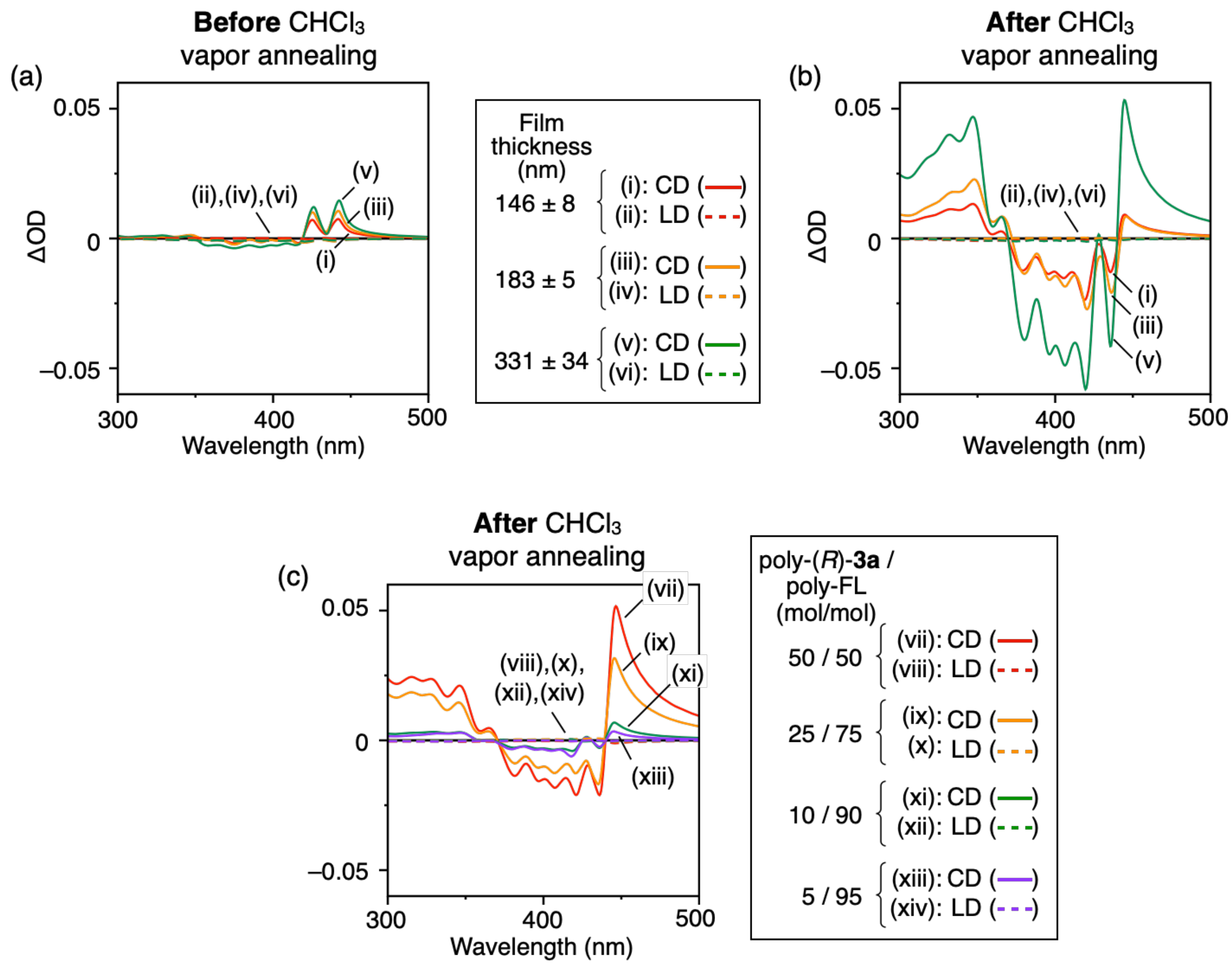

Figure S21. CD (i, iii, v) and linear dichroism (LD) (ii, iv, vi) spectra of the poly-(*R*)-**3a** films with different thicknesses (146 (i,ii), 183 (iii,iv), and 331 nm (v,vi)) measured at room temperature before (a) and after (b) annealing in chloroform vapor at 25 °C for 1 h, expressed as Δoptical density (ΔOD). The pristine polymer thin films were prepared by drop-casting of the corresponding chloroform/acetonitrile (75/25, v/v) solutions (0.40 (i,ii), 0.60 (iii,iv), and 1.0 mM (v,vi)). Film thicknesses shown here were measured after chloroform vapor exposure. (c) CD (vii,ix,xi,xiii) and LD (viii,x,xii,xiv) spectra of the polymer blended films containing poly-(*R*)-**3a** and poly-FL at different molar ratios (50/50 (vii,viii), 25/75 (ix,x), 10/90 (xi,xii), and 5/95 (xiii,xiv), mol/mol), expressed as ΔOD. Measurements were performed at room temperature after annealing in chloroform vapor at 25 °C for 1 h. The pristine polymer thin films were prepared by drop-casting of the corresponding chloroform/acetonitrile (75/25, v/v) solutions (1.0 mM).

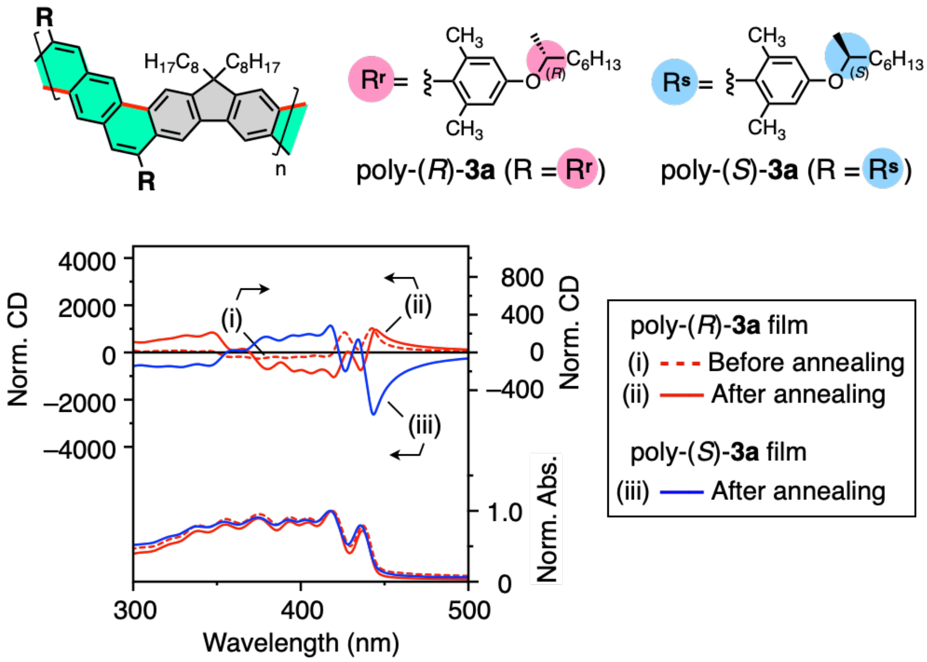

Figure S22. Normalized absorption and CD spectra of poly-(*R*)-**3a** (i,ii) and poly-(*S*)-**3a** (iii) in the film state measured at room temperature before (i) and after (ii,iii) annealing in chloroform vapor at 25 °C for 1 h. The pristine polymer thin films were prepared by drop-casting of the corresponding chloroform/acetonitrile (75/25, v/v) solutions (1.0 mM). The CD and absorption spectra were normalized based on the corresponding absorption spectra at room temperature.

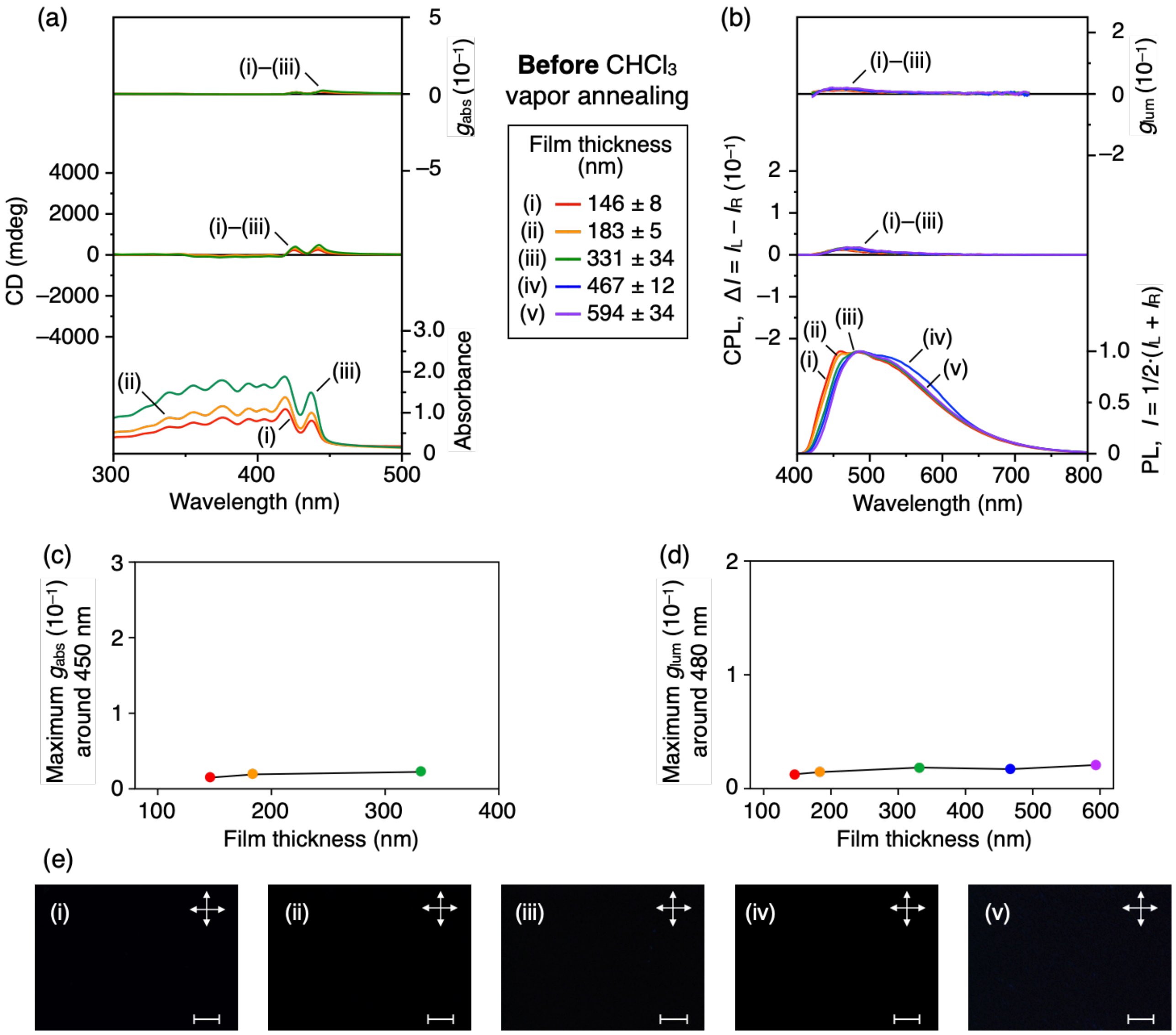

Figure S23. (a,b) Absorption (bottom), CD (middle), and g_{abs} (top) spectra (a) and normalized PL (bottom), CPL (middle), and g_{lum} (top) spectra (b; λ_{ex} = 300 nm) of the as-cast poly-(*R*)-**3a** films with different thicknesses (146 (i), 183 (ii), 331 (iii), 467 (iv), and/or 594 nm (v)) measured at room temperature. The pristine polymer thin films were prepared by drop-casting of the corresponding chloroform/acetonitrile (75/25, v/v) solutions (0.40 (i), 0.60 (ii), 1.0 (iii), 1.5 (iv), and/or 2.0 mM (v)). Film thicknesses shown here were measured after chloroform vapor exposure. (c,d) Plots of the maximum g_{abs} (c; $\lambda \approx 450$ nm) and g_{lum} (d; $\lambda \approx 480$ nm) values of the poly-(*R*)-**3a** films versus the film thickness. (e) Polarized optical micrographs of the as-cast poly-(*R*)-**3a** films taken at room temperature under crossed polarizers. Scale bar: 50 μm.

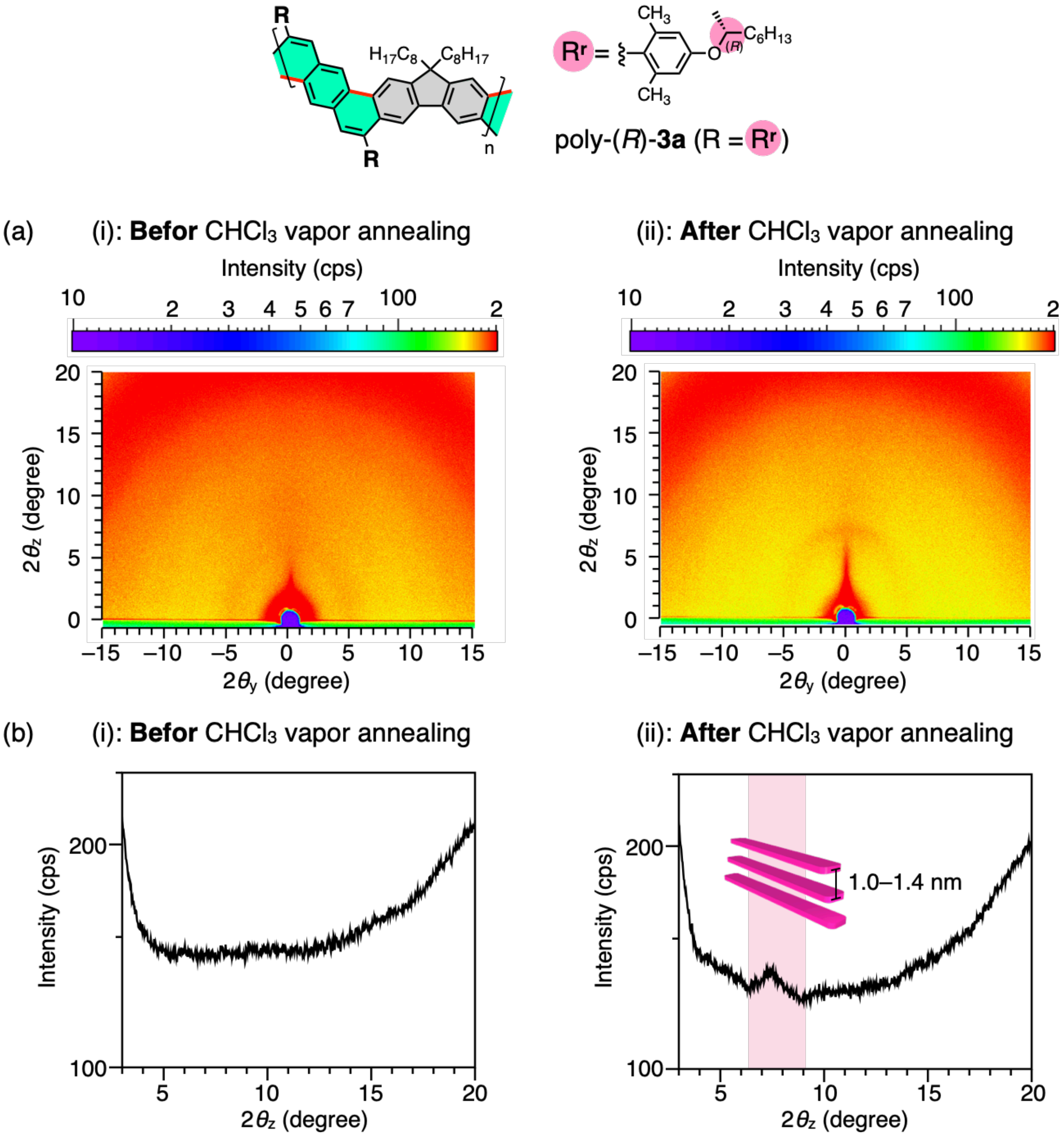

Figure S24. (a) 2D-WAXS images of the poly-(R)-**3a** film before (i) and after (ii) annealing in chloroform vapor at 25 °C for 1 h. The pristine polymer thin film was prepared by drop-casting of the corresponding chloroform/acetonitrile (75/25, v/v) solution (1.0 mM). (b) The corresponding out-of-plane line-cut profiles.

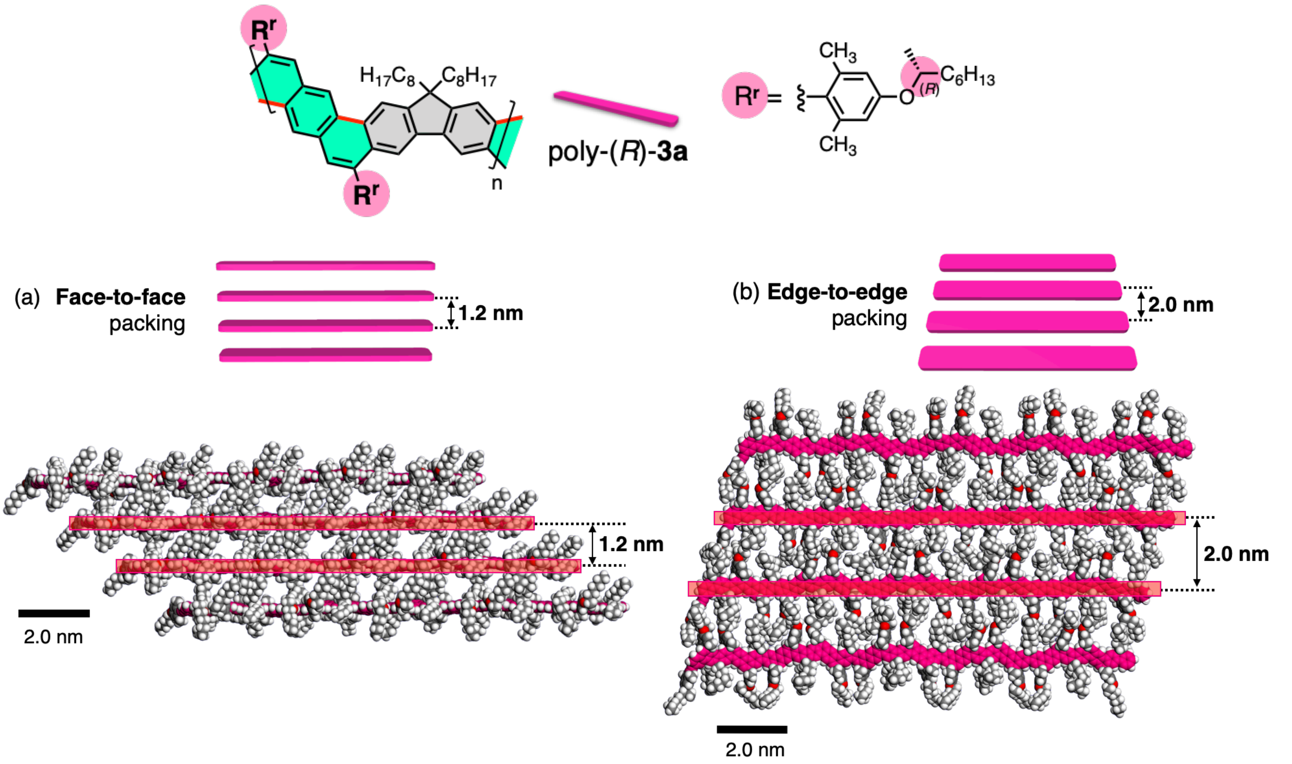

Figure S25. Side (a) and top (b) views of possible face-to-face (a) and edge-to-edge (b) packing structures of poly-(*R*)-**3a**, respectively. The structures are represented by space-filling models and the main-chain atoms are shown in purple for clarity.

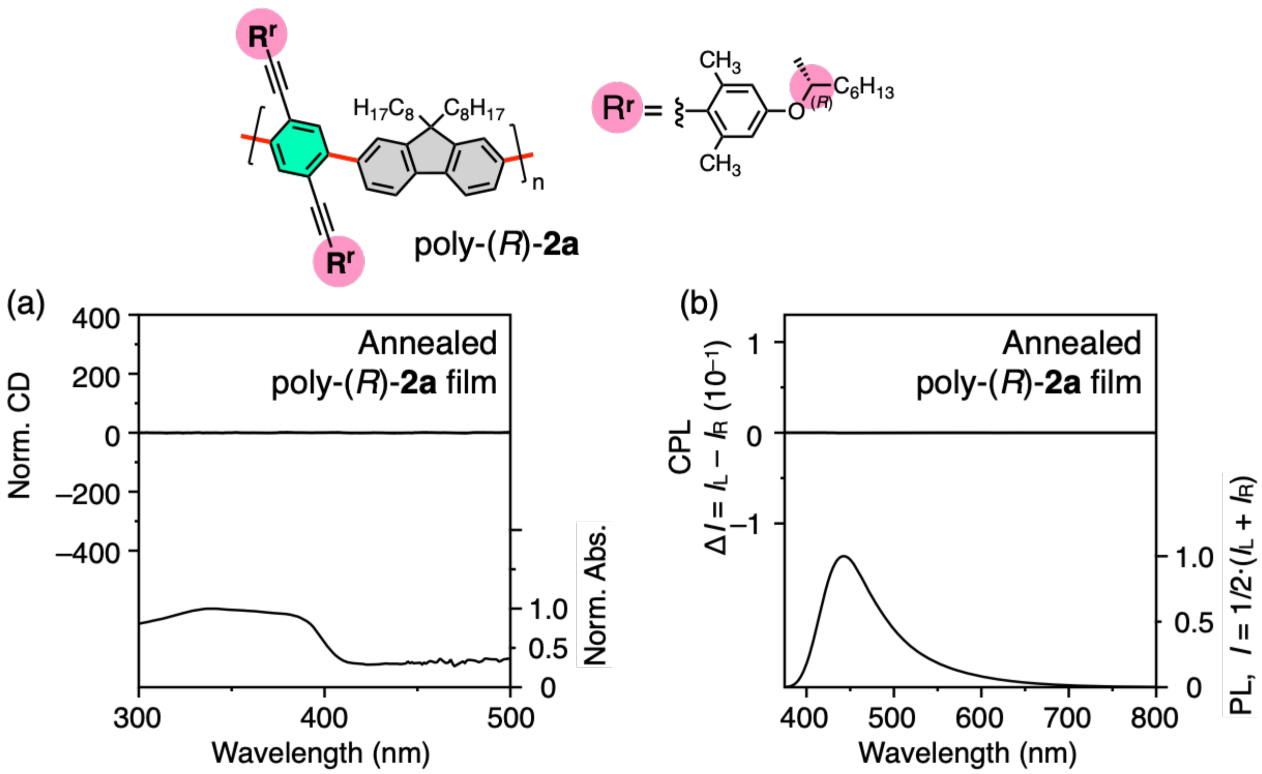

Figure S26. Normalized absorption and CD spectra (a) and normalized PL and CPL spectra (b; λ_{ex} = 300 nm) of poly-(*R*)-**2a** in the film state measured at room temperature after annealing in chloroform vapor at 25 °C for 1 h. The pristine polymer thin film was prepared by drop-casting of the corresponding chloroform/acetonitrile (75/25, v/v) solution (1.0 mM). The CD and absorption spectra were normalized based on the corresponding absorption spectrum at room temperature.

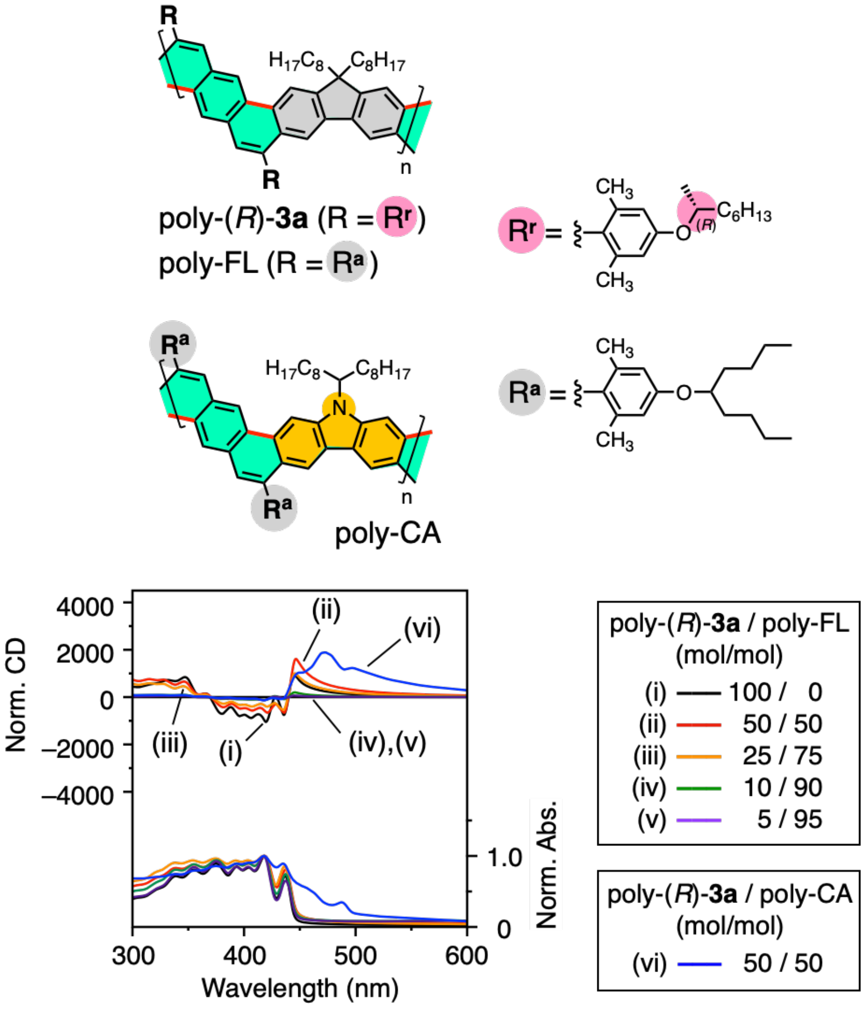

Figure S27. Normalized absorption and CD spectra of the polymer blended films containing poly-(*R*)-**3a** and poly-FL at different molar ratios (100/0 (i), 50/50 (ii), 25/75 (iii), 10/90 (iv), and 5/95 (v), mol/mol) and the poly-(*R*)-**3a**/poly-CA blended film (50/50, mol/mol) (vi). Measurements were performed at room temperature after annealing in chloroform vapor at 25 °C for 1 h. The pristine polymer thin films were prepared by drop-casting of the corresponding chloroform/acetonitrile (75/25 (i–v) or 100/0 (vi), v/v) solutions (1.0 mM). The CD and absorption spectra were normalized based on the corresponding absorption spectra at room temperature.

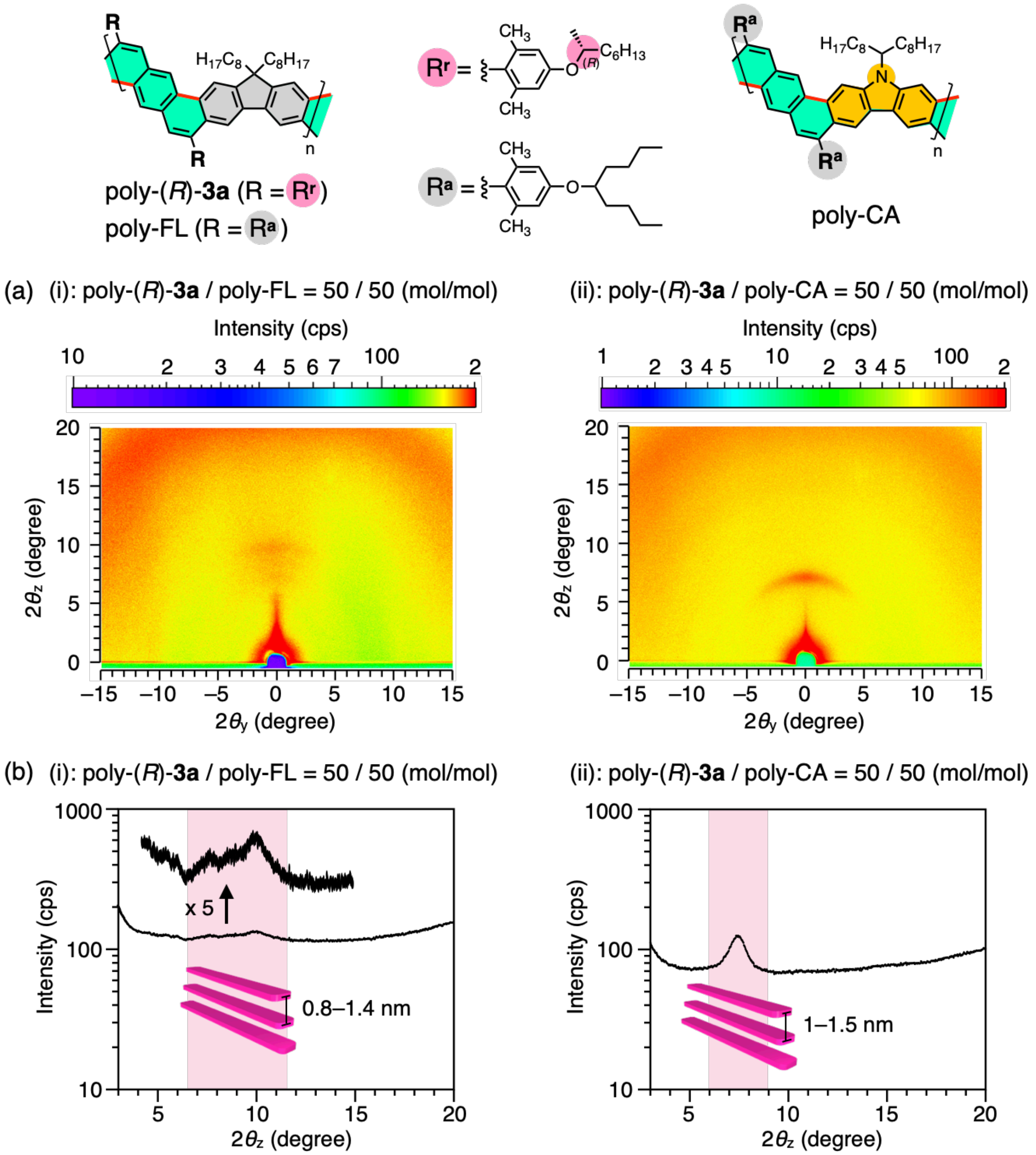

Figure S28. (a) 2D-WAXS images of the poly-(*R*)-**3a**/poly-FL (i) and poly-(*R*)-**3a**/poly-CA (ii) blended films (50/50, mol/mol) after annealing in chloroform vapor at 25 °C for 1 h. The pristine polymer thin films were prepared by drop-casting of the corresponding chloroform/acetonitrile (75/25 (i) or 100/0 (ii), v/v) solutions (1.0 mM). (b) The corresponding out-of-plane line-cut profiles.

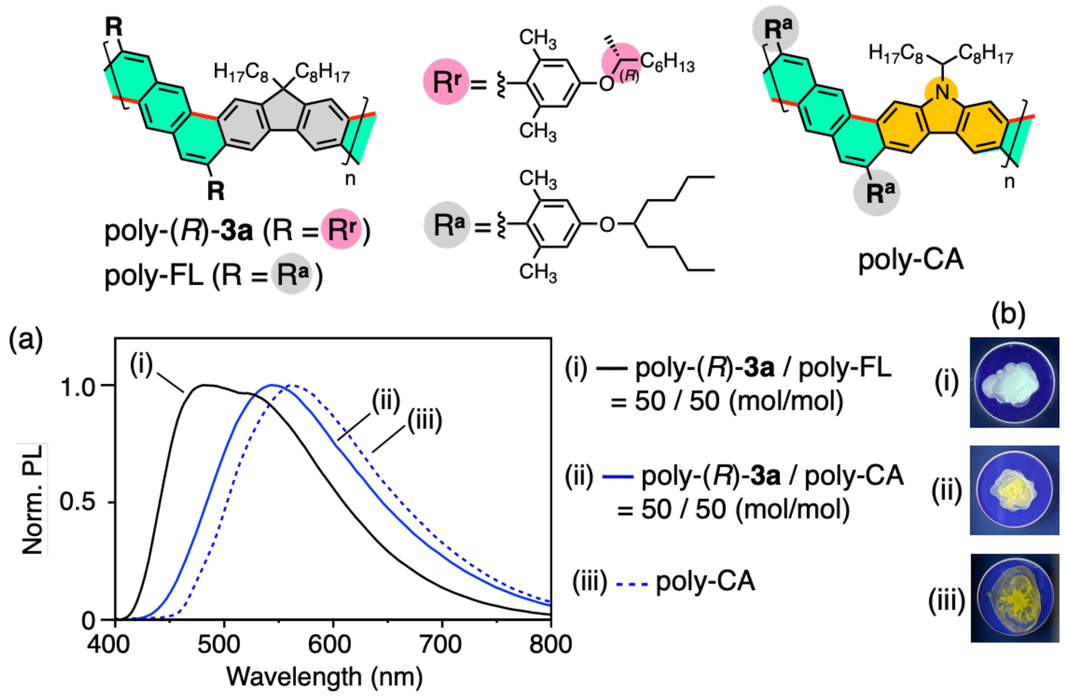

Figure S29. (a) Normalized PL spectra (λ_{ex} = 300 nm) of the poly-(*R*)-**3a**/poly-FL (i) and poly-(*R*)-**3a**/poly-CA (ii) blended films (50/50, mol/mol) and the poly-CA film (iii) after annealing in chloroform vapor at 25 °C for 1 h. The pristine polymer thin films were prepared by drop-casting of the corresponding chloroform/acetonitrile (75/25 (i) or 100/0 (ii,iii), v/v) solutions (1.0 mM). (b) Photographs of the corresponding films under irradiation at 365 nm.

5. Supporting References

[S1] P. J. M. Stals, M. M. J. Smulders, R. Martín-Rapún, A. R. A. Palmans, E. W. Meijer, *Chem.-Eur. J.* **2009**, *15*, 2071–2080.

[S2] J. E. Roque Peña, E. J. Alexanian, *Org. Lett.* **2017**, *19*, 4413–4415.

[S3] W. Zheng, T. Ikai, E. Yashima, *Angew. Chem., Int. Ed.* **2021**, *60*, 11294–11299.

[S4] H. Sun, Z. Jin, C. Yang, R. L. C. Akkermans, S. H. Robertson, N. A. Spenley, S. Miller, S. M. Todd, *J. Mol. Model.* **2016**, *22*, 47.

[S5] a) J. Lee, B. B. Rajeeva, T. Yuan, Z.-H. Guo, Y.-H. Lin, M. Al-Hashimi, Y. Zheng, L. Fang, *Chem. Sci.* **2016**, *7*, 881–889; b) J. Lee, A. J. Kalin, C. Wang, J. T. Early, M. Al-Hashimi, L. Fang, *Polym. Chem.* **2018**, *9*, 1603–1609; c) T. Ikai, S. Miyoshi, K. Oki, R. Saha, Y. Hijikata, E. Yashima, *Angew. Chem., Int. Ed.* **2023**, *62*, e202301962.

6. ^{1}H and ^{13}C NMR Spectral Data

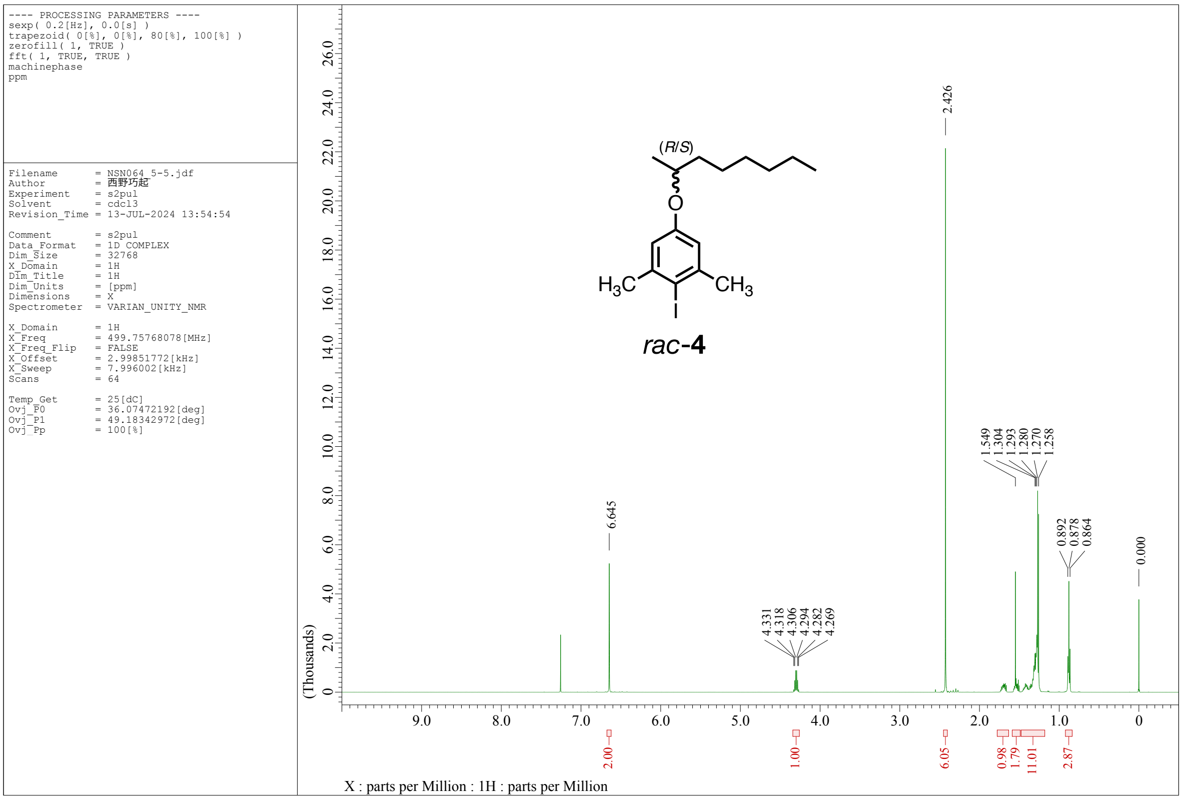

Figure S30. ^{1}H NMR (500 MHz, $CDCl_3$, 25 °C) spectrum of *rac*-**4**.

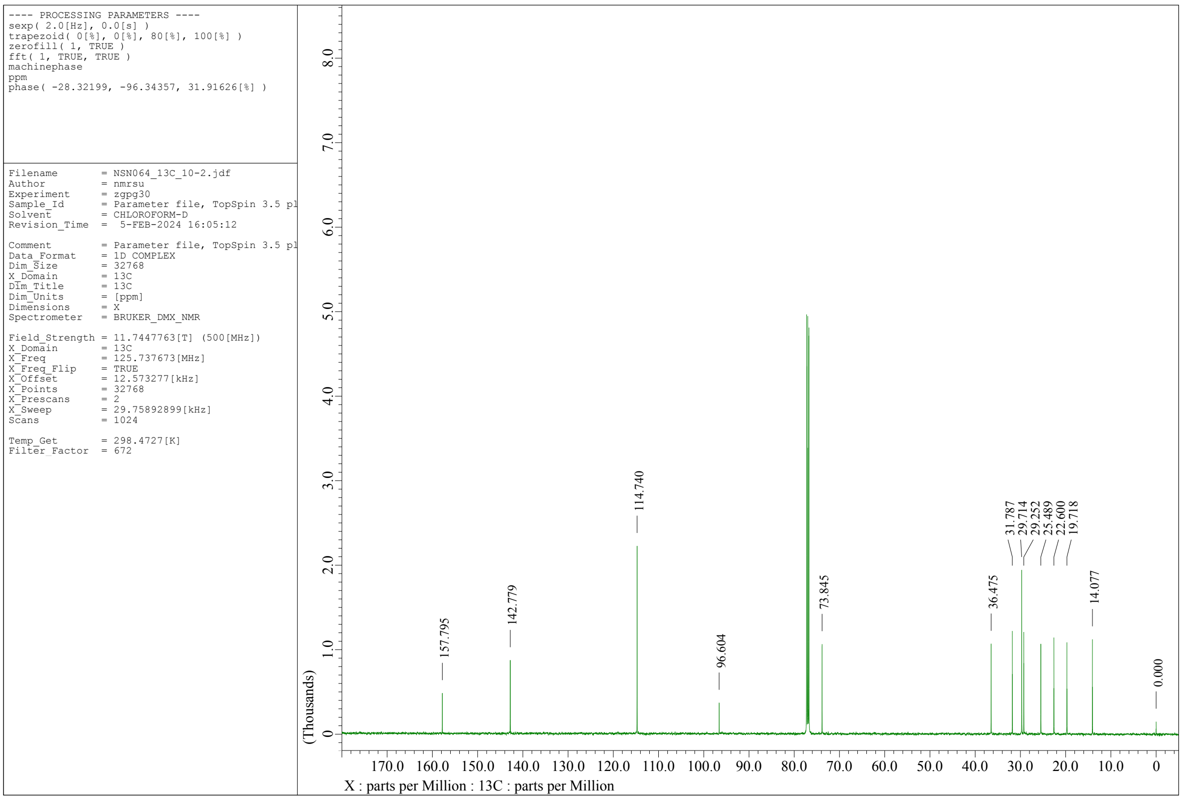

Figure S31. ^{13}C NMR (126 MHz, $CDCl_3$, 25 °C) spectrum of *rac*-**4**.

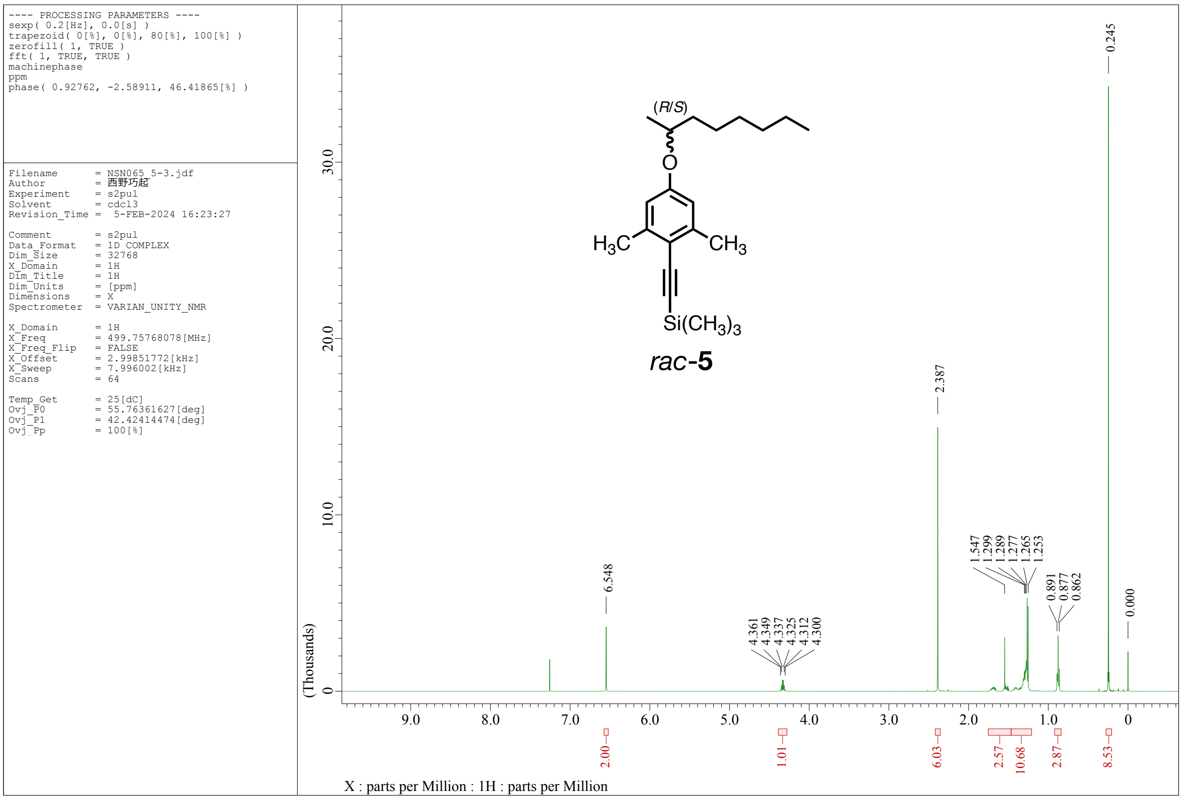

Figure S32. ^{1}H NMR (500 MHz, $CDCl_3$, 25 °C) spectrum of *rac*-**5**.

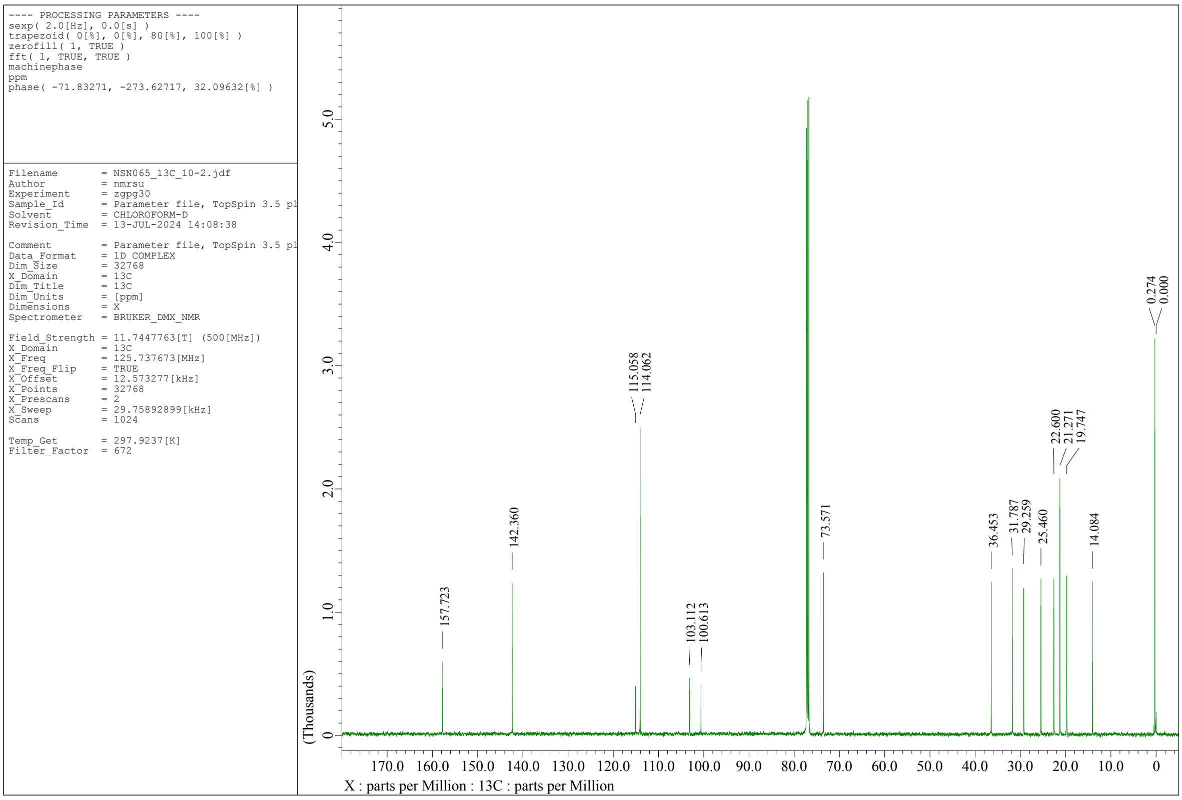

Figure S33. ^{13}C NMR (126 MHz, $CDCl_3$, 25 °C) spectrum of *rac*-**5**.

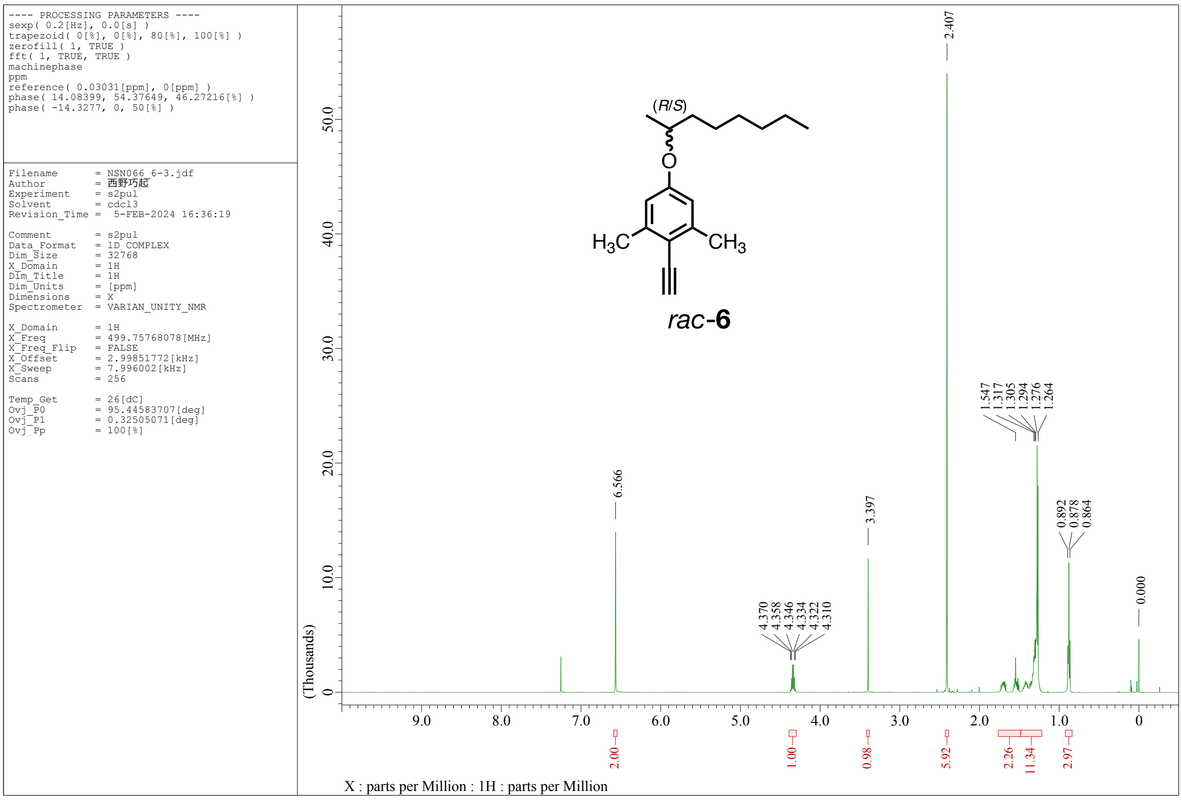

Figure S34. ^{1}H NMR (500 MHz, $CDCl_3$, 25 °C) spectrum of *rac*-**6**.

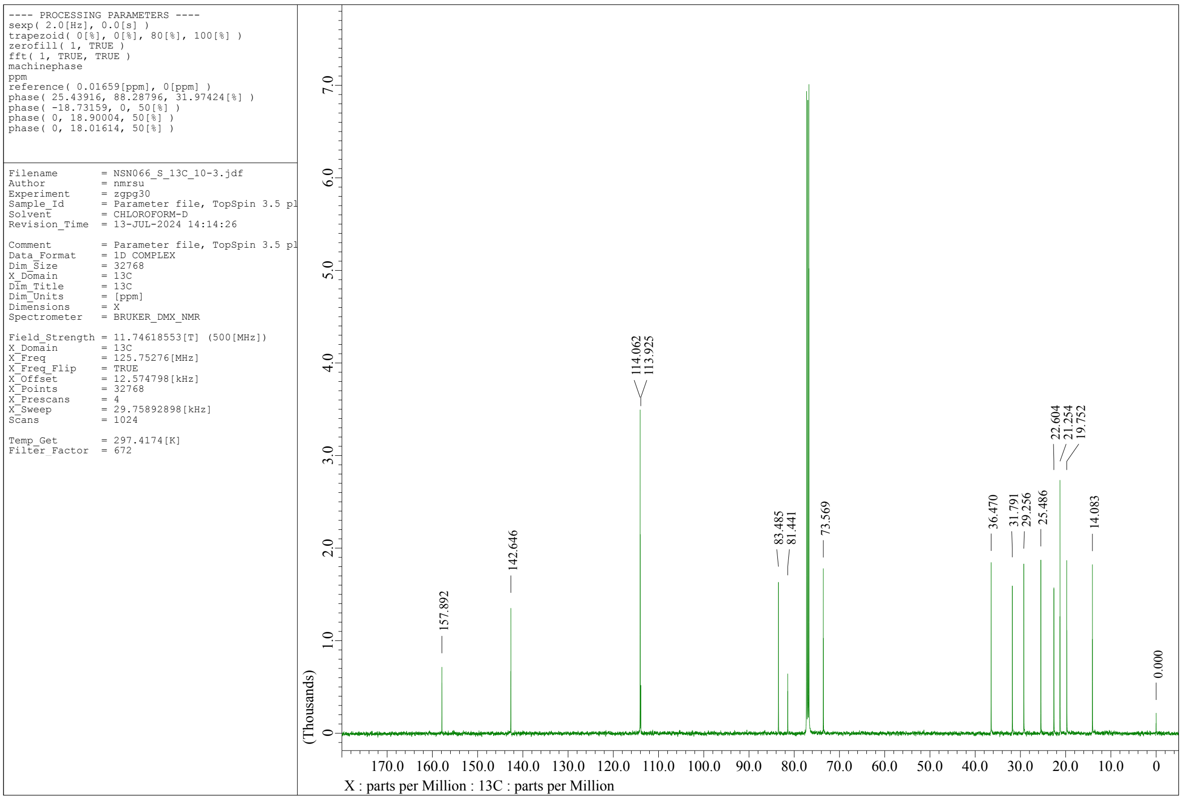

Figure S35. ^{13}C NMR (126 MHz, $CDCl_3$, 25 °C) spectrum of *rac*-**6**.

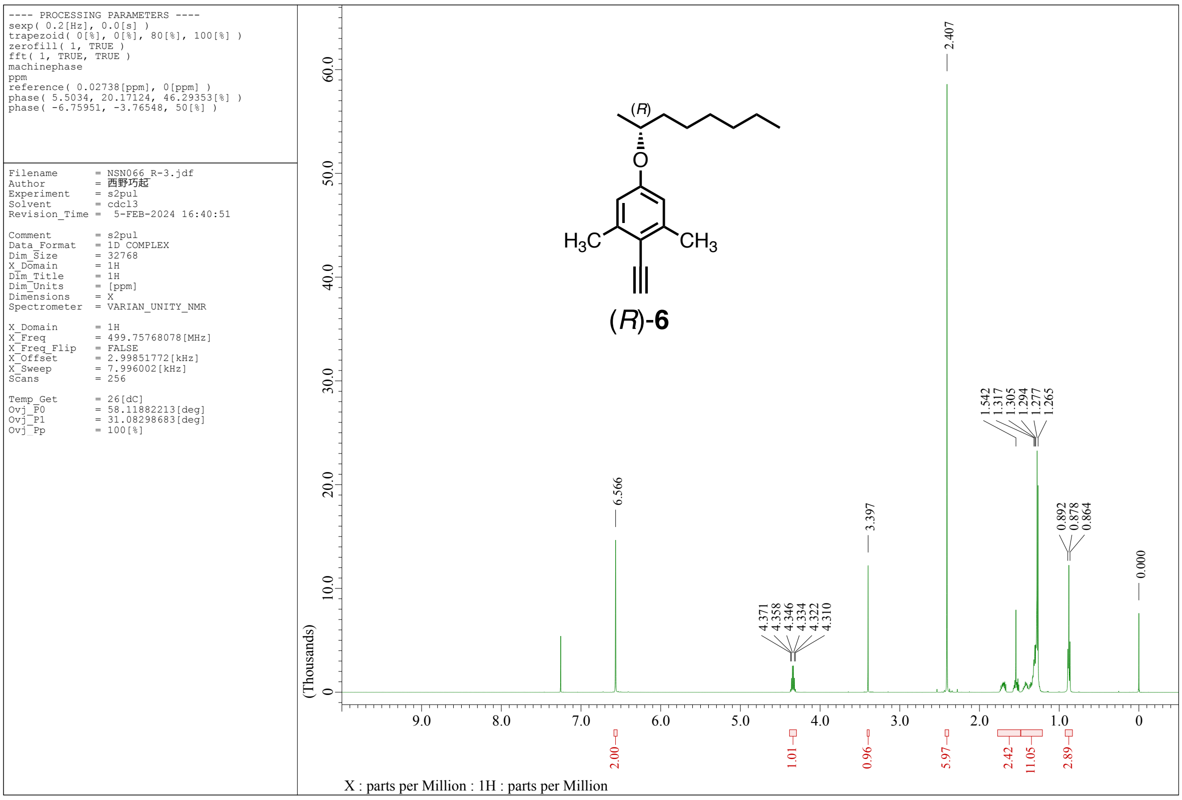

Figure S36. ^{1}H NMR (500 MHz, $CDCl_3$, 25 °C) spectrum of (*R*)-**6**.

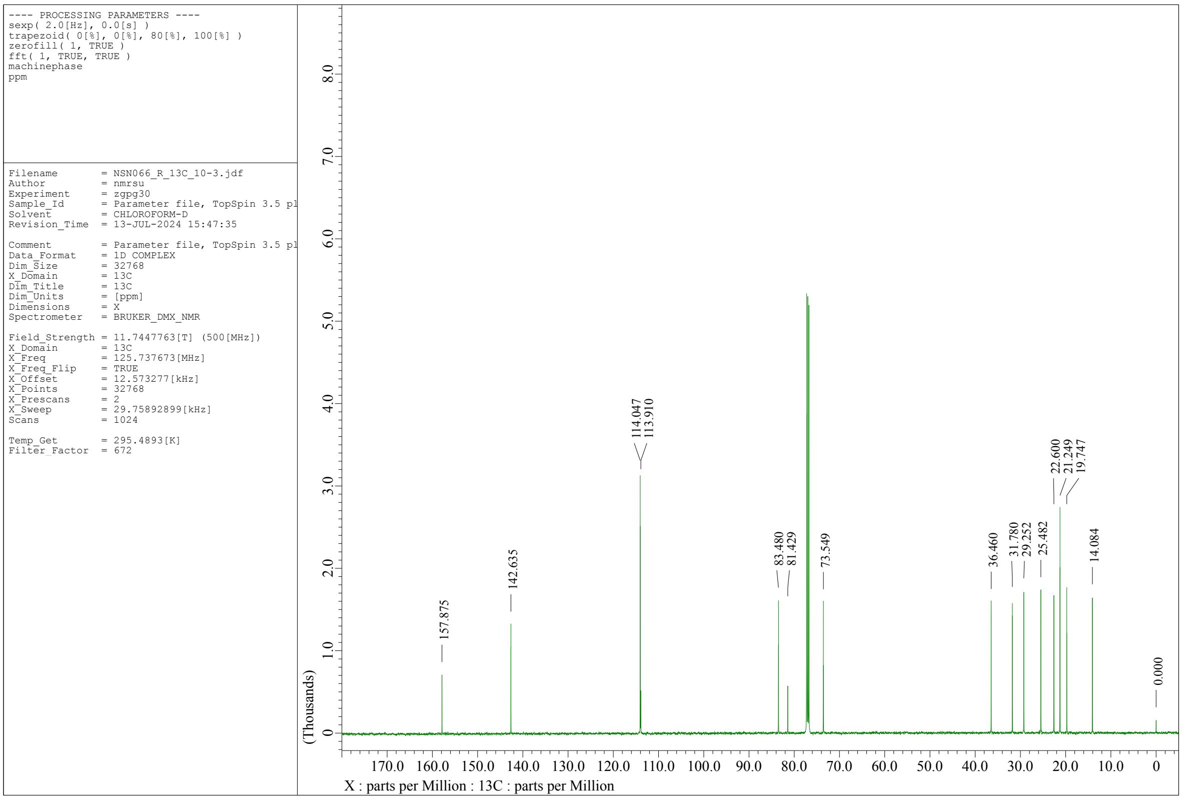

Figure S37. ^{13}C NMR (126 MHz, $CDCl_3$, 25 °C) spectrum of (*R*)-**6**.

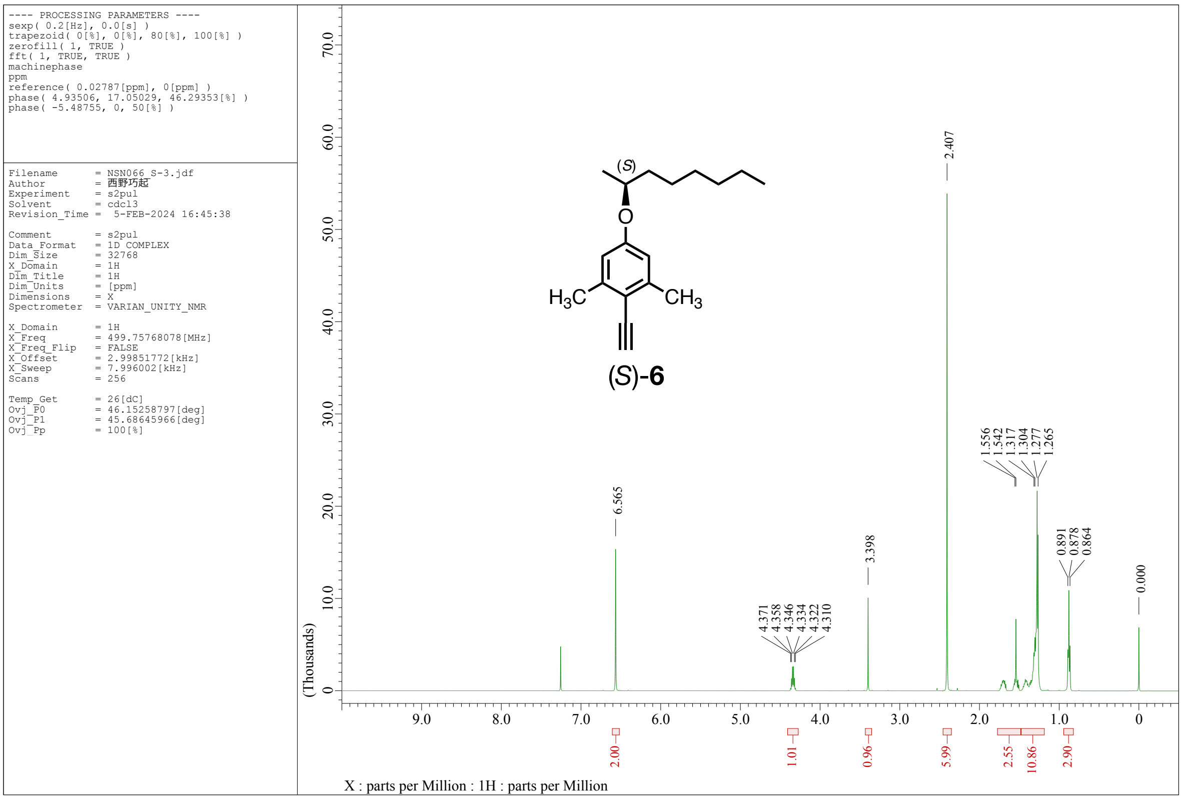

Figure S38. ^{1}H NMR (500 MHz, $CDCl_3$, 25 °C) spectrum of (*S*)-**6**.

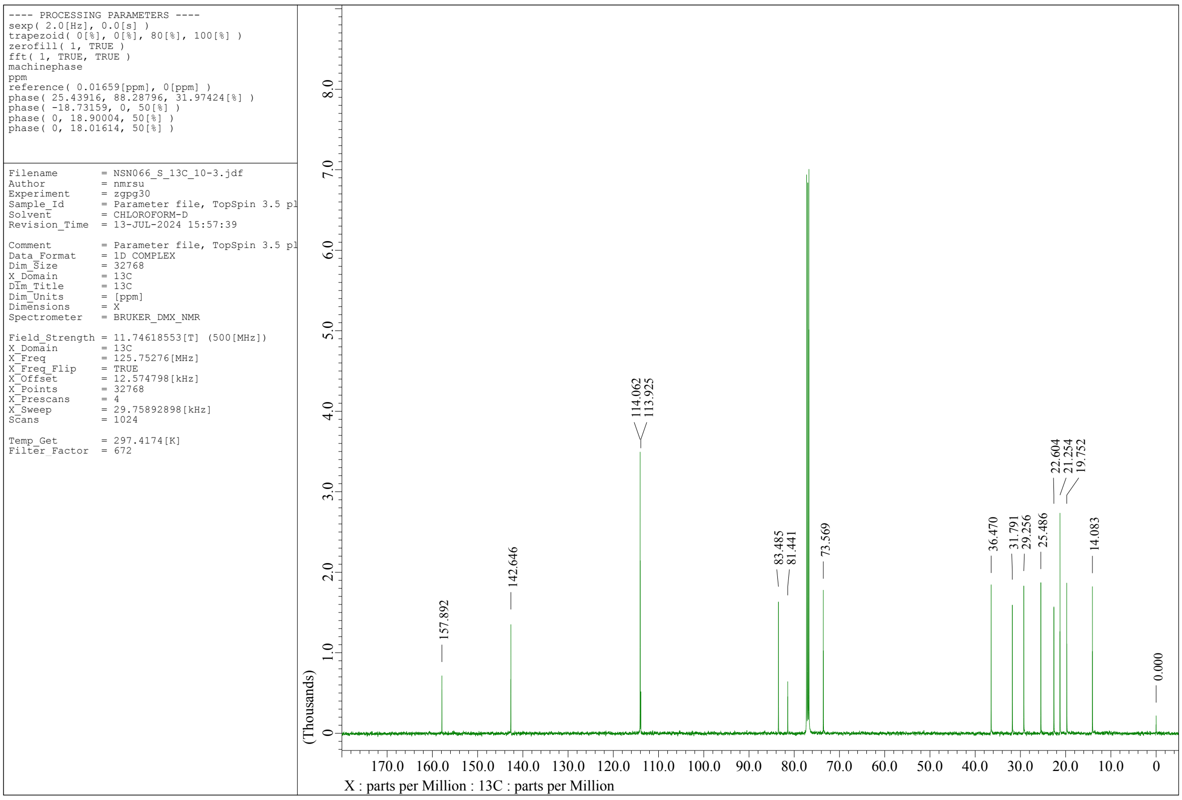

Figure S39. ^{13}C NMR (126 MHz, $CDCl_3$, 25 °C) spectrum of (*S*)-**6**.

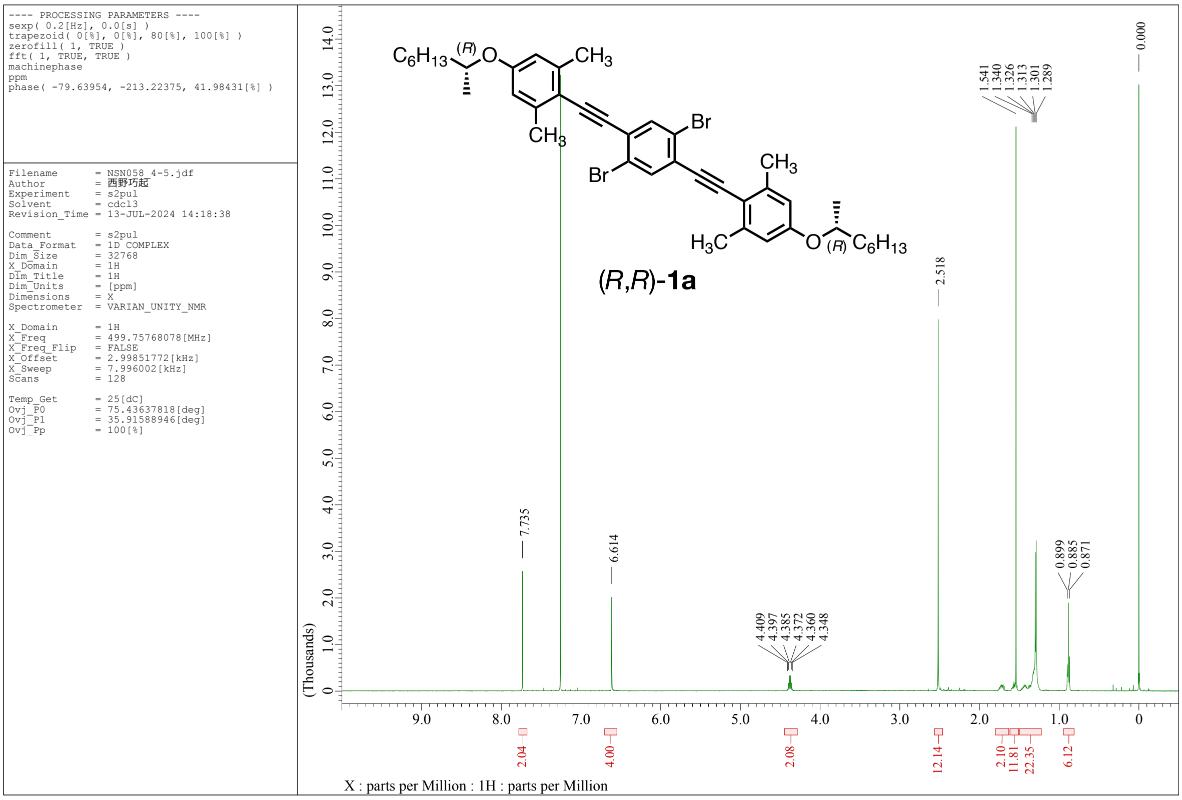

Figure S40. ^{1}H NMR (500 MHz, $CDCl_3$, 25 °C) spectrum of (*R*,*R*)-**1a**.

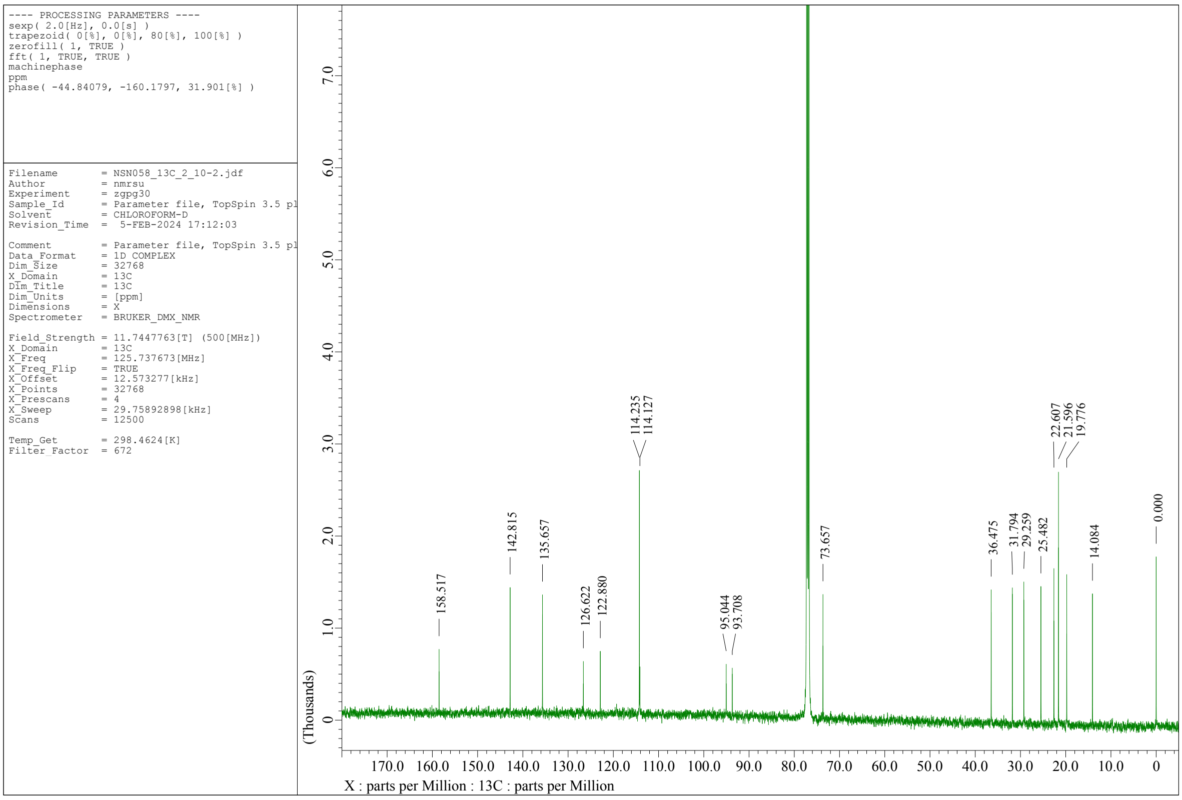

Figure S41. ^{13}C NMR (126 MHz, $CDCl_3$, 25 °C) spectrum of (*R*,*R*)-**1a**.

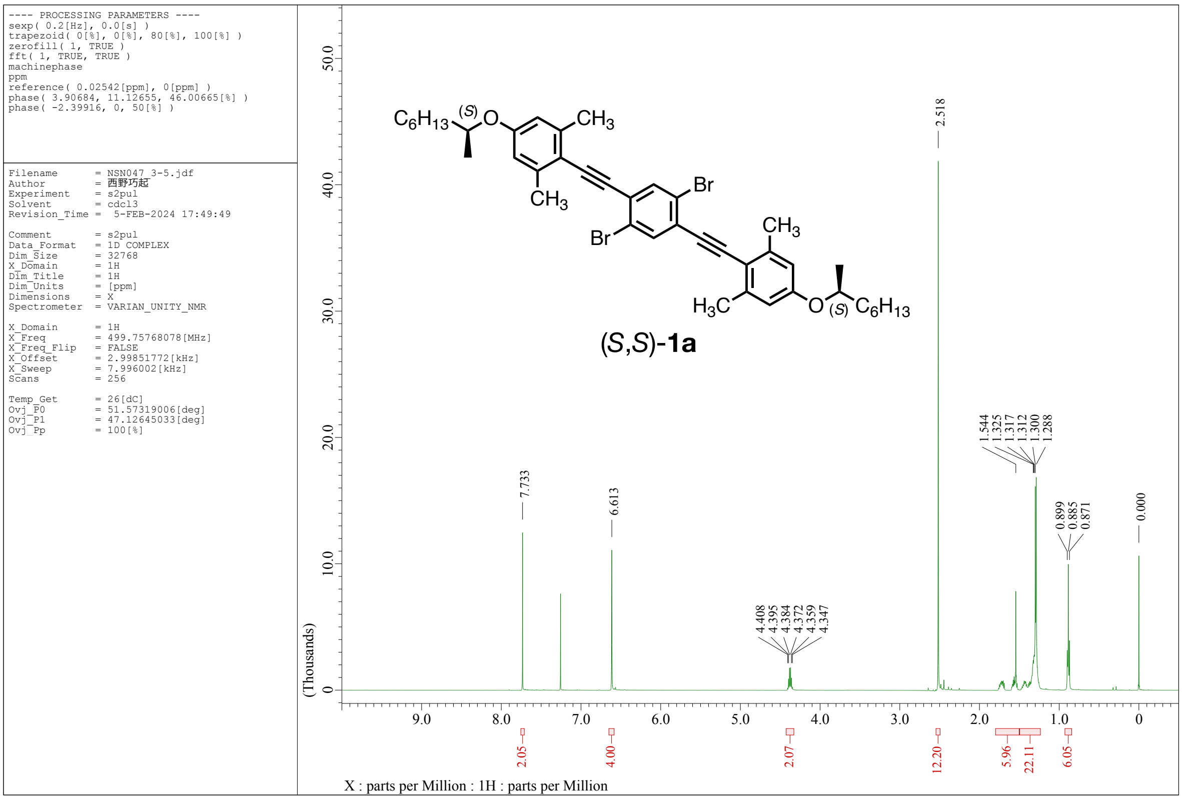

Figure S42. ^{1}H NMR (500 MHz, $CDCl_3$, 25 °C) spectrum of (*S*,*S*)-**1a**.

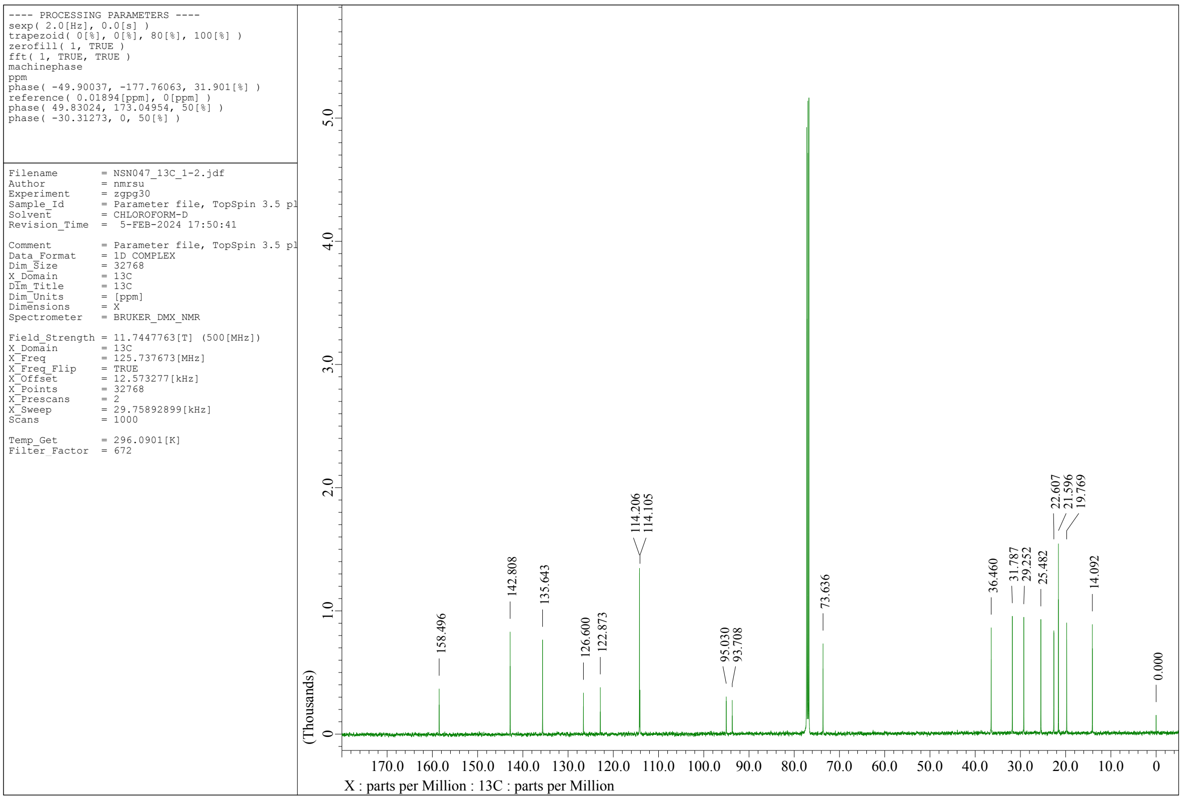

Figure S43. ^{13}C NMR (126 MHz, $CDCl_3$, 25 °C) spectrum of (*S*,*S*)-**1a**.

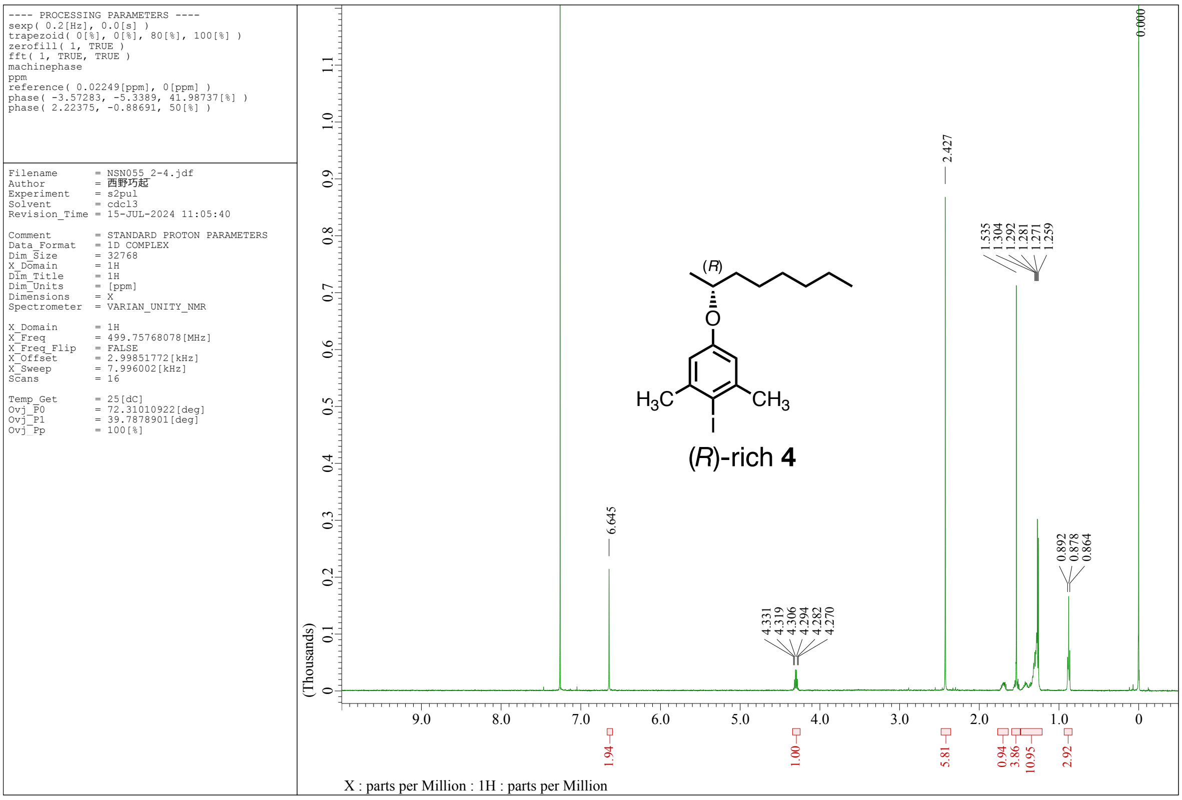

Figure S44. ^{1}H NMR (500 MHz, $CDCl_3$, 25 °C) spectrum of (*R*)-rich **4**.

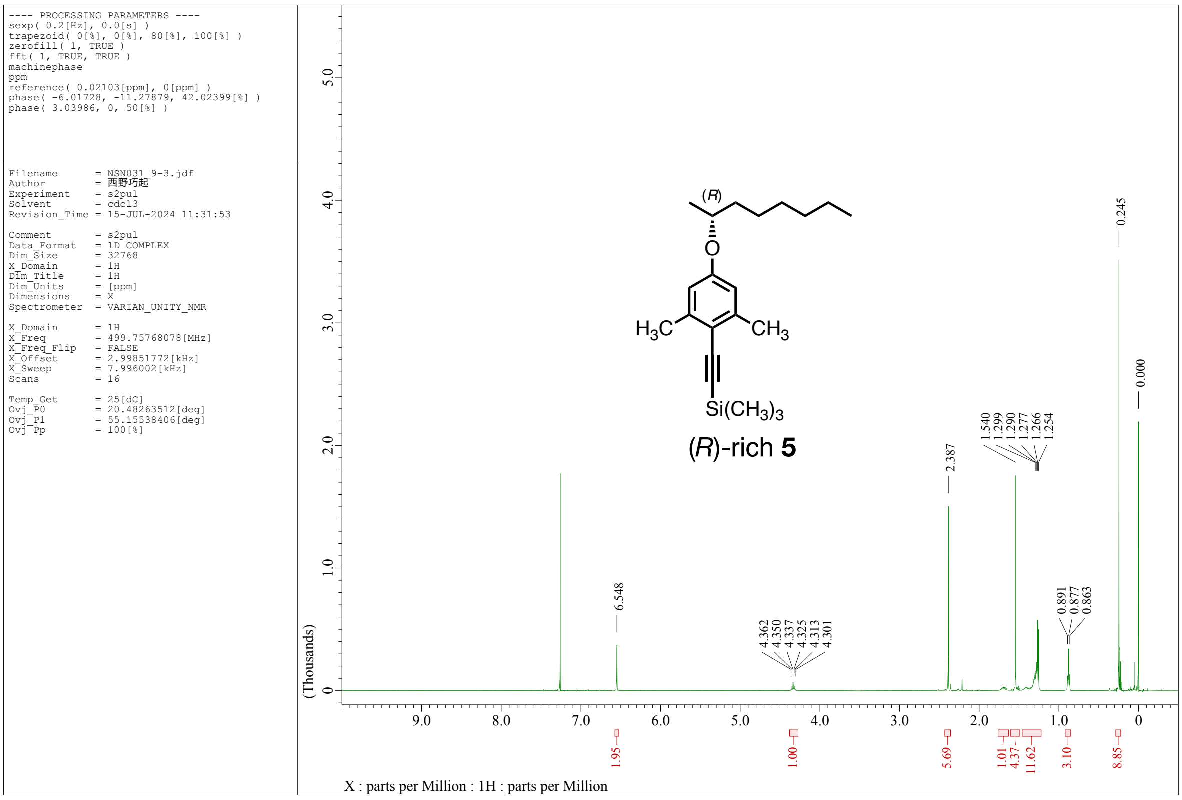

Figure S45. ^{1}H NMR (500 MHz, $CDCl_3$, 25 °C) spectrum of (*R*)-rich **5**.

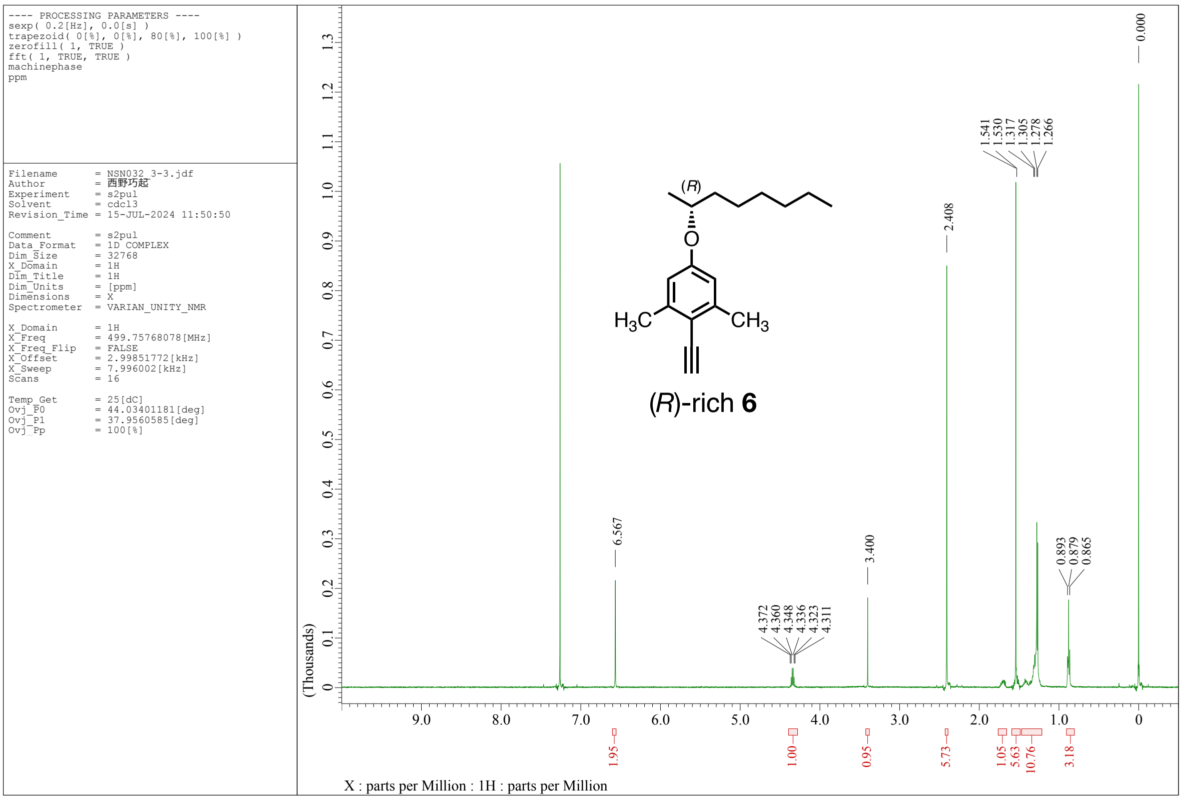

Figure S46. ^{1}H NMR (500 MHz, $CDCl_3$, 25 °C) spectrum of (*R*)-rich **6**.

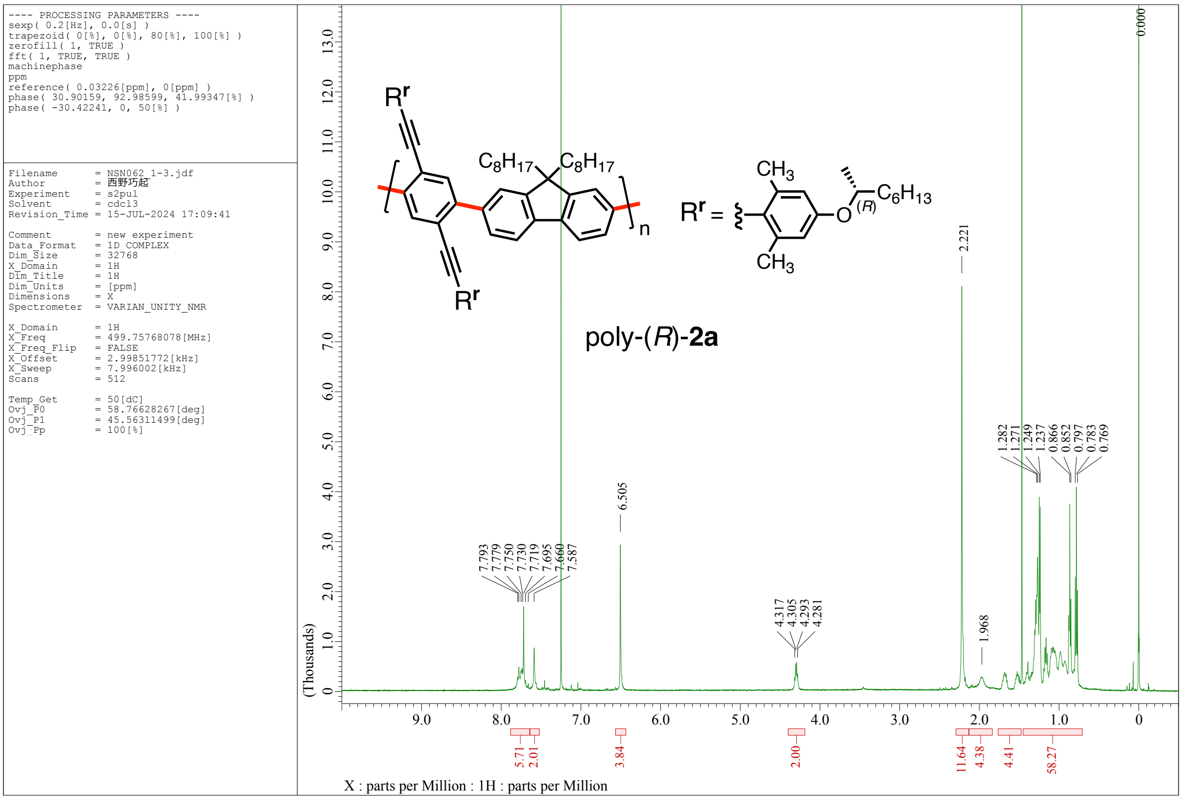

Figure S47. ^{1}H NMR (500 MHz, $CDCl_3$, 50 °C) spectrum of poly-(*R*)-**2a**.

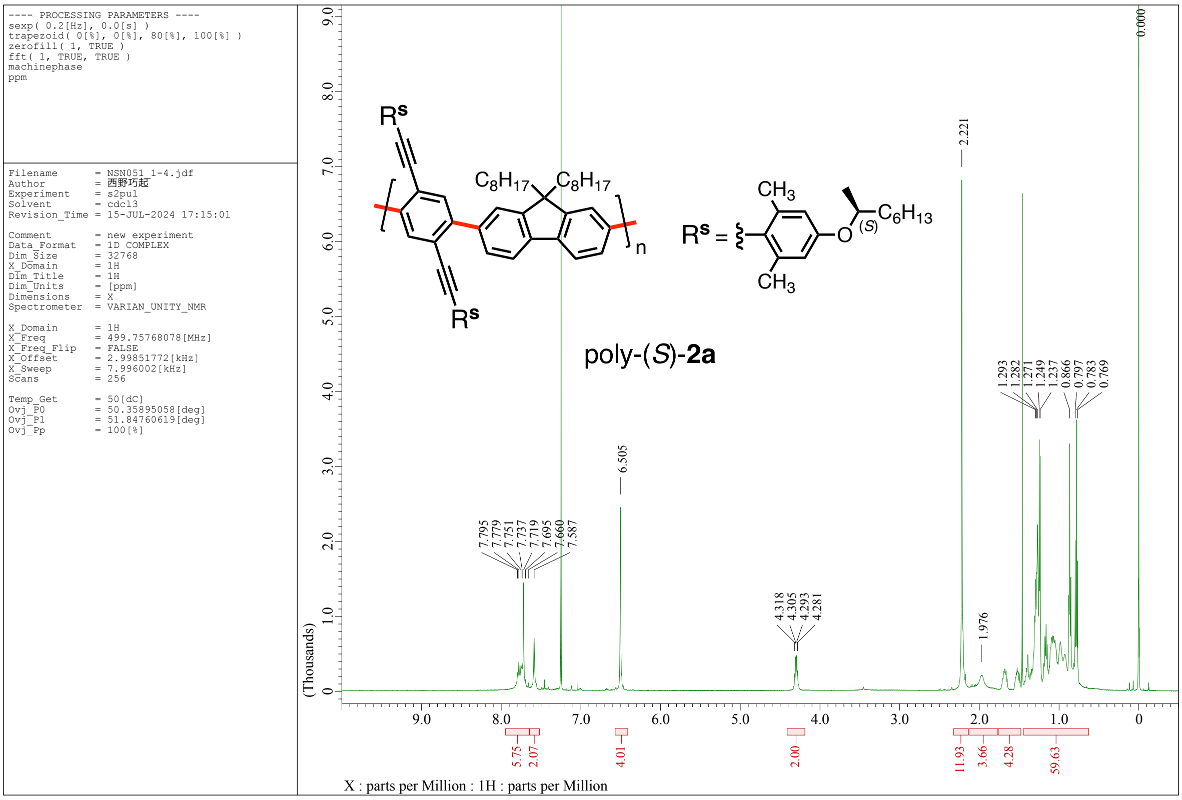

Figure S48. ^{1}H NMR (500 MHz, $CDCl_3$, 50 °C) spectrum of poly-(*S*)-**2a**.

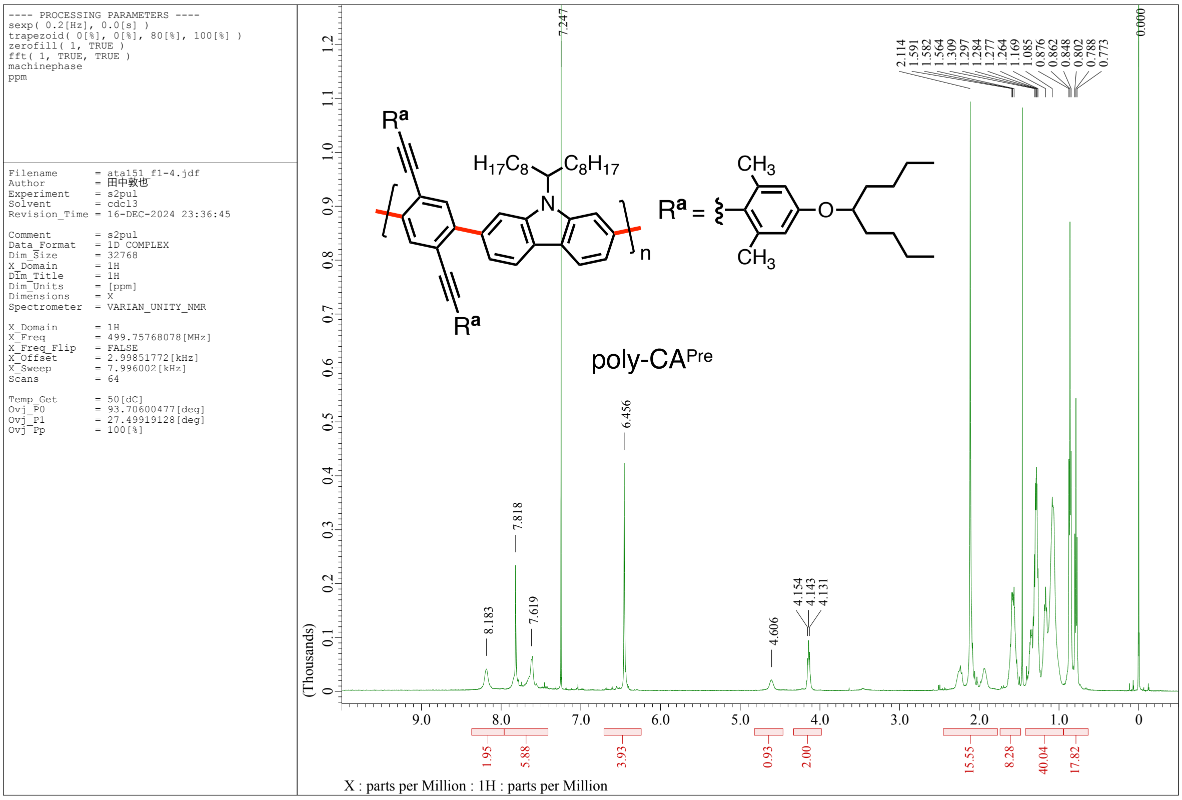

Figure S49. ^{1}H NMR (500 MHz, $CDCl_3$, 50 °C) spectrum of poly-CA^{Pre}.

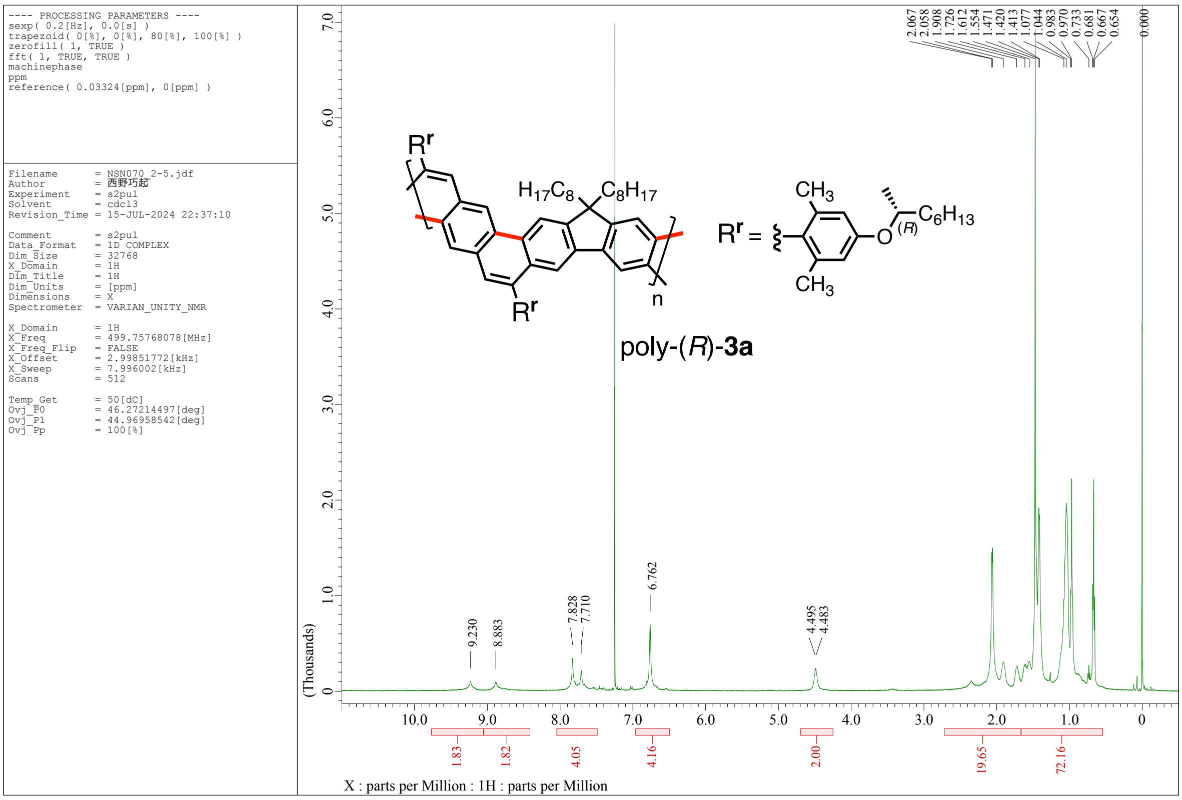

Figure S50. ^{1}H NMR (500 MHz, $CDCl_3$, 50 °C) spectrum of poly-(*R*)-**3a**.

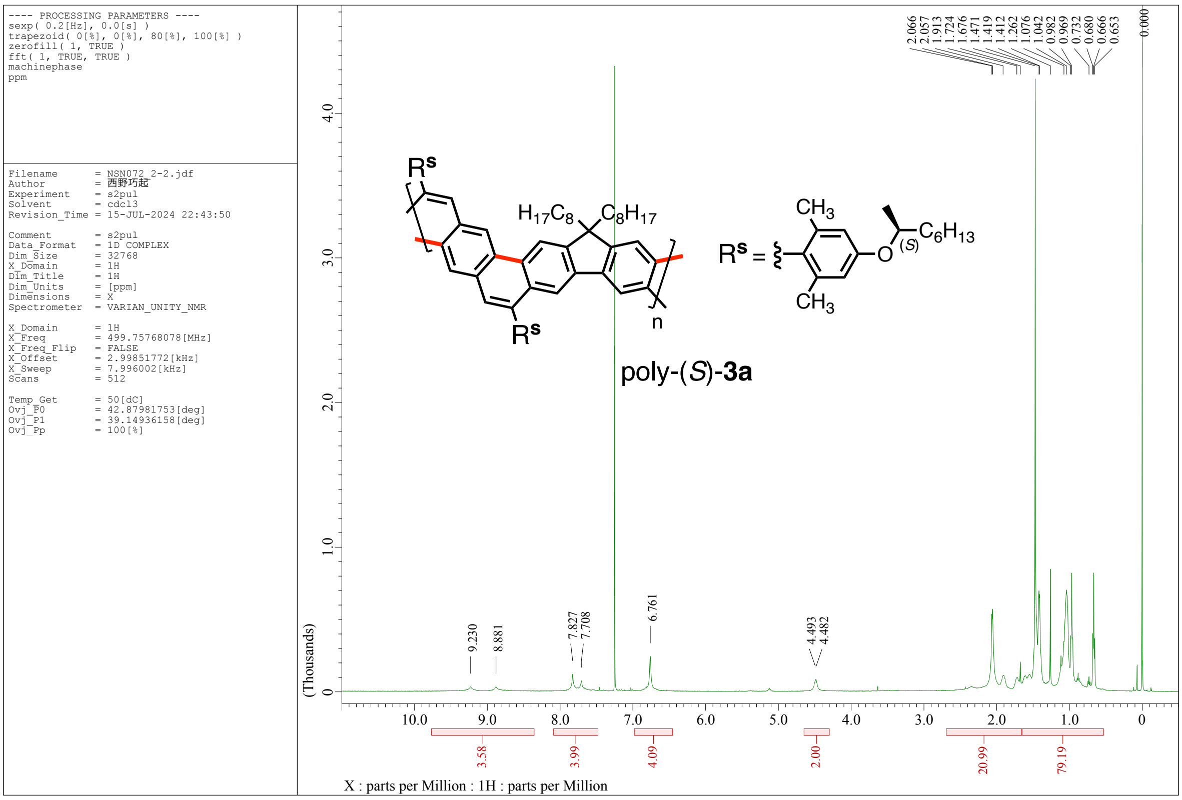

Figure S51. ^{1}H NMR (500 MHz, $CDCl_3$, 50 °C) spectrum of poly-(*S*)-**3a**.

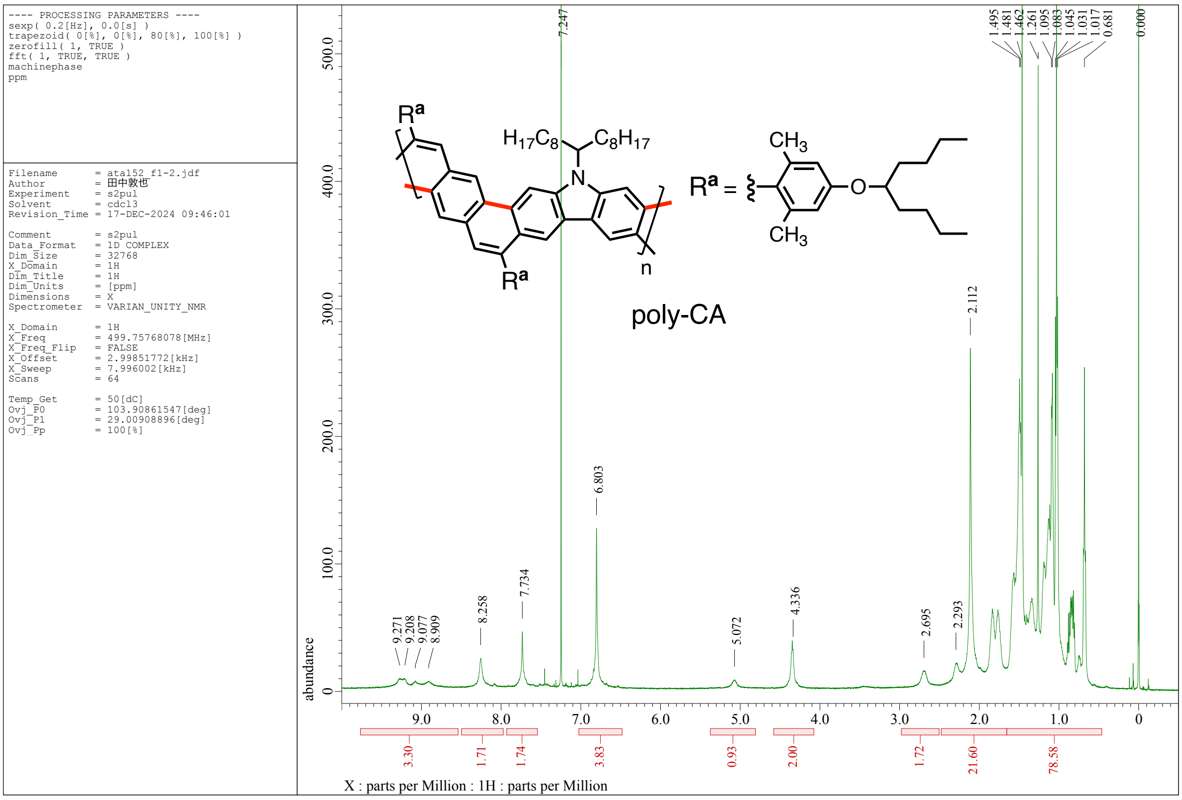

Figure S52. ^{1}H NMR (500 MHz, $CDCl_3$, 50 °C) spectrum of poly-CA.
